# *FGF5* methylation is a sensitivity marker of esophageal squamous cell carcinoma to definitive chemoradiotherapy

**DOI:** 10.1038/s41598-019-50005-6

**Published:** 2019-09-16

**Authors:** Jun Iwabu, Satoshi Yamashita, Hideyuki Takeshima, Takayoshi Kishino, Takamasa Takahashi, Ichiro Oda, Kazuo Koyanagi, Hiroyasu Igaki, Yuji Tachimori, Hiroyuki Daiko, Hidetsugu Nakazato, Kazuhiro Nishiyama, Yi-Chia Lee, Kazuhiro Hanazaki, Toshikazu Ushijima

**Affiliations:** 1 Division of Epigenomics, National Cancer Center Research Institute, Tokyo, Japan; 2 Division of Endoscopy, National Cancer Center Hospital, Tokyo, Japan; 3 Division of Esophageal Surgery, National Cancer Center Hospital, Tokyo, Japan; 4 Department of Internal Medicine, College of Medicine, National Taiwan University, Taipei, Taiwan; 5 Department of Surgery, Kochi Medical School, Kochi, Japan

**Keywords:** Oesophageal cancer, Tumour biomarkers, Epigenomics, Predictive markers

## Abstract

Definitive chemoradiotherapy (dCRT) is the major treatment for esophageal squamous cell carcinoma (ESCC), and prediction of the response to dCRT is important so as not to miss an opportunity to cure an ESCC. Nevertheless, few validated markers are available. Here, we aimed to identify a highly reproducible marker using multi-layer omics analysis. 117 ESCC samples from 67 responders and 50 non-responders were divided into screening, validation, and re-validation sets. In the screening cohort (n = 41), somatic mutations in 114 genes showed no association with dCRT response. Genome-wide DNA methylation analysis using Infinium HumanMethylation450 BeadChip array identified four genic regions significantly associated with dCRT response. Among them, *FGF5* methylation was validated to be associated with dCRT response (n = 34; *P* = 0.001), and further re-validated (n = 42; *P* = 0.020) by bisulfite-pyrosequencing. The sensitivity and specificity in the combined validation and re-validation sets (n = 76) were 45% and 90%, respectively, by using the cut-off value established in the screening set, and *FGF5* methylation had predictive power independent from clinicopathological parameters. In ESCC cell lines, *FGF5* promoter methylation repressed its expression. *FGF5* expression was induced by cisplatin (CDDP) treatment in three unmethylated cell lines, but not in two methylated cell lines. Exogenous *FGF5* overexpression in a cell line with its methylation conferred resistance to CDDP. In non-cancerous esophageal tissues, *FGF5* was not expressed, and its methylation was present in a small fraction of cells. These results showed that *FGF5* methylation is a validated marker for ESCC sensitivity to dCRT.

## Introduction

Definitive chemoradiotherapy (dCRT) is a treatment strategy for patients with locally advanced esophageal squamous cell carcinoma (ESCC) that is frequently adopted as an alternative to surgical resection^1,2^. In Japan, dCRT is indicated for patients with resectable stage II/III ESCC who refuse surgery with tolerable complete response rates of 15–37% while it is 62.6% by neoadjuvant chemotherapy followed by surgery^3,4^. One major reason for the low complete response rate of dCRT is the high proportion (40 to 60%) of patients who have resistance to dCRT^5–7^. Patients who have a residual tumor after dCRT have to receive salvage surgery, and unfortunately, the mortality of salvage surgery is very high (8–15%)^8,9^. At the same time, patients who show complete response to dCRT do not need to receive surgery or further chemotherapy. Therefore, there is a strong clinical need to predict the sensitivity of ESCCs to dCRT.

To predict the response of an ESCC patient to dCRT, multiple exploratory studies using molecular markers have been conducted. Indeed, associations of the response with a genotype (*GALNT14*)^10^, gene expression (*MDM2*, *LOC285194*, and *SIM2*)^11–13^, and gene methylation (*ZNF695*)^14^ have been reported. However, most of the reports, except for *ZNF695*, have not been validated in an independent cohort of patients, and their validity in general is still unclear. In addition, the sensitivity and specificity to predict responders to dCRT are still unsatisfactory. For example, *ZNF695* has a sensitivity of 39% and specificity of 90% to predict responders, and needs further improvement.

In the present study, we conducted multi-layer omics analysis to isolate a novel biomarker that can predict sensitivity of an ESCC to dCRT with high specificity, and validated and re-validated the marker in independent sample sets. We also analyzed how methylation of the marker gene functions in the sensitivity to dCRT.

## Methods

### Clinical samples and patient profiles

125 ESCC were collected from 125 ESCC patients from 2010 to 2016 at the National Cancer Center Hospital, Japan. ESCCs were histologically confirmed, and the patients were at cStage Ib-IV according to the 7th edition of the TNM classification. The response to dCRT was determined based on the endoscopic findings of the primary tumor after each course of chemotherapy using the modified criteria of the 10th edition of the Japanese Society for Esophageal Diseases^15^. A responder was defined as a patient with disappearance of the primary tumor without pathological residual lesions in biopsy specimens.

The samples were stored in RNAlater (Life Technologies, Carlsbad, CA, USA) at −80 °C until extraction of DNA. Eight ESCC specimens were excluded from the analysis because the cancer cell fraction measured by a DNA methylation marker was less than 20%^16^. As a result, 117 ESCC samples were used for this study. Fifteen samples analyzed by HumanMethylation450 in our previous study^14^ were assigned to the screening set. The other 102 samples were randomly assigned to either the screening set (26 samples), validation set (34 samples), or re-validation set (42 samples). The patient profiles are shown in Supplementary Table S1, and the sample overlap with our previous studies is shown in Supplementary Table S2 ^14,17,18^.

Twelve normal esophageal mucosa samples were collected endoscopically from adults who underwent cancer screening at the National Taiwan University Hospital from September 2008 to April 2013. The 12 samples were classified into three risk groups based on the exposure to lifestyle risk factors (alcohol drinking, betel quid chewing, and cigarette smoking) and healthy/cancer statuses as previously described^19^.

### Cell lines and their treatment

Human ESCC cell lines, KYSE-30, 50, 140, 170, 180, 220, 270, 410, 450, 510, and 520 were obtained from the Japanese Collection of Research Bioresources (JCRB) Cell Bank (Ibaragi, Osaka, Japan)^20^. TE-15 was obtained from Riken Cell Bank (Tsukuba, Ibaraki, Japan)^21^. KYSE cell lines and TE-15 were cultured in 50%/50% mixture of RPMI1640/Ham’s F12 medium containing 2% (v/v) FBS and RPMI1640 medium containing 10% (v/v) FBS, respectively.

For 5-aza-2′-deoxycytidine (5-aza-dC) treatment, KYSE-170 and KYSE-180 were seeded at a density 1 × 10^5^ cells per 10-cm plate on day 0, and were treated on days 1 and 3. The concentration of 5-aza-dC for KYSE-170 and KYSE-180 was adjusted to 0, 0.1, 0.3, 1, and 3 µM, and 0, 1, 3, 10 and 30 µM, respectively, and the cells were collected on day 5. KYSE-170, 180, 270, 410, and 450 were seeded at a density 1.5 × 10^4^, 1.5 × 10^4^, 3 × 10^4^, 4 × 10^3^, and 5 × 10^4^, respectively, per 24 well plate on day 0. These cell lines were treated with cisplatin on day 1. The cells were collected on days 1, 2 and 4. To analyze the effect of *FGF5* overexpression, KYSE-180 was seeded at a density of 5 × 10^3^ per well in a 96-well plate on day 0, and was transiently transfected with pBApo-CMV empty or *FGF5* (long variant) by a Lipofectamine 3000 Transfection Kit (Invitrogen, Eugene, OR, USA) on day 1. These cell lines were treated with CDDP on day 2. Cells were collected on day 5.

### Extraction of DNA and RNA

Genomic DNA was extracted by the phenol/chloroform method and quantified using a Quant-iT PicoGreen dsDNA Assay Kit (Invitrogen, Eugene, OR, USA). Total RNA was extracted by ISOGEN (Nippon Gene, Tokyo, Japan).

### Mutation analysis

Target sequencing was performed using three panels of genes (CP1, repair, and SWI/SNF panel). The CP1 panel contained 55 genes in 226 fragments as described previously^22^. The repair panel contained 46 genes in 1,335 fragments (Supplementary Table S3). The SWI/SNF panel contained 18 genes in 672 fragments as described previously^23^. A total of 114 genes were analyzed because five genes were duplicated in two panels. A DNA library was prepared for each panel by multiplex polymerase chain reaction (PCR), and the library was sequenced using an Ion Proton Sequencer.

### Genome-wide DNA methylation analysis

A genome-wide screening of differentially methylated CpG sites was conducted using an Infinium HumanMethylation450 BeadChip array that interrogates 485,512 methylation sites (Illumina, San Diego, CA, USA). We analyzed the 470,870 CpG sites on autosomes after excluding probes on the sex chromosomes and non-CpG probes. The methylation level of each CpG site was obtained as a β-value, which ranged from 0 (completely unmethylated) to 1 (completely methylated). A corrected β-value was calculated using a measured β-value and the fraction of cancer cells in a sample [A corrected β-value = measured β-value × 100/(the fraction of cancer cells in a sample) (%)]^16^. Unsupervised hierarchical clustering analysis was performed using R 3.51 with the Heatplus package from Bioconductor^24^. The analysis was conducted for all CpG sites, all CpG islands, regions near transcription start sites (TSS200), and enhancers.

### Gene-specific DNA methylation analysis

One µg genomic DNA was treated with sodium bisulfite, and eluted into 50 µl elution buffer using an innuCONVERT Bisulfite Basic Kit (Analytik Jena AG, Jena, Germany). Bisulfite pyrosequencing was performed using PyroMark (Qiagen). The measured methylation level was corrected using the fraction of cancer cells in a sample^16^. Deep bisulfite sequencing was performed using Ion PGM (Thermo Fisher Scientific) with bisulfite-modified DNA and primers used for the bisulfite-pyrosequencing. The PCR primers for bisulfite-pyrosequencing and measurement of cancer cell fraction are listed in Supplementary Table S4.

### Quantitative RT-PCR

cDNA was synthesized from total RNA using SuperScript IV Reverse Transcriptase (Invitrogen, Eugene, OR, USA). Real-time PCR was performed using cDNA samples, specific primers (Supplementary Table S4), EvaGreen (Biotium, Fremont, CA, USA), and CFX Connect Real-Time PCR Detection System (Bio-Rad, Hercules, CA, USA). The copy number of a target gene in a sample was measured by comparing its amplification to those of the control samples with known copy numbers. The measured copy number of a target gene was normalized to that of *GAPDH*. All of the analyses were performed in triplicate.

### Statistical analysis

Fisher’s exact test and the Mann-Whitney *U* test were used to evaluate the difference in characteristics between responders and non-responders to dCRT. Differences in the corrected methylation levels were evaluated by the Mann-Whitney *U* test. The one-way analysis of variance test was used to evaluate the distribution of methylation levels among cohorts. The Jonckheere-Terpstra trend test was used to test an increasing trend of DNA methylation levels according to cancer risk levels. The odds ratios (ORs) and 95% confidence interval (95% CI) were calculated in a univariate analysis. The factors affecting the response to dCRT were tested by a multivariate logistic regression analysis. All statistical analysis was conducted by PASW statistics version 18.0.0 (IBM, Armonk, NY, USA).

### Ethics approval and consent to participate

The study was performed according to ethics approval and consent. The study was approved by the Institutional Review Boards of National Cancer Center, Japan (Reference No. 2010-094) and  National Taiwan University Hospital (Reference No. 200806039R). The study was performed in accordance with the Declaration of Helsinki.

### Consent for publication

Informed consent for publication was obtained from all participants.

## Results

### No association between dCRT response and somatic mutations

**Figure 1:**
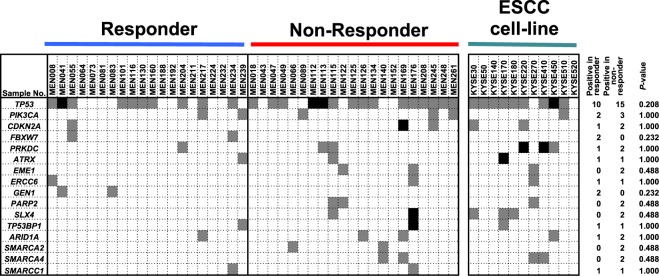
Mutation analysis of the 41 ESCCs and 11 ESCC cell lines. In the 20 responders and 21 non-responders, mutations of 55 cancer-related, 46 repair-related, and 18 SWI/SNF-related genes were analyzed. There was no difference in the incidence of mutations between the two groups. When a sample had two or more mutations in one gene, the box is colored in black. When a sample had one mutation in one gene, the box is colored in gray.

As the first layer of multi-omics analysis, we performed target sequencing of 55 cancer-related genes, 46 DNA repair genes, and 18 chromatin remodeler genes using (1) 20 ESCC samples from responders, (2) 21 ESCC samples from non-responders, and (3) 11 ESCC cell lines. A total of 93 somatic mutations were identified in the 41 ESCC samples (Supplementary Table S5). *TP53* mutation was detected in 25 of the 41 patients, being in the reported range of its mutation incidence. However, the incidence was not different between the responders and non-responders (Fig. 1). The other genes had a low frequency of mutations, and there were no significant differences between the responders and non-responders.

### Isolation of methylation marker genes in the screening set

As the second layer of multi-omics analysis, a genome-wide DNA methylation analysis was performed using an Infinium HumanMethylation450 BeadChip array. To the above 41 ESCC samples analyzed for the mutations, we added three samples of peripheral leukocytes and 12 samples of normal esophageal mucosae. First, from the 470,870 CpG sites on autosomes, we selected 126,963 CpG sites unmethylated (β value < 0.2) in normal esophageal mucosae. Using the 126,963 CpG sites, we explored whether responders and non-responders fell into specific clusters obtained by unsupervised hierarchical clustering analysis. However, there was no significant association between any cluster and responders/non-responders using all CpG sites, those in CpG islands, those in transcription start sites (TSS200) in CpG islands, and those in enhancers (Supplementary Fig. S1).

**Figure 2:**
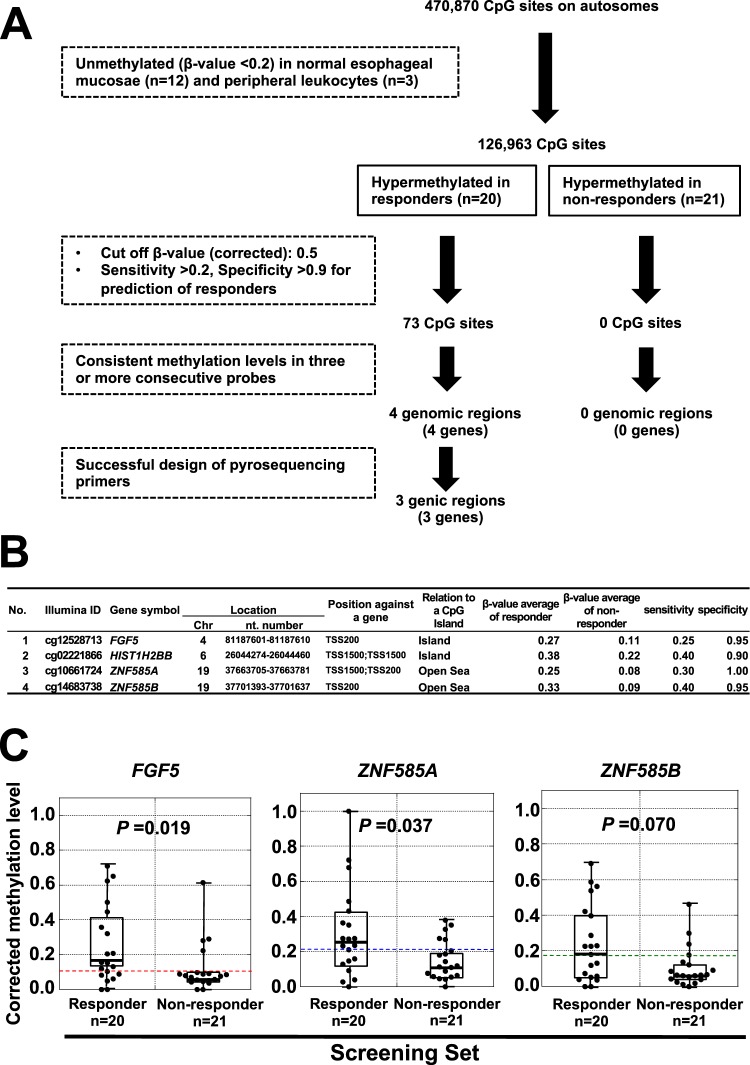
Isolation of three candidate genes in the screening set. (**A**) Workflow of the screening. Details are explained in the text. (**B**) Six genomic regions identified by the genome-wide methylation analysis. Since a genomic region had multiple probes, the ID and location of a probe in the center are shown. (**C**) Methylation levels of the three genomic regions measured by pyrosequencing. *FGF5* and *ZNF585A* showed significantly different methylation levels between the responders and non-responders. A corrected methylation level was calculated using the cancer cell fraction in a sample. A horizontal dotted line shows a cut-off value for sensitivity and specificity obtained in this screening set. Whiskers show maximum and minimum methylation levels.

Then, we searched for individual CpG sites differentially methylated between responders and non-responders. From the 126,963 CpG sites, we isolated CpG sites hypermethylated (corrected β-value > 0.5) in responders (73 sites) with a sensitivity >0.2 and a specificity >0.9 (Fig. 2A). On the other hand, no CpG sites were hypermethylated in non-responders under these criteria. Considering the future use of the marker to select patients who will be assigned to dCRT and thus should respond to dCRT with a high probability, we placed emphasis on its specificity, rather than sensitivity. By searching for genomic regions that had three or more consecutive probes, four genomic regions were isolated as hypermethylated in responders (Fig. 2B; Supplementary Fig. S2).

We attempted to design primers for pyrosequencing for the four genomic regions, and successfully designed primers for three regions (*FGF5*, *ZNF585A* and *ZNF585B*) (Fig. 2A, and Supplementary Table S4). Analysis of the methylation levels of the genomic regions showed that *FGF5* and *ZNF585A* had significant difference between responders and non-responders in the screening set (*P* = 0.019 and 0.037) (Fig. 2C). We established cut-off values of 0.10 (*FGF5*) and 0.21 (*ZNF585A*) based on the maximum Youden index (sensitivity + specificity − 1) (Supplementary Fig. S3).

### Validation and re-validation of the methylation markers

**Figure 3:**
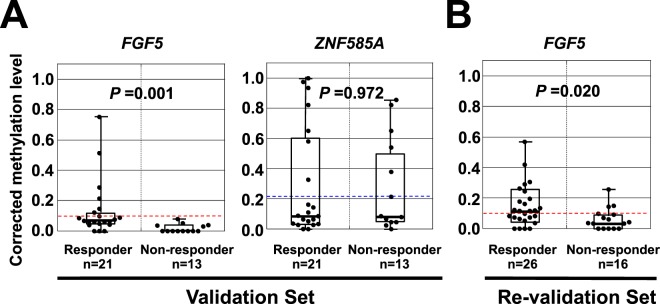
Validation and re-validation of the candidate genes. (**A**) Corrected methylation levels of *FGF5* and *ZNF585A* in the validation set. Differential methylation of only *FGF5* was validated in the 21 responders and 13 non-responders. A horizontal dotted line shows a cut-off value for sensitivity and specificity obtained in the screening set, and whiskers show maximum and minimum methylation levels. (**B**) Corrected methylation levels of *FGF5* in the re-validation set. Differential methylation of *FGF5* was re-validated in the 26 responders and 16 non-responders. A horizontal dotted line shows a cut-off value for sensitivity and specificity obtained in the screening set, and whiskers show maximum and minimum methylation levels.

To validate the association between the *FGF5* and *ZNF585A* methylation levels and response to dCRT in an independent set of samples, we used the validation set (responder, n = 21; non-responder, n = 13). In this set, only *FGF5* methylation levels showed significant difference between the responders and non-responders (*P* = 0.001) (Fig. 3A). A cut-off value of the methylation level established in the screening set achieved a sensitivity of 28% and specificity of 100%.

Since two genes were analyzed in the validation set, the association was further confirmed in an additional independent sample set (re-validation set) (responder, n = 26; non-responder, n = 16). *FGF5* methylation levels showed significant difference between the two groups once again (*P* = 0.020) (Fig. 3B). The response to dCRT was predicted with a sensitivity of 58% and specificity of 81% using the cut-off value established in the screening set. These results in the screening, validation, and re-validation sets demonstrated that *FGF5* methylation was associated with the response to dCRT with a high specificity. The distribution of the methylation levels appeared to be different among the three cohorts, but did not show any statistical difference (*P* = 0.06).

### Independence of FGF5 methylation from the other clinicopathological parameters

The predictive power of *FGF5* methylation in all the sample sets was compared with other clinical factors. Univariate analyses showed that gender, clinical T stage, clinical N stage, clinical M stage, and *FGF5* methylation were significantly associated with the response to dCRT (Table 1). A multivariate logistic regression analysis involving gender, clinical T stage, clinical N stage, clinical M stage, and *FGF5* methylation showed that *FGF5* methylation was an independent predictive factor for the response to dCRT (OR 6.17, 95% CI 2.06–18.34, *P* = 0.001).

**Table 1:** Predictive power of *FGF5* methylation compared with other clinicopathological factors.

Features	Categories	No. of cases			OR	95% CI	*P* value
		Total	Responder	Non-responder			
**Univariate analysis**							
**Age**	≥60	93	56	37	1.79	0.72–4.42	0.207
	<60	24	11	13			
Gender	Male	92	59	33	3.80	1.48–9.75	0.005
	Female	25	8	17			
Location	Cervical, upper	33	19	14	1.02	0.45–2.30	0.966
	Middle, lower	84	48	36			
Radiation dose (Gy)	60	57	32	25	1.09	0.53–2.28	0.811
	50.4	60	35	25			
Clinical T stage	T1b, T2	28	26	2	15.22	3.41–68.03	<0.0001
	T3, T4	89	41	48			
Clinical N stage	N0, N1	64	47	17	4.56	1.19–5.46	0.016
	N2, N3	53	20	33			
Clinical M stage	M0	86	55	31	2.81	1.21–6.55	0.017
	M1	31	12	19			
*FGF5* Methylation	methylated	41	34	7	6.18	2.43–15.71	<0.0001
	unmethylated	75	33	42			
**Multivariate logistic regression analysis**							
Gender	(male/female)	92/25			6.72	1.96–23.07	0.002
Clinical T stage	(T1b or T2/T3 or T4)	28/89			11.19	2.19–57.27	0.004
Clinical N stage	(N0 or N1/N2 or N3)	64/53			1.54	0.59–3.97	0.377
Clinical M stage	(M0 vs. M1)	86/31			3.36	1.13–10.00	0.030
*FGF5* methylation	(methylated/unmethylated)	41/75			6.14	2.06–18.34	0.001

### Functional consequence of FGF5 promoter methylation

**Figure 4:**
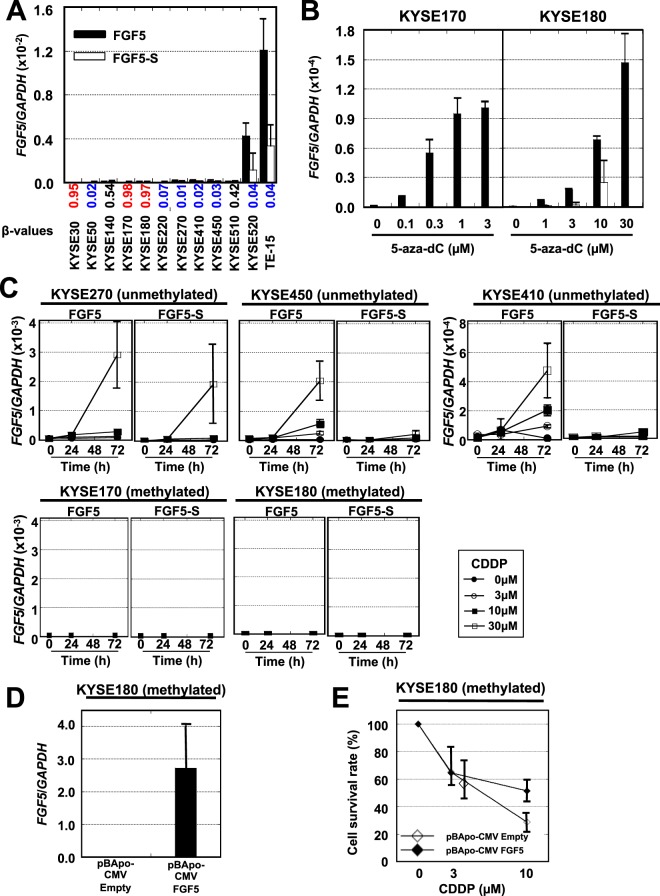
Transcriptional repression of FGF5 by its methylation of promoter CpG island and induction of FGF5 by CDDP treatment. (**A**) Expression levels of *FGF5* and *FGF5-S* variants in 12 ESCC cell lines. Methylation levels obtained by a bead array analysis are also shown (high values in red, and low values in blue). Error bars mean SD (n = 3). (**B**) Re-expression of *FGF5* in KYSE-170 and KYSE-180 cells by treatment with a DNA demethylating agent, 5-aza-dC. *FGF5* expression was induced by the 5-aza-dC treatment in a dose-dependent manner. Error bars mean SD (n = 3). (**C**) ESCC cell lines without *FGF5* methylation (KYSE-270, -410 and -450) and with methylation (KYSE-170 and -180) were treated with CDDP. In KYSE-270, -410 and -450 cells, *FGF5* expression was induced by CDDP in dose- and time-dependent manners. In contrast, in KYSE-170 and -180 cells, *FGF5* expression was not induced. Error bars mean SD (n = 3). (**D**) Overexpression of *FGF5* in an ESCC cell line with methylation (KYSE-180) by transient transfection of pBApo-CMV empty or *FGF5*. Error bars mean SD (n = 3). (**E**) KYSE-180 cells transfected with pBApo-CMV empty and *FGF5* were treated with CDDP. When transfected with *FGF5*, the cells revealed survival advantage after CDDP treatment. Error bars mean SD (n = 3).

We further investigated the mechanisms of how *FGF5* methylation was involved in the sensitivity of dCRT. First, we analyzed the influence of *FGF5* methylation in its promoter CpG island on its expression. Since *FGF5* had two splice variants^25^, we designed primers to distinguish the two variants (Supplementary Fig. S4A and Table S4). Among the 12 ESCC cell lines, three cell lines (KYSE 30, 170, and 180) had high levels of methylation, which was confirmed by deep bisulfite sequencing (Supplementary Fig. S4B). The three cell lines with high methylation did not have *FGF5* expression while two of nine cell lines with low methylation had high expression (Fig. 4A). Also, treatment of KYSE-170 and KYSE-180 cells that had high methylation levels with a DNA demethylating agent, 5-aza-2′-deoxycytidine (5-aza-dC), induced *FGF5* expression, especially its long variant, in a dose-dependent manner (Fig. 4B). These showed that *FGF5* promoter methylation repressed its expression, as is observed for methylation of promoter CpG islands of many genes^26^.

Then, influence of dCRT on *FGF5* expression was analyzed by treating KYSE-270, 410, and 450 cells, which had low *FGF5* methylation but low expression, with CDDP. As expected, *FGF5* expression was induced after CDDP treatment in a time- and dose-dependent manner (Fig. 4C). In contrast, in KYSE-170 and 180 cells with high *FGF5* methylation, *FGF5* expression was not induced, even after CDDP treatment (Fig. 4C). These results showed that *FGF5* expression can be induced by CDDP treatment in ESCCs with unmethylated *FGF5*. Further, the impact of *FGF5* expression on sensitivity to CDDP was analyzed by expressing *FGF5* in KYSE-180 cells with *FGF5* promoter hypermethylation (Fig. 4D). When treated with CDDP, *FGF5*-expressing KYSE-180 cells showed resistance to CDDP treatment (Fig. 4E). This suggested that *FGF5* is induced by dCRT and confers resistance, but that its methylation disables the induction and confers sensitivity.

### FGF5 expression and methylation in normal esophageal mucosae

**Figure 5:**
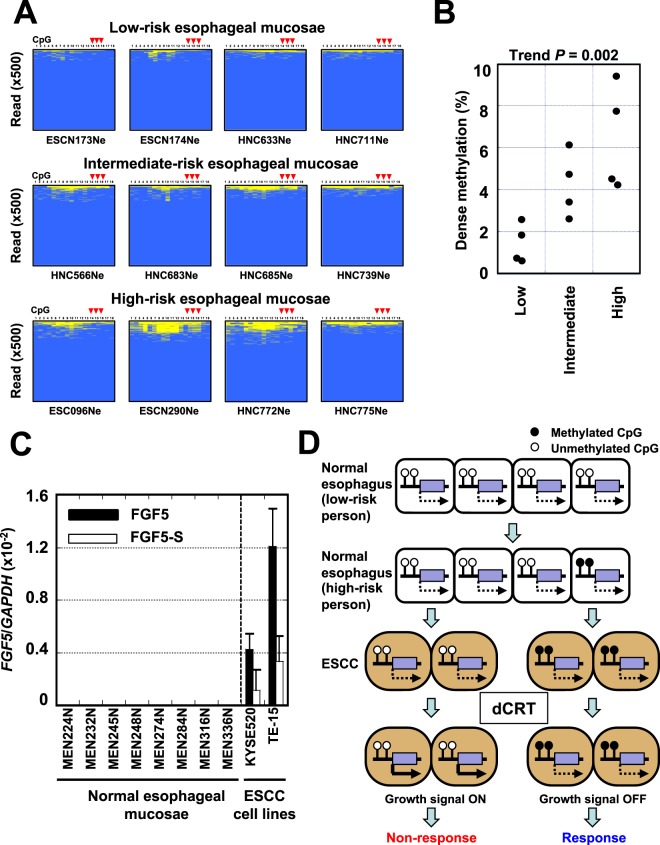
FGF5 methylation levels in normal esophageal tissues with different risk levels. (**A**) Deep bisulfite sequencing of normal esophageal mucosae from healthy people without exposure to lifestyle risk factors (low risk), normal esophageal mucosae from healthy people with exposure (intermediate risk), and non-cancerous esophageal mucosae of cancer patients, all of whom had exposure (high risk). The fraction of methylated DNA molecules increased according to the risk level. The position of the original consecutive CpG sites (cg10031614, cg12528713, and cg20528583) are marked by red arrowheads. (**B**) Fraction of densely methylated DNA molecules in normal esophageal tissues in the three risk groups. When 9 or more CpG sites were methylated among the 18 CpG site in a molecule, the molecule was counted as a densely methylated DNA molecule. The fraction of densely methylated DNA molecules significantly increased according to the increased risk level (Jonckheere-Terpstra trend test). (**C**) Expression levels of *FGF5* and *FGF5-S* variants in non-cancerous esophageal surgical samples and two ESCC cell lines. Error bars mean SD (n = 3). (**D**) Model on the origin of *FGF5* methylation and its role in the sensitivity to dCRT. After exposure to risk factors for ESCCs, aberrant methylation of multiple genes, including *FGF5*, creeps into esophageal mucosa. When an ESCC develops from a cell without *FGF5* methylation, it is capable of expressing *FGF5* upon dCRT and thus is resistant. When an ESCC happens to develop from a cell with *FGF5* methylation, the ESCC cannot express *FGF5* upon dCRT and becomes vulnerable to dCRT.

To explore the origin of the *FGF5* methylation, its presence was analyzed in normal esophageal mucosae of individuals with different risk levels of ESCC. Normal mucosae of low-risk individuals (healthy people without past exposure to lifestyle risk factors) had *FGF5* methylation levels of 0.6–2.6%, those of intermediate-risk individuals (healthy people with the lifestyle risk factors) had levels of 2.6–6.1%, and those of high-risk individuals (ESCC patients with past exposure to lifestyle risk factors) had methylation levels of 4.2–9.4% (Fig. 5A,B).

The *FGF5* methylation in normal esophageal mucosae, being 9.4% at the highest, suggested that the *FGF5* methylation was the consequence of its low expression in normal esophageal mucosae. It is well established that genes with low expression tend to be methylated^27,28^. Expression analysis confirmed that *FGF5* expression was very low in non-cancerous esophageal mucosa samples irrespective of their methylation levels (Fig. 5C).

## Discussion

In the present study, we discovered that *FGF5* methylation is a sensitivity marker of ESCC to dCRT. Importantly, the initial finding by a genome-wide screening was validated and re-validated using independent sample sets. The specificity and sensitivity in the combined validation and re-validation sets were 45% and 90%, respectively. The cut-off value established in the screening set was maintained, and the values were unlikely to suffer from overfitting. Clinically, we aim to use the marker to reduce the number of patients who have to undergo high-risk salvage surgery due to poor response to dCRT. Therefore, patients who are predicted to be sensitive to dCRT by *FGF5* methylation should respond to dCRT with a high probability. To eliminate false positives, we adopted a conservative cut-off value, and achieved a high specificity of 90% avoiding the issue of overfitting. Therefore, *FGF5* methylation is a promising sensitivity marker for ESCC to dCRT. To advance this finding, we need further validation using samples from different hospitals and a prospective cohort study.

*FGF5* methylation was in its promoter CpG island, and, when methylation was present, it consistently repressed *FGF5* expression. Some ESCC cell lines without *FGF5* methylation did not express *FGF5*, but treatment of the cells with CDDP induced *FGF5* expression. FGF5 is an oncogenic growth factor^29^, but no expression was reported in 13 ESCC cell lines in stable culture^30^. In glioblastoma, *FGF5* overexpression in primary samples and its growth-promoting effect have been reported^31^. In breast cancers, *FGF5* overexpression and its influence on poor survival have been reported^32^. Our data indicated that the *FGF5* is induced by dCRT in ESCC cells without its methylation and that it supports survival of the ESCC cells, leading to clinical resistance. If *FGF5* is methylated, *FGF5* cannot be induced, and this will lead to cell death and thus clinical response. Such difference between “unexpressed but ready to be expressed” and “unexpressed and cannot be expressed” is well known for *MGMT* in glioma^33,34^. The importance of *FGF5* expression for resistance to dCRT suggests that inhibition of *FGF5* or its pathway may have therapeutic benefit in increasing the response to dCRT of ESCC, especially when *FGF5* is not methylated.

Even in normal esophageal tissues, *FGF5* methylation was present (Fig. 5A,B). Therefore, if an ESCC develops from an esophageal cell with *FGF5* methylation, the ESCC is expected to be sensitive to dCRT (Fig. 5D). In contrast, if an ESCC develops from an esophageal cell without *FGF5* methylation, the ESCC is expected to be resistant (Fig. 5D). In general, it is reported that a gene tends to be methylated when it is not expressed^27,28,35^, and accumulation of aberrant DNA methylation of various genes, including drivers and passengers, leads to predisposition to cancer^36–39^. Taken together, it was considered that the lack of *FGF5* expression in normal esophageal mucosa facilitated *FGF5* methylation to creep into some esophageal cells, and that, when an ESCC happened to develop from such a cell with *FGF5* methylation, the ESCC paradoxically showed sensitivity to dCRT.

Since *FGF5* is not expressed without its induction, it is expected to be impossible to identify the difference in *FGF5* expression levels between responders and non-responders in biopsy specimens before treatment. In contrast, the methylome screening here was able to identify the difference between responders and non-responders.

## Conclusions

We identified that *FGF5* methylation was associated with the sensitivity of ESCC to dCRT.

## Supplementary information

Supplementary Information

## Data Availability

The data that support the findings of this study are available from the corresponding author upon reasonable request.
