# Supplementary material for: FGF5 methylation is a sensitivity marker of esophageal squamous cell carcinoma to definitive chemoradiotherapy
Source: Sci Rep. 2019 Sep 16;9:13347. doi: 10.1038/s41598-019-50005-6 (PMC6746740; doi:10.1038/s41598-019-50005-6)
Supplement: Supplementary file 1 — Supplementary Information [file 41598_2019_50005_MOESM1_ESM.pdf]

FGF5 methylation is a sensitivity marker of esophageal squamous cell carcinoma to definitive chemoradiotherapy

Supplementary Information

Authors

Jun Iwabu, Satoshi Yamashita, Hideyuki Takeshima, Takayoshi Kishino, Takamasa Takahashi, Ichiro Oda, Kazuo Koyanagi, Hiroyasu Igaki, Yuji Tachimori, Hiroyuki Daiko, Hidetsugu Nakazato, Kazuhiro Nishiyama, Yi-Chia Lee, Kazuhiro Hanazaki, and Toshikazu Ushijima

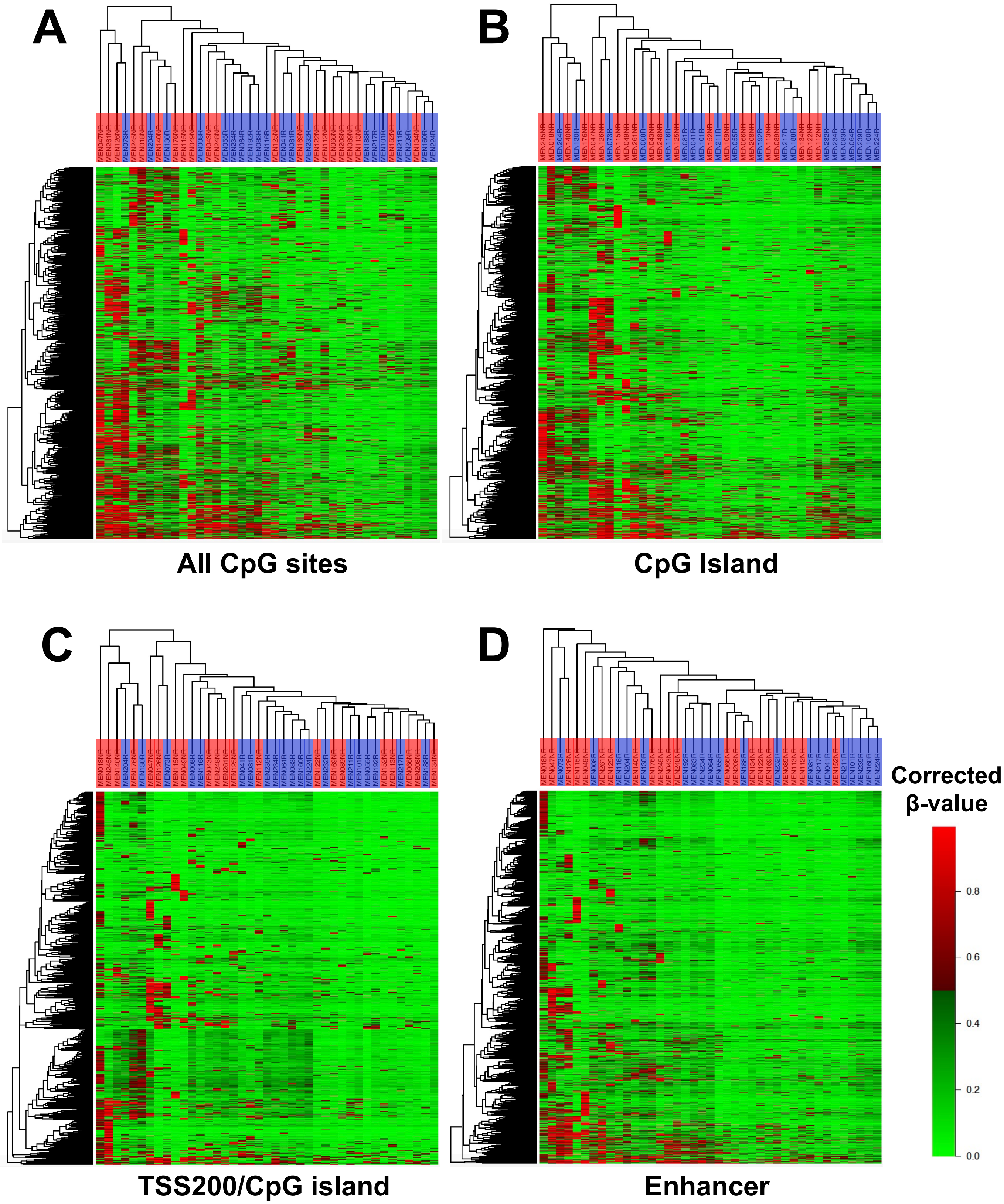

Supplementary Figure S1

Supplementary Figure S1. Unsupervised hierarchical clustering analysis of DNA methylation in the screening set.

Samples in blue and red show the responders and non-responders, respectively. (A) Analysis using 5,000 CpG sites randomly selected from all of the CpG sites on autosome that were unmethylated (β-value <0.2) in normal esophageal mucosae and peripheral leukocytes (126,963 probes). (B) Analysis using 5,000 CpG sites randomly selected from the probes in CpG islands (88,578 probes). (C) Analysis using 2,000 CpG sites randomly selected from the probes in TSS200/CpG island regions (23,253 probes). (D) Analysis using 5,000 CpG sites randomly selected from the probes in enhancers (12,057 probes).

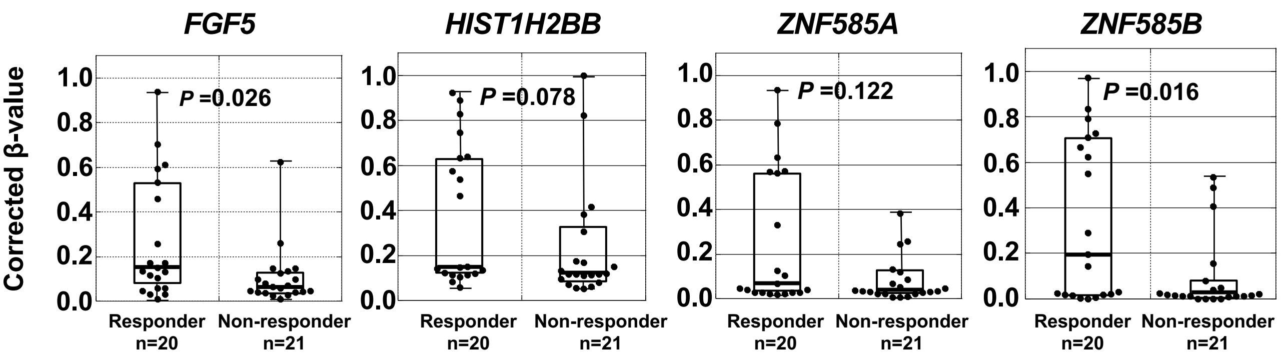

Supplementary Figure S2. β-values of the four candidate genes in the screening set.

The β-values obtained by the bead array analysis were corrected for cancer cell fractions, and are shown for responders and non-responders. Whiskers show maximum and minimum β-values.

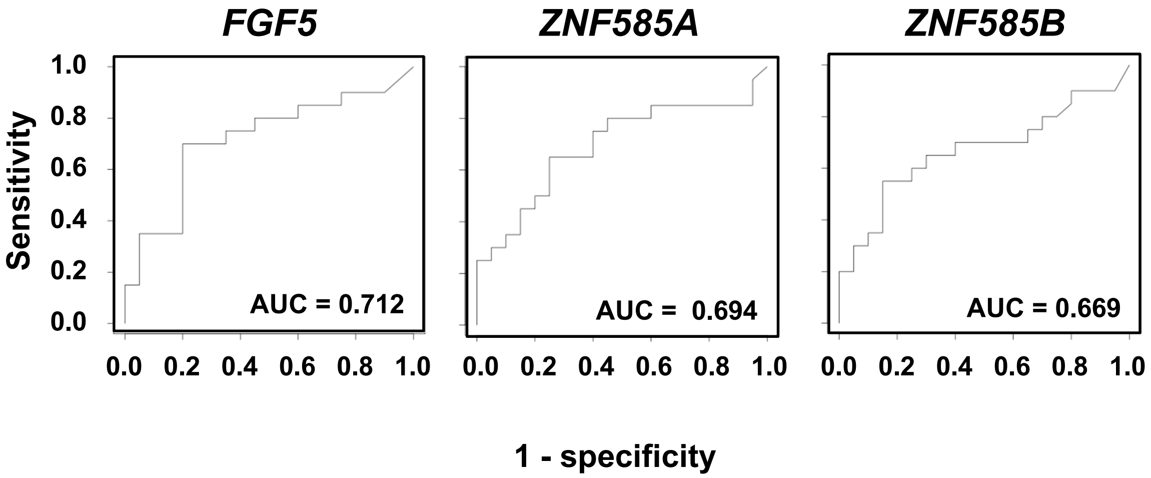

Supplementary Figure S3. ROC curves of the three candidate genes.

The ROC curves were drawn using the data from the screening set. The cut-off values of 0.10 (*FGF5*) and 0.21 (*ZNF585A*) were established based upon the maximum Youden index (sensitivity + specificity - 1).

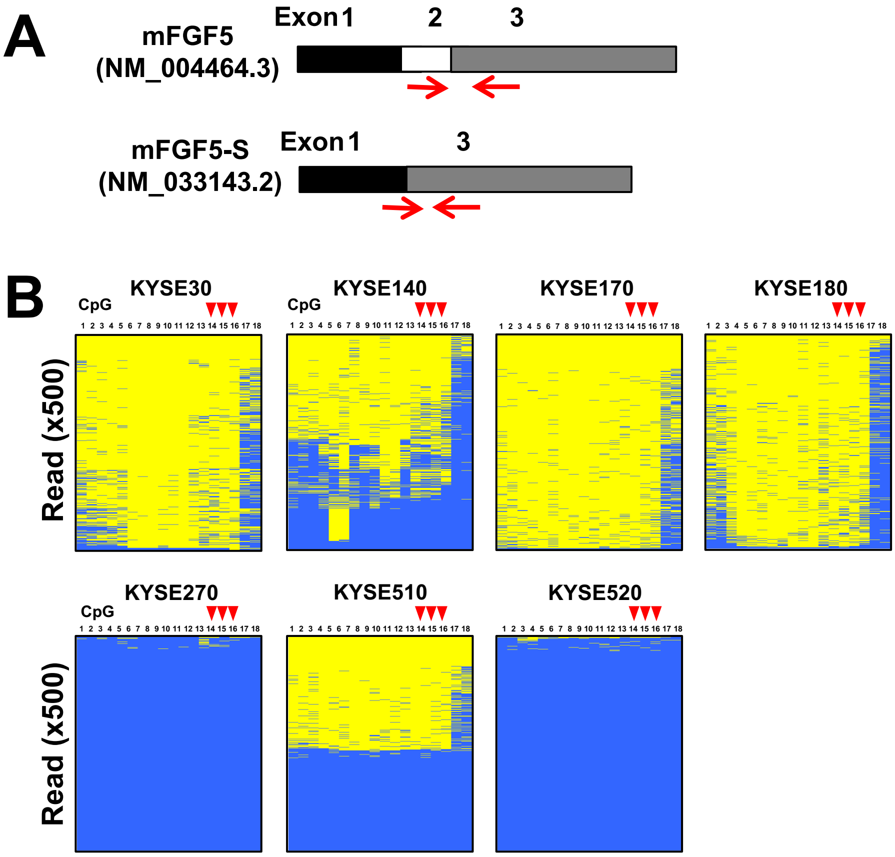

Supplementary Figure S4. Design of primers for FGF5 variants, and FGF5 methylation and expression in ESCC cell lines.

(A) Design of primers specific to *FGF5* (exons 1, 2, and 3) variant and for *FGF5-S* variant (exons 1 and 3). Primer locations are shown by red arrows. (B) Deep bisulfite sequencing of seven ESCC cell lines. The position of the original consecutive probes (cg10031614, cg12528713, and cg20528583) identified by the bead array analysis are marked by red arrowheads.

Supplementary Table S1. Clinicopathological features of the 117 samples in the screening, validation, and re-validation sets

Feature	Screening set			Validation set			Re-validation set		
	Responder	Non-responder	*P* value	Responder	Non-responder	*P* value	Responder	Non-responder	*P* value
Number of patients	20	21		21	13		26	16	
Age (years)[a]	65.0±6.2	64.3±9.8	0.928[b]	68.7±4.8	63.7±6.9	0.021[b]	66.2±7.9	65.3±10.5	0.816[b]
Gender									
Male	19	15	0.045[c]	19	9	0.114[c]	21	9	0.088[c]
Female	1	6		2	4		5	7	
Location									
Cervical	3	0	0.180[b]	3	1	0.859[b]	3	2	0.705[b]
Upper	4	5		3	3		3	3	
Middle	12	12		9	6		13	9	
Lower	1	4		6	3		7	2	
Radition dose (Gy)									
60	6	13	0.041[c]	9	5	0.800[c]	17	7	0.169[c]
50.4	14	8		12	8		9	9	
cT									
1	7	1	0.004[b]	5	0	0.173[b]	8	0	0.009[b]
2	3	0		1	0		2	1	
3	10	20		13	10		16	11	
4	0	0		2	3		0	4	
cN									
0	1	3	0.007[b]	1	1	0.127[b]	3	0	0.782[b]
1	16	6		12	6		14	1	
2	3	7		8	3		8	11	
3	0	5		0	3		1	4	
cM									
0	17	13	0.095[c]	16	9	0.655[c]	22	9	0.042[c]
1	3	8		5	4		4	7	
cStage									
1	0	0	0.079[b]	0	0	0.484[b]	0	0	0.041[b]
2	10	4		5	1		9	1	
3	7	9		11	8		13	8	
4	3	8		5	4		4	7	

[a]Mean±SD; [b]*P* values were calculated by two-sample t-test; [c]*P* values were calculated by the chi-square test

Supplementary Table S2. Backgrounds of all 117 samples

Sample No.	Group	Mutation Analysis					Methylation Analysis		Fraction	Age	Gender	Response	Location	T	N	M	cStage
		CP1 Panel		Repair Panel	SWI/SNF Panel		Infinium										
		Analyzed by Kishino et al.	This Study	This Study	Analyzed by Nakazato et al.	This Study	Analyzed by Takahashi et al.	This Study									
MEN008	Screening set	○		○	○		○		56.0	61	M	R	Lt	T3	N2	M0	IIIB
MEN018	Screening set	○		○	○			○	76.6	63	M	Non	Mt	T3	N2	M1	IV
MEN041	Screening set	○		○	○		○		78.4	65	M	R	Mt	T3	N1	M0	IIIA
MEN043	Screening set		○	○		○		○	48.6	42	F	Non	Ut	T3	N1	M0	IIIA
MEN047	Screening set		○	○		○		○	40.9	63	M	Non	Lt	T3	N3	M1	IV
MEN049	Screening set		○	○		○		○	66.7	59	M	Non	Mt	T3	N2	M1	IV
MEN055	Screening set	○		○	○		○		80.8	64	M	R	Ce	T3	N1	M0	IIIA
MEN064	Screening set	○		○	○			○	68.9	66	M	R	Ce	T2	N1	M0	IIB
MEN066	Screening set		○	○		○		○	61.5	47	F	Non	Mt	T3	N2	M0	IIIB
MEN073	Screening set	○		○	○		○		85.3	70	F	R	Mt	T3	N1	M0	IIIA
MEN081	Screening set	○		○	○			○	64.9	66	M	R	Mt	T1b	N2	M1	IV
MEN083	Screening set	○		○	○			○	50.2	75	M	R	Mt	T1b	N1	M0	IIB
MEN089	Screening set	○		○	○		○		87.2	62	F	Non	Mt	T3	N2	M0	IIIB
MEN101	Screening set	○		○	○		○		61.9	65	M	R	Mt	T3	N1	M1	IV
MEN112	Screening set	○		○	○		○		67.0	68	F	Non	Mt	T3	N2	M0	IIIB
MEN113	Screening set	○		○		○	○		65.6	69	M	Non	Ut	T3	N0	M0	IIA
MEN115	Screening set	○		○	○		○		59.6	67	F	Non	Mt	T1b	N1	M0	IIB
MEN116	Screening set	○		○	○		○		42.7	64	M	R	Mt	T2	N1	M0	IIB
MEN122	Screening set	○		○		○	○		44.9	69	M	Non	Ut	T3	N0	M0	IIA
MEN125	Screening set		○	○	○			○	25.2	58	M	Non	Mt	T3	N3	M0	IIIC
MEN126	Screening set	○		○	○		○		74.8	69	M	Non	Mt	T3	N2	M1	IV
MEN130	Screening set	○		○		○	○		54.0	74	M	R	Ut	T1b	N1	M0	IIB
MEN134	Screening set	○		○	○			○	80.9	79	M	Non	Lt	T3	N2	M0	IIIB
MEN140	Screening set	○		○		○	○		44.8	75	M	Non	Lt	T3	N3	M0	IIIC
MEN152	Screening set	○		○		○	○		64.2	55	M	Non	Mt	T3	N3	M1	IV
MEN160	Screening set	○		○	○			○	59.2	64	M	R	Mt	T2	N1	M0	IIB
MEN169	Screening set	○		○	○			○	70.8	79	M	Non	Ut	T3	N1	M0	IIIA
MEN176	Screening set	○		○	○			○	40.0	54	M	Non	Lt	T3	N3	M1	IV
MEN188	Screening set	○		○	○			○	78.2	56	M	R	Mt	T3	N1	M1	IV
MEN192	Screening set	○		○		○		○	79.9	67	M	R	Mt	T1b	N1	M0	IIB
MEN204	Screening set	○		○	○			○	74.8	65	M	R	Mt	T1b	N1	M0	IIB
MEN208	Screening set	○		○	○			○	73.9	61	M	Non	Mt	T3	N1	M1	IV
MEN211	Screening set	○		○	○			○	78.4	55	M	R	Mt	T3	N2	M0	IIIB
MEN217	Screening set		○	○		○		○	40.0	62	M	R	Ut	T3	N1	M0	IIIA
MEN224	Screening set		○	○		○		○	52.0	73	M	R	Ce	T3	N1	M0	IIIA
MEN232	Screening set		○	○		○		○	58.0	53	M	R	Ut	T1b	N1	M0	IIB
MEN234	Screening set		○	○		○		○	58.0	59	M	R	Ut	T1b	N1	M0	IIB
MEN239	Screening set		○	○		○		○	44.0	75	M	R	Mt	T3	N0	M0	IIA
MEN245	Screening set		○	○		○		○	54.0	75	M	Non	Mt	T3	N0	M0	IIA
MEN248	Screening set		○	○		○		○	62.0	60	M	Non	Ut	T3	N1	M1	IV
MEN261	Screening set		○	○		○		○	44.0	77	F	Non	Mt	T3	N1	M0	IIIA
MEN120	Validation set								42.2	67	F	Non	Lt	T3	N1	M0	IIIA
MEN223	Validation set								35.5	71	M	R	Mt	T3	N2	M0	IIIB
MEN264	Validation set								81.1	70	M	R	Mt	T2	N2	M1	IV
MEN274	Validation set								58.1	60	M	Non	Ut	T3	N1	M0	IIIA
MEN284	Validation set								22.6	67	M	R	Ut	T3	N1	M1	IV
MEN302	Validation set								81.1	74	M	R	Mt	T3	N2	M0	IIIB
MEN316	Validation set								85.1	67	M	R	Ce	T3	N1	M0	IIIA
MEN336	Validation set								38.3	62	M	Non	Ut	T3	N1	M0	IIIA
MEN348	Validation set								31.6	55	M	R	Lt	T3	N2	M0	IIIB
MEN354	Validation set								77.9	66	M	R	Ce	T1b	N1	M0	IIB
MEN376	Validation set								64.9	71	M	Non	Mt	T4b	N2	M0	IIIC
MEN388	Validation set								44.9	64	M	Non	Mt	T3	N1	M0	IIIA
MEN391	Validation set								28.1	75	M	R	Mt	T3	N0	M0	IIA
MEN397	Validation set								83.0	73	M	R	Lt	T1b	N1	M0	IIB
MEN415	Validation set								60.8	73	M	R	Lt	T1b	N1	M0	IIB
MEN425	Validation set								83.0	68	F	Non	Mt	T4b	N2	M1	IV
MEN430	Validation set								63.0	63	M	R	Ut	T4b	N2	M1	IV
MEN431	Validation set								87.5	68	M	R	Lt	T1b	N1	M0	IIB
MEN433	Validation set								51.4	75	M	R	Ce	T4b	N1	M0	IIIC
MEN435	Validation set								89.8	67	M	R	Lt	T3	N2	M0	IIIB
MEN445	Validation set								88.7	59	M	Non	Ut	T3	N3	M0	IIIC
MEN449	Validation set								52.0	68	M	R	Mt	T3	N1	M0	IIIA
MEN451	Validation set								84.3	58	M	Non	Ce	T3	N0	M0	IIA
MEN452	Validation set								75.5	72	M	R	Mt	T1b	N1	M1	IV
MEN454	Validation set								64.1	64	M	R	Mt	T3	N2	M0	IIIB
MEN466	Validation set								51.3	74	M	R	Ut	T3	N1	M1	IV
MEN468	Validation set								49.7	51	F	Non	Mt	T3	N3	M1	IV
MEN471	Validation set								59.3	76	M	Non	Lt	T3	N1	M1	IV
MEN503	Validation set								25.6	55	F	Non	Mt	T3	N3	M0	IIIC
MEN504	Validation set								84.4	68	F	R	Mt	T3	N1	M0	IIIA
MEN511	Validation set								74.4	63	F	R	Mt	T3	N2	M0	IIIB
MEN519	Validation set								87.7	65	M	Non	Mt	T4b	N1	M0	IIIC
MEN527	Validation set								81.7	72	M	Non	Lt	T3	N2	M1	IV
MEN535	Validation set								71.9	70	M	R	Lt	T3	N1	M0	IIIA
MEN026	Re-validation set								82.1	72	F	Non	Lt	T3	N2	M1	IV
MEN061	Re-validation set								23.0	62	M	R	Mt	T3	N2	M0	IIIB
MEN143	Re-validation set								29.9	63	M	R	Lt	T3	N2	M0	IIIB
MEN207	Re-validation set								83.7	56	M	R	Lt	T1b	N1	M0	IIB
MEN243	Re-validation set								51.4	52	F	R	Mt	T3	N1	M0	IIIA
MEN247	Re-validation set								34.9	78	M	R	Mt	T1b	N1	M1	IV
MEN257	Re-validation set								100.0	60	M	R	Mt	T3	N2	M0	IIIB
MEN258	Re-validation set								79.0	66	M	R	Mt	T2	N1	M1	IV
MEN297	Re-validation set								49.0	66	M	R	Mt	T1b	N1	M0	IIB
MEN301	Re-validation set								61.6	78	M	R	Lt	T1b	N1	M0	IIB
MEN304	Re-validation set								82.9	63	M	R	Mt	T3	N1	M0	IIIA
MEN311	Re-validation set								49.4	75	M	R	Lt	T3	N1	M0	IIIA
MEN333	Re-validation set								35.0	77	M	Non	Ut	T3	N1	M1	IV
MEN334	Re-validation set								74.2	72	M	R	Mt	T3	N1	M0	IIIA
MEN367	Re-validation set								83.4	44	M	Non	Mt	T3	N3	M1	IV
MEN370	Re-validation set								61.5	70	M	R	Lt	T3	N0	M1	IV
MEN406	Re-validation set								62.3	45	F	Non	Mt	T4a	N1	M1	IV
MEN419	Re-validation set								67.1	66	F	Non	Ce	T3	N1	M0	IIIA
MEN422	Re-validation set								85.7	73	F	Non	Mt	T4b	N1	M0	IIIC
MEN440	Re-validation set								99.0	66	M	Non	Mt	T3	N2	M1	IV
MEN446	Re-validation set								71.6	54	F	R	Ut	T3	N0	M0	IIA
MEN447	Re-validation set								49.2	71	M	Non	Ut	T4b	N0	M0	IIIC
MEN456	Re-validation set								92.4	74	M	Non	Mt	T3	N2	M1	IV
MEN473	Re-validation set								88.4	70	M	Non	Ut	T3	N2	M0	IIIB
MEN486	Re-validation set								50.8	62	M	R	Mt	T3	N3	M1	IV
MEN488	Re-validation set								45.1	76	M	R	Mt	T3	N2	M0	IIIB
MEN497	Re-validation set								62.8	78	M	Non	Mt	T3	N2	M0	IIIB
MEN510	Re-validation set								74.0	67	M	R	Mt	T2	N2	M0	IIIA
MEN512	Re-validation set								34.6	50	M	Non	Mt	T3	N2	M1	IV
MEN528	Re-validation set								82.1	50	M	R	Ut	T3	N2	M0	IIIB
MEN533	Re-validation set								77.8	68	M	R	Ce	T3	N2	M0	IIIB
MEN543	Re-validation set								24.6	59	M	R	Ce	T3	N1	M0	IIIA
MEN545	Re-validation set								81.1	68	M	R	Lt	T1b	N1	M0	IIB
MEN546	Re-validation set								62.5	78	F	R	Lt	T3	N2	M0	IIIB
MEN550	Re-validation set								86.5	74	M	R	Mt	T1b	N1	M0	IIB
MEN557	Re-validation set								79.8	61	M	Non	Ce	T3	N1	M0	IIIA
MEN560	Re-validation set								93.4	74	F	Non	Mt	T3	N2	M0	IIIB
MEN568	Re-validation set								60.8	39	F	R	Mt	T1b	N1	M0	IIB
MEN570	Re-validation set								60.3	67	M	R	Ce	T3	N0	M0	IIA
MEN571	Re-validation set								76.4	66	F	R	Ut	T1b	N1	M0	IIB
MEN589	Re-validation set								69.7	63	F	Non	Lt	T4a	N1	M0	IIIC
MEN599	Re-validation set								44.6	60	F	Non	Mt	T2	N1	M0	IIB

Supplementary Table S3. Customized primers for mutation analysis of repair genes ("Repair Panel")

Amplicon ID	Gene	Forward primer sequence	Reverse primer sequence	Chr	Amplicon start	Amplicon end
AMPL7152997103	*ATM*	TTTTTACAGACAGTGATGTGTGTTCTG	CCGATCTAGATGTTTAATTGTTTCAGGATC	chr11	108098312	108098565
AMPL7152997104	*ATM*	TGAGAAATTTAAGCGCCTGATTCGA	ATGCAAGGCATAATGATATATAGGAAGCAA	chr11	108098510	108098742
AMPL7152997108	*ATM*	TCAACGAGTTTCTGAAATTGCATTTTGT	GCGACAGTAATCTGTTAAGCCATTTATTTA	chr11	108099866	108100100
AMPL7152997081	*ATM*	GTTGCCATTCCAAGTGTCTTATTTTTGT	CATGAGGAAAAATTCACAAACAACAACC	chr11	108106328	108106601
AMPL7152996697	*ATM*	GCAGTTTTAAAATCCTTTTTCTGTATGGGA	CTCTATGAACATCTTGTGAAGGTTTCAGA	chr11	108114563	108114736
AMPL7152997278	*ATM*	TGTTCTCTGTGTACTTCAGGCTCTA	CATGGTCTTGCAAGATCAAAAGTATTTCAA	chr11	108114682	108114906
AMPL7152997084	*ATM*	AGCATACCACTTCATAACTGTTCAGT	TCGAAAGTTGACAGCCAAAGTCT	chr11	108115433	108115596
AMPL7152997085	*ATM*	ATCTTAGCAGCTCTTACTATCTTCCTCA	ACTTAGACTTCTATGTTTGAATGAAGAAGC	chr11	108115545	108115814
AMPL7152997416	*ATM*	CCAGGTGTCTTCTAACGCTGAT	TGACAGATATCTGCCATCAATTCAATCAAA	chr11	108117579	108117850
AMPL7152996749	*ATM*	GGATTTCGTAATATTGCCGTCAAAGAAAA	AGAACACAGGTTTTAAAAGCCCAAAATG	chr11	108117791	108117943
AMPL7152997105	*ATM*	GATACGAGATCGTGCTGTTCCA	GACACTGTAATCACTAGATTCTCTTTGTGT	chr11	108119558	108119740
AMPL7152997106	*ATM*	TCCTTGGAGATTTCTCAATCTTACACTACT	GAAAGGATTCCACTGAAAGTTTTCTGAAAA	chr11	108119680	108119912
AMPL7152997070	*ATM*	ATTTCCTTTTAGTTTGTTAATGTGATGGAATAG	GGTTTGACCTCTTGTCTTGACACA	chr11	108121363	108121606
AMPL7152997071	*ATM*	CGATGCCTTACGGAAGTTGCAT	TGTAAGTCAGACATAATGCATGCTGAA	chr11	108121560	108121831
AMPL7152997141	*ATM*	CCCTCCAATAGCTTGCTTTTCAC	AAAAACAGCAGCATGCTAATGAACT	chr11	108122517	108122791
AMPL7152997235	*ATM*	AGTATTCTTTACATGGCTTTTGGTCTTCT	CAGCTACTACCCAGCTAAAATTATCATCTT	chr11	108123486	108123721
AMPL7152997181	*ATM*	TACATATAAGGCAAAGCATTAGGTACTTGG	TTTTCTATACCACATTCTCTCACAATGGTT	chr11	108124479	108124660
AMPL7152997187	*ATM*	CAGACAACTTTTGACAAGATGGACTTTTT	CCTTCCTAACAGTTTACCAAAGTTGAATCA	chr11	108124601	108124831
AMPL7152997077	*ATM*	ACAGTGATCTCCTAGTTGTTTTTAGAGC	TCCTCTTCAGCTATTACACCCATGTAA	chr11	108126774	108127033
AMPL7152997079	*ATM*	GGTGTCCTTGGCTGCTACTG	GTCTTCCAAACAAATGTAATAATTTTCACAGGA	chr11	108126986	108127151
AMPL7152997386	*ATM*	GTTTTGCTTATACTGTATGACTACGTGGAA	CTCCTGCACATTGCATTAGAGACTAT	chr11	108128035	108128229
AMPL7152997390	*ATM*	CTGGAATTTGCATTTTTCCTTCTATTCACA	ACAATGAGTTGTGACAATCCCACT	chr11	108128173	108128425
AMPL7152997292	*ATM*	AAGATAGAGAAAACACTGTCTGCCAA	AGCTATATGTTGTGAGATGCATCCTTATTT	chr11	108129632	108129905
AMPL7152997102	*ATM*	CCATCTTGAACATCTTTGTTTCTCTTCC	GAGGCCTCTTATACTGCCAAATCAATATAA	chr11	108137863	108138138
AMPL7152997064	*ATM*	TTTTTGTGAAGAGGAGGAAATTTGAGTT	ACAAGAACTTGAGCATGTCTAAGAAAAGTA	chr11	108139045	108139211
AMPL7152997066	*ATM*	TAGCTGAAGAATATCTGTCAAAGCAAGATC	ACATCAGATAAAATCCAAGAGCTTCTTCAT	chr11	108139151	108139380
AMPL7152997118	*ATM*	ACTTTTTAAAGTAAATGATTTGTGGATAAACCT	GCTTAACAGAACACATCAGTTATTTTATAACTT	chr11	108141731	108141941
AMPL7152997097	*ATM*	TCCCTCCTACCATCTTAGTATCTAATGC	AAACATCTTGGTCACGACGATACAA	chr11	108141772	108142015
AMPL7152997098	*ATM*	TTTTTACCACAGCAATGTGTGTTCT	CAGATAGCAAAAGAAAAATAGGACCACAAA	chr11	108141965	108142178
AMPL7152997100	*ATM*	CCTTTCAGTGAGTTTTCTGAGTGCT	CGGGAAAAGAACTGTGGTTAAATATGAAAT	chr11	108143187	108143445
AMPL7152997089	*ATM*	CTTTGGAAAACTTACTTGATTTCAGGCA	CCATTACATTAAGAATGGCCCATTTTGAAT	chr11	108143233	108143488
AMPL7152997332	*ATM*	GAATATGCTTTGGAAAGTAGGGTTTGAA	ACCAAACTTGGTGAAGTAATTTATGGG	chr11	108150138	108150413
AMPL7152997199	*ATM*	CCAAAAGACAGAACTAGGATTAGTGAGT	CAGATAGGGCTACAGGATAAAACCA	chr11	108151575	108151822
AMPL7152997203	*ATM*	GAAAATCTGTTTTACTGACGTTGATAGCTG	CACAGTGACCTAAGGAAGCTTCTAATAAAA	chr11	108151767	108151942
AMPL7152997283	*ATM*	TCCCATCTCATAGATGAGGAAATCAAGA	AGATTTAGCCATTCCAAAACCAGATAATCT	chr11	108153298	108153531
AMPL7152997286	*ATM*	ACGTTTAGAAGACTTTATGGCATCTCATTT	GTATGGGTATGGTATGTGTGTTGCT	chr11	108153471	108153706
AMPL7152997095	*ATM*	GATCTTGTTATAAGGTTTTGATTCCACATCT	CCCTCATAGGCAAAATAAGGAAGAATATTTACA	chr11	108154952	108155112
AMPL7152997096	*ATM*	TTCTAACAGACTGCTTTCCAAAGATTCT	ATCTCATATCATTCAGGGAATGAAAAGTAC	chr11	108155051	108155249
AMPL7152997094	*ATM*	GGTAATATATGCCTTTTGAGCTGTCTTGA	CATTGAAGGTGTCAACCAATAAACTTCTT	chr11	108158247	108158521
AMPL7152997217	*ATM*	TTTATATATCTGGACTGTGATATGTCATTTGTG	GGTTTTATGACAATTGCTGATATAGGCAAATG	chr11	108159523	108159791
AMPL7152997223	*ATM*	ATTTTCCATCGCATGTGATTAAAGCAA	ATTTCAATTAATGCTGACAAGTAAAATACCATT	chr11	108159732	108160007
AMPL7152996658	*ATM*	AAAGTGTATTTATTGTAGCCGAGTATCTAATT	ACAGGTGATATATTTTAAGAATTCTGTGCTTCT	chr11	108160262	108160425
AMPL7152997112	*ATM*	AGCAAGCAGCTGAAACAAATAATGTTTAT	AGGAAGAACAGGATAGAAAGACTGCTTATA	chr11	108160362	108160582
AMPL7152997345	*ATM*	GTCAAGGCATATAAGAATTAGAGATGCTGA	GGGAGAAGCTACGTAATGACACATC	chr11	108163230	108163386
AMPL7152997349	*ATM*	TCTCCCTATATTAGGCCTTCTTGTATCATG	ACCAAATAAACATAAAACACTCAAATCCTTCTA	chr11	108163331	108163605
AMPL7152997395	*ATM*	ACAGAAACTAAAAGCTGGGTATCTTAGAC	GCTGAGTAATACGCAAATCCTTAAAAACA	chr11	108163888	108164163
AMPL7152997400	*ATM*	GCTTTTAGATCCTTTTCCTGACCATGT	GCCTGGCCTACGTATATATTTTTAATCACA	chr11	108164107	108164338
AMPL7152997083	*ATM*	GTTTTATTTCACAGGCTTAACCAATACGT	AAGAGCAAGACTTTGCAAAATATTCTTTCA	chr11	108165594	108165860
AMPL7152997241	*ATM*	AAACAAAAGTGTTGTCTTCATGCTAGTT	CTATATGTGATCCGCAGTTGACTGAA	chr11	108167956	108168164
AMPL7152997150	*ATM*	CGCACTCGGCCTTAAGGTTAAT	AAGGTCCACTGAAGTTCTTTATCTTCAAAT	chr11	108170312	108170570
AMPL7152997154	*ATM*	GCATCTTATACCAAGGCCCTTAAGTT	TGTGAAGTATCATTCTCCATGAATGTCAT	chr11	108170514	108170677
AMPL7152997404	*ATM*	TTCAGTGGAGGTTAACATTCATCAAGATT	CAGCAAACAGAACTGTTTTAGATATGCT	chr11	108172302	108172577
AMPL7152997356	*ATM*	CTTAAAAGGTAAACATTGCCTCCAGATT	CCTTCAAAAGGGTTTTCTTTGTCAAATC	chr11	108173436	108173623
AMPL7152997360	*ATM*	CCCTTTCTTTCTAGTTTTTAGAAGTACCCA	CACAGCACTCTTTAGATAAACAGGTCATAA	chr11	108173565	108173821
AMPL7152997082	*ATM*	TACTCAAACTATTGGGTGGATTTGTTTGT	TCCATCTTTCTCTAGAACTGAGTTTACAGT	chr11	108175363	108175636
AMPL7152997091	*ATM*	AGTGGGAGACAGACACATAAACAAG	GCAACACCTTCACCTAAAATAATCATGTT	chr11	108178537	108178810
AMPL7152997316	*ATM*	GGGAAATGTGGTTTTTGGGAATTTG	AAATCCAGCCAGAAAGCATCATTAAAAAT	chr11	108180754	108180931
AMPL7152997319	*ATM*	TCATTTTTCTTTAGACCTTCTTCAGGAACA	CCCTTATTGAGACAATGCCAACATTAATTT	chr11	108180872	108181101
AMPL7152997270	*ATM*	TGTATTCAGGAGCTTCCAAATAGTATGTTC	ATGGCATCTGTACAGTGTCTATAACAAAAT	chr11	108183076	108183309
AMPL7152997068	*ATM*	TTCCTTCTTCAATTTTTGTTGTTTCCATGT	ACACACACATAACTCCTTCATAAACAGAAT	chr11	108186513	108186701
AMPL7152997073	*ATM*	ATTCTGTTTATGAAGGAGTTATGTGTGTGT	ACATACTGAAATAACCTCAGCACTACAC	chr11	108186671	108186904
AMPL7152997125	*ATM*	ACACCCAGCTGATATTTTGGGATTTTA	GGAAAGAATATGGCAGAGTCCCAAA	chr11	108187955	108188135
AMPL7152997129	*ATM*	CTCCTGTTTAGGCCTTGCAGAA	TTGTTTAGAATGAGGAGAGAGGCAAAAA	chr11	108188088	108188302
AMPL7152997074	*ATM*	TTGTCCTTTGGTGAAGCTATTTATACATGT	GTTTCTCAAGTTTTCAGAAAAGAAGCCA	chr11	108190597	108190858
AMPL7152997092	*ATM*	AAGCCTATGATGAGAACTCTTTAACAACAA	ACTATTGGTAACAGAAAAGCTGCACT	chr11	108191929	108192204
AMPL7152996601	*ATM*	TTTTTCATTTCTCTTGCTTACATGAACTCT	GCCATGATAGGCTCCTGAAAACTA	chr11	108195990	108196138
AMPL7152997075	*ATM*	CCAGCTTCTCAAGGACAGTGATTT	GGAAAGTCAAGAGGTAAGATGACATAGTTT	chr11	108196090	108196315
AMPL7152997306	*ATM*	TCATGCAGACAGAGAGGTCCTT	TCCACAGCTAACTGAATTGTACTGTTTAAT	chr11	108196636	108196838
AMPL7152997308	*ATM*	GCTCCCTGAAAGGGCAATATTTCAA	TTTTTAAATAACAGTAAAACACTAATCCAGCCA	chr11	108196783	108197000
AMPL7152997080	*ATM*	GTACATGAAGGGCAGTTGGGTA	CTTGATGAAAAGATGAAGCATATTCATGCT	chr11	108198301	108198551
AMPL7152997334	*ATM*	AAATTGGTTGTGTTTTCTTGAAGGCA	TTTGGCTCTTTTCAGGAGAGCTT	chr11	108199724	108199921
AMPL7152997335	*ATM*	CATGAAATCATCGGAATTTGAAAACAAGC	CACTCACTCAGTTAACTGGTGAACATA	chr11	108199869	108200020
AMPL7152997252	*ATM*	TCCCTGGGATAAAAACCCAACTT	AATTTTCAACTGCTTTACATAAGAAGCGTT	chr11	108200828	108201041
AMPL7152997257	*ATM*	CGTGCACTGAAAGAGGATCGTA	AGGGTAGAATATTGGGCTGAGTAACA	chr11	108200989	108201179
AMPL7152997337	*ATM*	AGTACCCATTAGAAAGACCTTCAGATAAGA	ATCCTAGGCCTCCCATCATCTT	chr11	108201989	108202264
AMPL7152997340	*ATM*	TATGTACCAATTGGCTGCTAGAATGG	GCCTATGAGGAATTAGAGAGAGAAAATGAG	chr11	108202211	108202456
AMPL7152997296	*ATM*	CCTCATAGGCCTCTGCCTTTTT	CAGAAATTCATCTCTGTTTGCATTTGCT	chr11	108202446	108202692
AMPL7152997300	*ATM*	CCATCACACTTTGTTTATTATACTGGCCTT	GACTGAATATCACACTTCTAAAAGGTACGT	chr11	108202634	108202811
AMPL7152997093	*ATM*	AACTCTGAGAAGTTTAAATGTTGGGTAGTT	TCTCTACAGAGAGTAACACAGCAAGA	chr11	108203425	108203684
AMPL7152997069	*ATM*	CAGTGGTATCTGCTGACTATTCCTG	TTGCCAATATTTAACCAATTTTGACCTACA	chr11	108204528	108204803
AMPL7152997110	*ATM*	CGGCCTAAAGTTGTAGTTCTTAACCA	CTGCTTTAAATGACTGTATAGTCACCAGAT	chr11	108205551	108205750
AMPL7152996657	*ATM*	TGGACCACACAGGAGAATATGGAA	CTAAGGGCTAAGCCAGAGAAGGGAA	chr11	108205696	108205868
AMPL7152997366	*ATM*	TGGTTTGAGTGCCCTTTGCTAT	ACTTCACCCAACCAAATGGCAT	chr11	108206479	108206754
AMPL7152997113	*ATM*	GATTGGTTTCCTCCAAGGAGCTT	GCCTGTATCTTTTATGAGCACCATCTT	chr11	108213792	108214057
AMPL7152996663	*ATM*	CCCATTGGTGAATTTCTTGTTAACAATG	CAGCTGTCAGCTTTAATAAGCCATTAAAT	chr11	108214002	108214164
AMPL7152997087	*ATM*	TGCTTCCCTGTCCAGACTGTTA	GAAGTAACGGAAAACTGGTTGAAAATTTTG	chr11	108216316	108216553
AMPL7152997088	*ATM*	GAGAAATATGAAGTCTTCATGGATGTTTGC	CCAAACAACAAAGTGCTCAATCTACTATAT	chr11	108216493	108216680
AMPL7152997072	*ATM*	ACCAAGTCAGTGGTCTTAATTGAAATTATG	GTAATATCTTGGTAGGCAAACAACATTCC	chr11	108217914	108218174
AMPL7152997067	*ATM*	ACATGTGGTTTCTTGCCTTTGTAAAG	GCCCAGCCCATGTAATTTTGAC	chr11	108224395	108224668
AMPL7152997099	*ATM*	GCATAGGCTCAGCATACTACACA	CTATGTTAAAATGAAGCAGTGCTCTTCA	chr11	108225437	108225706
AMPL7152997376	*ATM*	TGACTGGCTTATTTGTATGATACTGGTTC	ATCTGAAAAACTGACAACAGGACCTT	chr11	108235713	108235988
AMPL7152997163	*ATM*	AAGGCCTTTAAACTGTTCACCTCA	TTCTAAAGGCTGAATGAAAGGGTAATTCA	chr11	108236000	108236275
AMPL7153194013	*ATR*	GATCAGAGAGAAATAACAGTTGCTGAGA	TAGAGTGACAGGACTGCCGTTAT	chr3	142168138	142168385
AMPL7153194022	*ATR*	GTATAAGGTAATGCACATGTCCTTCAATAG	CTTTTCCTGTATTTGCTCTTACAAAAGGG	chr3	142168332	142168583
AMPL7153128325	*ATR*	GATGAGAACTATAGCTGCATATCAAGTTCA	TCATCCTTCACTTAAATATTGATGTCAGCA	chr3	142171889	142172158
AMPL7153127531	*ATR*	AAAAACTATTTCAGTCAGTACAAAGTGGTT	GTCTTTTTCGAAGAGCATGTGAAGT	chr3	142176332	142176512
AMPL7153127549	*ATR*	ATCACGCATCAGCCTCATTGTA	TCTGGACATGAAGTTCTTTGAGTAAAGATT	chr3	142176465	142176675
AMPL7153194211	*ATR*	ACGCATTACTACATGATCCACTGA	CGTCATGGTGAAAATATTCTCTTTGATTCT	chr3	142177635	142177877
AMPL7153128769	*ATR*	TACATGTACGCATTCACCAGTCAA	GATCCTACATCATGGTAAGTTACCAAGAAT	chr3	142177823	142178082
AMPL7153193682	*ATR*	ATTCTTGGTAACTTACCATGATGTAGGATC	CTGATGTGATTAATGGATATCACTCTTCCT	chr3	142178052	142178260
AMPL7153193718	*ATR*	GGAAGGAAGGGATGGAAACACTT	TGAATGGGTGAACAACACTGCT	chr3	142180617	142180843
AMPL7153193726	*ATR*	AGTTTGGTCAGAATAGGTCTCAAACC	GCCTTCAATGACATTCTTTGGTTATGAAAT	chr3	142180795	142181046
AMPL7153193784	*ATR*	TCATTCCTGCTTTTTAAGAGTGATCTGT	CTACATCATGATGTGTAAGCCAAAAGATG	chr3	142183772	142184017
AMPL7153193792	*ATR*	TCCATTAGTCTACAATCCTTTCTCAGGT	ACATTTACACAGAAATTTTTGGCCCTAATT	chr3	142183960	142184136
AMPL7153118144	*ATR*	AAACTGAAGTTTACCATATCATCAAACCCT	CAACATTTAGTGAAATCCTCATTCCTCTAC	chr3	142185151	142185305
AMPL7153193748	*ATR*	TGATGGAAGTGTAGGTATCATGACTGATT	ACAGACTGCTGAACCTTTGTAATCAAAATA	chr3	142185246	142185479
AMPL7153193820	*ATR*	ACATAATTACCCAACATCAGTTTATAAGCA	ATCTTATCCCATGCGTGTGAACA	chr3	142186727	142186908
AMPL7153193827	*ATR*	GAATAGCTTTATTGAGGATTTCCTTGCATC	GCTCTTGATAGCATTGCCAAATAAATTTTC	chr3	142186855	142187066
AMPL7153118097	*ATR*	AGATCTTTTCATATAAAAAGGTAAAAGACCCT	ACAATTGATCTCTCGAATTTGTCATTCTCA	chr3	142188141	142188302
AMPL7153193649	*ATR*	TTCCATCAAGACAACAAAAACTTCATCG	AAAGCAAAATTTTTCTTCTGTGAAATGAAC	chr3	142188244	142188498
AMPL7153127789	*ATR*	GACTGTCCAGCCAAATCTGACT	GAAAACGGTATGTGGTAGTTTGAATCTTT	chr3	142188850	142189122
AMPL7153193811	*ATR*	ATCCTTACATATTTAATCTGCAAACATGCA	GTGGGATTTCCTTAGGTAGGTATCCTAT	chr3	142203937	142204166
AMPL7153128625	*ATR*	CTGGTTATTTTTATACATGTGCTTTGCCAT	GAGGGTAAGAACATGTTAATCCATGGT	chr3	142211879	142212078
AMPL7153128644	*ATR*	GCCCACTAGTAGCATAGCTCG	ACAAATGGATTTGTACAAAAACATAATGAACTA	chr3	142212030	142212253
AMPL7153128807	*ATR*	GATTAGTTATGTTCTCTCAGTTGGCCTT	GCCTACAATGCTCTCCTTAATGCAG	chr3	142215068	142215283
AMPL7153194233	*ATR*	TACAGTTCAGCGAGTCGTGATTC	TCTCTCACCAATGTGTTGTTACTTCC	chr3	142215233	142215393
AMPL7153194030	*ATR*	CCAGATAGTGTAGCCCTGATCATTAAA	GACTAGAAATGACCCAGAATTCCTACAG	chr3	142215714	142215936
AMPL7153194035	*ATR*	CCAGGATAGGCTCCTTGGCT	AGTTCTACCAAAGACAACTGTGTATTTTAAATT	chr3	142215888	142216158
AMPL7153193864	*ATR*	AATCCTGTCAGGTGACATTTATAGGC	AGCTACCGTGACATTTCTTGACTC	chr3	142217381	142217656
AMPL7153193959	*ATR*	CGCACCCATCCTAAAACTGCTT	ACCAATGAAAAATCTCTGAATGTCACCTTA	chr3	142218424	142218630
AMPL7153127887	*ATR*	TACCCAATTCACTAACTAAAAATTCTCCATTCA	CCCAAGCTATTATTGAAGTGATAATTGC	chr3	142222131	142222349
AMPL7153127902	*ATR*	AAAAACTAAGGTTTCCAGAGTTCCTATTCA	CAGAACCATCTCTAAAAGAACAGATCCTT	chr3	142223909	142224084
AMPL7153127918	*ATR*	GCAAGCCAAGGCTTTCATGTTC	AATTGCTGTTTCAATATAAACAGGTGGTTT	chr3	142224033	142224239
AMPL7153127724	*ATR*	GAACAACATAAACATAAAAGACTGGCCA	ATGATCTAGTATCTACTGTGGATTATGAAGACT	chr3	142226703	142226960
AMPL7153118337	*ATR*	GAACTGATAAAGGGAAGAGCTAATTGGT	CACAAATTTCAGGCACTGAAAGCT	chr3	142231026	142231174
AMPL7153128280	*ATR*	TTTGATTTGCTGTGTGGACATTTCTC	GCCTTAGAATGGTTAGCTTTAGATGTCATA	chr3	142231124	142231398
AMPL7153193846	*ATR*	CATTGGAGAAAGTAAGTTTCACATGTTCAA	AATATGTGAAAGTGTGTTACTGTCTACACA	chr3	142232286	142232524
AMPL7153127337	*ATR*	GCATTCAGATAGATGTTCTGGTTTCCT	TCAACTGAAGGAGTTGCTGTAATTTACTAA	chr3	142234166	142234440
AMPL7153193941	*ATR*	AATACATTAAATAAGCCTACATACCTGGTATTT	CTTCACTGTGGACTTACAAATGTAAAATATGT	chr3	142238486	142238666
AMPL7153128546	*ATR*	TGGTTAAGAGGTATGCTTCTGTACTCT	ATTCAGCTGCCTATGCCATTCA	chr3	142241404	142241592
AMPL7153128558	*ATR*	AGCCAGTAATGACTATATCCACTCTTACC	CATGGAAAAAGCAGTACACCTAAGAAAATT	chr3	142241541	142241758

AMPL7153194074	*ATR*	CCTCAATAGGACAGAGAACTCTTTTGTC	GGTGACAGTGCTTTTGAAAGGTTG	chr3	142242784	142242982
AMPL7153194081	*ATR*	CGAGCTTGAGAGTTTGCATCTTG	CTAGCTCAGGAAAACATACTAGTTTGTAGA	chr3	142242934	142243107
AMPL7153128306	*ATR*	CATTTTGTCATCTTTTCTTTAAGCCTCCAA	CAGGCCTTTGGAAATGGTTGTTAAA	chr3	142253848	142254115
AMPL7153127849	*ATR*	AAAACTTCCCTCCAAAAGTCATGGA	GATGCTGTGCAAGATTTTCTTCATGAAATA	chr3	142254768	142255042
AMPL7153127867	*ATR*	TCCTGGAGAACGGCTTTTATCTTTTT	CAGCTCAGTAATCAACTAGCACTTTTAATT	chr3	142254959	142255169
AMPL7153193769	*ATR*	ATGCCACAACTGAAATAGCATCATTT	CCTCTTATACACATCCAGCCTAAAGAAAC	chr3	142257147	142257388
AMPL7153127585	*ATR*	TATGAGGTAGTGGAAGATAGCTGCA	GATCTTTATTCCATGTTTAAAGCTGCCTTT	chr3	142257334	142257605
AMPL7153128375	*ATR*	GAATTCAAGTTAGTGCTACTGGAAAATGC	CCTTAGGGCTCATCTGCTTCTG	chr3	142259677	142259945
AMPL7153194165	*ATR*	TTTAGTCACTTTGGGTTGGGTGTT	GTGTTGGCATTGAAGATAAGAAAATGGT	chr3	142261285	142261532
AMPL7153128602	*ATR*	GCACTCTGGAACTATCACCACTT	GCTTTTGGAGAAACTTAATTAACCAAGTGT	chr3	142261481	142261751
AMPL7153128704	*ATR*	CAAATGGCTAGCAGCCAGAAAA	GTCAATACTTGCCTCATTTGCATCC	chr3	142266455	142266642
AMPL7153128721	*ATR*	GGCCCTGATATGGATCATCACT	GAGCTCCTGATGTACTAATAGCATGTTAAA	chr3	142266595	142266850
AMPL7153166506	*ATR*	AGGATAGGCTATAATTTACTCACCCTCTTT	TGGTCTGTTCTTGTTCCAAAGATGAATT	chr3	142268215	142268373
AMPL7153194192	*ATR*	CTTCAGATAATGAAGGGCACGTTCT	AGACAAATGTACTTTTTGGTAATTGTTGCA	chr3	142268320	142268556
AMPL7153193949	*ATR*	GGAATGAACAAAATACTGAATTGCAAACC	CTTTGTGTGAAAACTCACAATAAAATGGTT	chr3	142268917	142269184
AMPL7153127968	*ATR*	AACTCTTAAAAAGTACTGCTGTTGCCTA	CTCTGGAGCAGCATACACAGAAAT	chr3	142272014	142272165
AMPL7153127979	*ATR*	CTTTTAGCTGCAACCAGAGCTCTA	CCGTTTGTTTCTGGGATATTGGAGAT	chr3	142272117	142272341
AMPL7153127463	*ATR*	TAACAGCAAGCAAATAAAAATGGAGAATGG	ACATTTGAGTTTACGTCAACCTAGCT	chr3	142272410	142272646
AMPL7153193803	*ATR*	TTTTAGAGCTAGGTTGACGTAAACTCAAA	AAATTAGGTATTGTATACTCTTGTCCTTCAGC	chr3	142272614	142272888
AMPL7153194115	*ATR*	CTGATTTGAAAAGCAGAGCTTAAATAGGTT	GCCACTTCTCAACATGAATGTTCATC	chr3	142274657	142274821
AMPL7153128457	*ATR*	TGCAGACAGAAGCTTTTAGTTGAGAA	CAAGACTATTTAGACCACAAAATTAGGCAA	chr3	142274769	142275044
AMPL7153128480	*ATR*	CCCTGCATACATAGCCAGACA	TTTCAGCACCACAGGCACAAT	chr3	142275150	142275423
AMPL7153193909	*ATR*	CAAACCACACACACATTCTTGTGAG	CAGTTCATTTGAAGATCATATCCTGGAAGA	chr3	142277433	142277614
AMPL7153127951	*ATR*	GGAATAAATCCATGGAAGTGAGAGCAT	TCAGACCATCGTTTATAGATCTCTGTGTTA	chr3	142277547	142277765
AMPL7153128410	*ATR*	TCTGAGTATTCCAAACACGCACTT	TGCTTAAGAGCTGTAGAAGTTTGTTAGAAT	chr3	142277986	142278197
AMPL7153128427	*ATR*	CTCCAGGTCCAGTTTCTGAACAG	GTACTCACAATTTCTTTTGGCTTTACTACA	chr3	142278144	142278381
AMPL7153128785	*ATR*	CAGTAAATCCAAGTTCATTACAGGAAACC	AGAATTAAACATGTGGACATGAACCAAAAG	chr3	142279045	142279298
AMPL7153193693	*ATR*	ATTTTAAGCACTCCCTTGGCTACA	GGAAAACCTCAGCAGTAATAGTGATGG	chr3	142279945	142280183
AMPL7153127448	*ATR*	ACGACGCCTTTTGGGTGATATT	AACATCTAATACATTCTTGCTGCCTATGAA	chr3	142280134	142280349
AMPL7153128081	*ATR*	ACTAGTAACCTGAAAAATTACTTACCTCTGC	TGTGCCATTTACTGCAGTATTTCCTTA	chr3	142281048	142281207
AMPL7153128098	*ATR*	TCATACCCAGCTGGCACAAATT	ATGAAGAGCCATTATCAAAGCTGATAAAGA	chr3	142281158	142281372
AMPL7153128112	*ATR*	GCTTCTGCTTCAAAGGGAAATAGTG	AAGAACTCTTACTTTGGCAGATAGGTTG	chr3	142281317	142281570
AMPL7153128127	*ATR*	TTGGACTACCATACTCTAGCAGAACA	CTTTTTGAAGACTTGGTTTACCTCCATAGA	chr3	142281516	142281790
AMPL7153128141	*ATR*	ACAGCATGACCCATCACATTTCT	CAAATGAATTAGCACTTCGGATATACTTGT	chr3	142281737	142282012
AMPL7153128394	*ATR*	AAAAGGAGGATTTTATAGAACAGATGGCAA	AACAAAAGGACCATTGTTTAATTTGTCATG	chr3	142284906	142285162
AMPL7153193664	*ATR*	ACTCCATATCTAAAACTACATGGAGAAAATG	TGAAACTGAGTCACTTTAAGGATTAAAAGCT	chr3	142286801	142287044
AMPL7153194147	*ATR*	CCACGGAGCATCTCCACAAG	CGTTGGCGTGGTTGACTAGT	chr3	142297348	142297582
AMPL7153040994	*ATRX*	ATGATTTTAACAATCCATTAAGCTTTTAGTGC	CTTCTAACTACCAGCAGATTGATATGAGAG	chrX	76763792	76763965
AMPL7153178933	*ATRX*	CCAGCCACTGGCTGATACATTC	CATGCACTTGTAATGACTCCCATTTTT	chrX	76763913	76764143
AMPL7153178911	*ATRX*	ACATAACCTAGAAATAGTCAGACGTCAGA	ACCTTGGGAAATCCCGAATATCTTAAAAT	chrX	76776228	76776485
AMPL7153178896	*ATRX*	GGGTGGAGGGTACAAATTGGAG	GATTCCCTATACTTTAACACTATGCTGTCT	chrX	76776827	76777043
AMPL7153074327	*ATRX*	CCCTCAACAACAAGGCACTTGA	TCTAAACCAGTAGGTGATGTAGAATTTCAA	chrX	76777671	76777941
AMPL7153180916	*ATRX*	CCACTAAAGGTAGCTGAAACCAAC	CTGAGTATGAAGCAGAGAAGAAGGT	chrX	76778559	76778752
AMPL7153073554	*ATRX*	ACAAAAACAAAAACACATTTTGCATCAACT	CTAGTACAGTTGACCATAGAACTATGGAGA	chrX	76778697	76778966
AMPL7153073496	*ATRX*	TAGTAAAATTTCCTTAGTCCACGTTGGTT	GGTGGAGCGTCATTTTACTATGAATGA	chrX	76812863	76813039
AMPL7153073519	*ATRX*	CTGGCTCAAAAGTATAAAGTTCAGTAAGC	TGGCAATATACTACCATAAAGTTTTACCAT	chrX	76812983	76813238
AMPL7153182862	*ATRX*	AAACTGACTTCTGCTGCTTTATGTAC	CTTGGAATCCATCTTATGACATCCAGA	chrX	76813997	76814231
AMPL7153073468	*ATRX*	TTTGTCCAAAGCGATAAACTCTGAATATAC	AGTAAGTAAGAAATGAATTCTCTGAACTCTTGA	chrX	76814174	76814448
AMPL7153073421	*ATRX*	TGACTGTACCTTGCATTTGCTAAAAAC	GCAGAGCGATATATCAAATTCCTTTTGTG	chrX	76829597	76829872
AMPL7153073374	*ATRX*	ATGGATAAGGACAAATAGACATTGCAAAAA	CCTCATATCTCTGGACTTGATTGAAGATTT	chrX	76845152	76845392
AMPL7153073396	*ATRX*	TGTCTTCTCCCTACTAGCTAATTCAAGA	TGCTGGGATTTTTGTTACAATTGTGTTAAA	chrX	76845334	76845517
AMPL7153073311	*ATRX*	TCACATCTTTAGGAAGGAAGGAAAAGC	TGGGAAAATGGTACTTCTCTTTGAAATTCT	chrX	76849053	76849225
AMPL7153073342	*ATRX*	CCCAATTTCCTCTGCCATTCGA	GGGTAGGTCTTTTTCTGTCTCTCTTG	chrX	76849173	76849401
AMPL7153073283	*ATRX*	TCCAAATTACTGTTCCATGATAAAGGCA	TCCTATGCTTTTCTAACAATCTGAATGTGT	chrX	76854834	76855109
AMPL7153073250	*ATRX*	AACGTCAATTATAAGAGGTCAATCAGCA	GCTCGTGCCAATTAACTTATTCTTAAATGA	chrX	76855117	76855387
AMPL7153073191	*ATRX*	TTTTTGTCTAAGTTTATTCTGCTTCCAATAGA	ACTCCTAATCTAGAAACTTGGTTGATATAGACA	chrX	76855842	76856101
AMPL7153073067	*ATRX*	TGCTTCTTGATTTTAATTTTGACAGCATCA	AGTGCAAGCTCTATCAGTACTACTTAGATC	chrX	76871890	76872119
AMPL7153073113	*ATRX*	TCAACAAGGTGTATTGTTTACCTGTTAAGT	GCAATGAGAACTTGTAGTTCATTCCTGTTA	chrX	76872059	76872270
AMPL7153183015	*ATRX*	ACGCAAGGAGATATAACTGTAAAACTATCT	AACGTGCTCACATTCTCTATGAGATG	chrX	76874129	76874317
AMPL7153073027	*ATRX*	ATTTGTACCTGAACACATCCAGCTAA	TGTTTTTGACATGAGCATTTCATTGGG	chrX	76874265	76874539
AMPL7153072853	*ATRX*	TGTTATTCTTTCACAGCAGACTAAGATGA	CAAGGAGGAGGATTATTTTAACAGGAACA	chrX	76875709	76875916
AMPL7153072903	*ATRX*	CTCACACTCAATTAGGTTATTTTGAAGTGG	CTGTAAGATCAGCACTTTAAAGGGATTAAC	chrX	76875857	76876105
AMPL7153072763	*ATRX*	GAATTTTCTAAAAGGCAAAAACCTGAAGGA	GAATGTGAAGAGTCGGAAACTTAAAGAAAT	chrX	76888552	76888750
AMPL7153072809	*ATRX*	CCTGGATCAACCAAAGCTTTGTTAAAT	CAGTGATGCATATTTCAGTGGGAATATTT	chrX	76888693	76888956
AMPL7153072674	*ATRX*	TCTCTCCTGAGGACGTTTCACA	GATGAATGAATTTGAGAAGTGGCAAGA	chrX	76888833	76889108
AMPL7153072714	*ATRX*	CCTCAAGCTTCTCATCATCTTTTAATCCC	TGGATATTTAAACGAAGGTGACAATCTGTT	chrX	76889052	76889239
AMPL7153072599	*ATRX*	GATTAGCAGCTCTCTCCTCCACTA	TGCTGCTGTGAGTCTGTGAAAAA	chrX	76889913	76890174
AMPL7153072631	*ATRX*	ATCCTGAACCTGGAGATTTCTTTGTT	GTTTCTGCTTTAGTTTGGTGGGAAAAA	chrX	76890125	76890368
AMPL7153072576	*ATRX*	TGCCTCACACCCAAATATTGGT	TGGTAAGACTGACCATTGCTTGAAT	chrX	76891339	76891614
AMPL7153040746	*ATRX*	AGAAGAAAGAAATTAGTTACCTCTCTCAATTT	AGGCAGAAAGAAAATTCGGAAGATTCTTA	chrX	76907583	76907736
AMPL7153181322	*ATRX*	CATTTTGTGTTTCTGTTCTCAGTTTATCATCTT	TGGATAAATTAAATGACTTTGTACTGTTTACCT	chrX	76907674	76907897
AMPL7153072512	*ATRX*	AGCATAATTACCATAGTCTACTGTACTGGT	GGAATGCATACAGAGGTAGAACAGTTTTAA	chrX	76909523	76909789
AMPL7153063222	*ATRX*	CCATATAAATCATTTGAAGGCATGGTCATT	CGAGTCAGTGGTAAATTAAGTGAATTAGGT	chrX	76911978	76912221
AMPL7153072484	*ATRX*	CACTGTCTGGTCCAATATGTTCTTTACA	GCATTTTAAAGGCTGAAGAAGGTTTTATTG	chrX	76918821	76919096
AMPL7153072451	*ATRX*	GGGCCATATTAAGATATTTCTTGGTCAGA	CCTCTGATGAGGATGGATCTTCAGA	chrX	76920034	76920223
AMPL7153072465	*ATRX*	TTTTTCCCTTCTTCTGGCTCATCA	AGGTTAAAACATGGTTTTGCTAGTTGTTT	chrX	76920174	76920424
AMPL7153072430	*ATRX*	GGTTTTTACAACCTGCTTGCTGT	CCACTTAGTTTGCTTTAAAGTTTTTGGCTT	chrX	76931623	76931897
AMPL7153040525	*ATRX*	TTTTTATTTATTTTTGCATACCTGAAGATTGGC	TGACATTACATCCTCATCTTCTTCTGATATAGA	chrX	76936990	76937154
AMPL7153072024	*ATRX*	CCCTCACCTATAGAATTCTGATCATCATCT	AAGGAAATACAAAGTGGCTCATCATCA	chrX	76937091	76937309
AMPL7153072039	*ATRX*	CTTCAGAACTTTCCTCAGCATCAGA	CAGAGGATAAAAAGAGTAAGAATGGAGCAT	chrX	76937257	76937518
AMPL7153072053	*ATRX*	CTTGCACCTTTTCTTCTCTCTACCAT	GAACAACAGTATGAATCTTCATCTGATGG	chrX	76937462	76937732
AMPL7153072068	*ATRX*	TCTCGCTCAGGTAACTTTTCAGTG	CTTTGGAAGTTAGAAAAGTTGCTGAAACT	chrX	76937679	76937950
AMPL7153072080	*ATRX*	GGTTTTGAGATGCTTGCTCTTTTCTTT	AGACCTCACAAGAAGGATCATCTGA	chrX	76937894	76938142
AMPL7153072094	*ATRX*	TCTCTCTCTTGTTTTCTTTCAGCATCA	GGCAAATCAGCTAAGAGCTCTATAATTTCT	chrX	76938090	76938365
AMPL7153072122	*ATRX*	CAGACTGGGTTTGTCGTTTCTTTTT	ATTCTGATGAAATGCTAGCAATCCTCA	chrX	76938310	76938559
AMPL7153072150	*ATRX*	TGACTCATCCTGCTCACCTCTT	AGAAACAAAAACTATCAGTTCCAGTGAGAA	chrX	76938510	76938688
AMPL7153072181	*ATRX*	ACTGTCAGAAGAATTACGCTTATCCTTTT	AGAAATGTGGACTTGGACAGGAAAA	chrX	76938629	76938874
AMPL7153072212	*ATRX*	TCATTTTCAACCAAATGCTCATTATCACTG	ACAAGAAGTGGAGAGTTCATCTGTAAAATT	chrX	76938819	76939089
AMPL7153072239	*ATRX*	ACCTCCTCTGTTGTCTTTTGAAGAAATATT	CGGTGGTGAACATAAGAAATCTGATAGAAA	chrX	76939028	76939281
AMPL7153072268	*ATRX*	GGCAGGTTCATATTGAGGTTCTTCT	CGATGGATGCTGTAAACAAAGAGAAAAATA	chrX	76939226	76939492
AMPL7153072298	*ATRX*	ACTTAGCATCTATGACTTTATGCTCTTTGG	CCCAAAGAGATGATTAAGAAGGCAAAAA	chrX	76939432	76939703
AMPL7153072325	*ATRX*	TTGGCTGTGGTCTCAATCAGTT	ACTGCATGTAACAGCGTATTTGAGAA	chrX	76939653	76939916
AMPL7153072353	*ATRX*	TTCTTATTTTGCTGCAACAACTGTTCTAAA	TCAGTTTCCTGAAAGAAGGGAATAATGTTT	chrX	76939860	76940135
AMPL7153063194	*ATRX*	CCTCCCAAAAGTAGGAAACACTGA	CCTGAGCTTGTGTAATATGCTCAATAAATC	chrX	76940381	76940655
AMPL7153071976	*ATRX*	TAGTAGAAGTCTTCCAAGGGCAGAT	AAATGTGTGAATAATAGCCACTCCTTTCT	chrX	76944186	76944454
AMPL7153071960	*ATRX*	GTATTTTTAGTAAGCACATCCGATTTTCCA	GCTTTATCTGAACTGAAGTCTTGTACTTCA	chrX	76949229	76949504
AMPL7153071930	*ATRX*	TTCTAGACAACCACTAGGAAAATGCC	GAAACTGTAAATGAAGATGCGTCTAATGAA	chrX	76951929	76952134
AMPL7153071945	*ATRX*	GCAAGCTCTGCATAGTAATATCATTTTCTG	GTATTGTTCCAGTTGTCAGAAACATTCAT	chrX	76952070	76952248
AMPL7153071915	*ATRX*	GTTAACTCAGAATAGTGGTTGACATGAGT	ATCACTGTATTGAAGTTGCTATAGGGAA	chrX	76952962	76953217
AMPL7153071901	*ATRX*	TGGGTATCAGTAGCCTTCGACA	TACTGCCAGACTTCATAGTGTGAGA	chrX	76953953	76954228
AMPL7153071872	*ATRX*	GGGCTTCTATAAAGCTTGCTAATCTG	CCTCCACGACTTGCAATGAATCA	chrX	76972454	76972638
AMPL7153071887	*ATRX*	AGCACATTCTTTTTCAATTTACCTGTGTTT	TGGCTATATTTTCAGTTTGCTAGAGCTT	chrX	76972585	76972809
AMPL7153071859	*ATRX*	ACCTAAAGAGAAAACGATGCCTCTT	CAGCAGCTACAGTGACGACTAA	chrX	77041328	77041601
AMPL7153180780	*BLM*	GGAACAATGCCTGTTTGAATCAAGT	GAGAAGATATCTCATGTGTACAATGTGGT	chr15	91290516	91290791
AMPL7153415096	*BLM*	GGGTTTCTTAAAATGGATCCATCTAATCT	GTGTTGGGTAGAGGTTCACTGA	chr15	91292546	91292737
AMPL7153415097	*BLM*	AAGATGTTAATGTTACCGAAGACTTTTCCT	GATGGTACTTAAAGAATCTGGTGAAGAACT	chr15	91292685	91292951
AMPL7153415098	*BLM*	CGGGATACTGCTCTCAAGAAATTAGAATTT	GGGTGGAGGCAAATCAGTCTTTAC	chr15	91292891	91293131
AMPL7153415099	*BLM*	TTTAAAGCACAGCTTTATACAACAAACACA	ATTGTTACCTCTTTCATCTTCCAAATGAGT	chr15	91293077	91293305
AMPL7153137254	*BLM*	CAAAGTGGTGTGATTGTTTGTGACT	CATTTTGAAGAGGAAGAAGAAGCAGAAATA	chr15	91294898	91295170
AMPL7153137257	*BLM*	TTTTGTTCCACCTTCTCCAGAAGAAAT	TTTTTGAGGCTTTCACTTGAATGTAAAATA	chr15	91295113	91295316
AMPL7153415094	*BLM*	ATTGAGTCTAGCCTATAGTATGATTGGCT	CACAATCTTTGTGTTACGTTGTTTTCAAAA	chr15	91297990	91298242
AMPL7153137236	*BLM*	GCTAGACAGATAAGTTTACAGCAGCA	CTGTCCATTTTTCACTTAGAGTGGTAGAAATAT	chr15	91303375	91303579
AMPL7153415110	*BLM*	CTTTTCAAACCACTCCTTTAACATTCGT	TGAATCAGGCCTGTATCTCCACA	chr15	91303655	91303905
AMPL7153137223	*BLM*	GATGCCAGTCTTCTTGGCTCAT	CCCAGTTGCTACTTACAAAGGACTTC	chr15	91303860	91304119
AMPL7153137225	*BLM*	TGAAAGGCCTTTATTCAATACCCATTTACA	GCTGGCTGCTAAATTATGCATTATGTC	chr15	91304063	91304334
AMPL7153415111	*BLM*	GACTTTGATGATGATGATGACTGGGAA	GGGACAGTTTTGTTTTCATATAAGCATTCA	chr15	91304280	91304532
AMPL7153415103	*BLM*	TTAAGAACTGTGCTGCAGGGAAA	CTCATGTTTCAGATTTCTGGATGCTAAATT	chr15	91306076	91306239
AMPL7153415104	*BLM*	TGATTATTTTCCTAGACAAGTCAGCACAA	TGGATTCCTGCCAATTCACTTCTAAAAATA	chr15	91306180	91306422
AMPL7153415108	*BLM*	TGTAACTTTTACATTCATGCTCTGAAGACA	ACTGAAATTTAATTGTTTCTAAGGCGTGTT	chr15	91308459	91308698
AMPL7153415102	*BLM*	GCCCTGCCTGAGTTATGCTTAA	GGTAAATATTTGTAGCTTCTGAGTCAGTCT	chr15	91309949	91310197
AMPL7153137196	*BLM*	TTCCAGCTACATATCTGACAGGTGATA	GGATGAAAGTTGTACAAAAATTCTTTGCAC	chr15	91310140	91310371
AMPL7153137185	*BLM*	TAAGTTGTGATGGAATTTGAAGACCACA	TCACTCAGTGTGGGTTTTTATTCAGTAAAT	chr15	91312277	91312551
AMPL7153415112	*BLM*	TCTTAATACATTGAGCAGTGTTGGCTT	CCCAGGGTTCTCAAATACGTGA	chr15	91312585	91312853
AMPL7153415101	*BLM*	GCAAGGTCTCTACTTATTTTTGAGCAC	ATCAAATGCCACCTTTTTAGGCTTTTT	chr15	91325852	91326127
AMPL7153137171	*BLM*	GGTTTAGCATGAGCTTTAACAGACATAATC	TGCTGTCATAATGCAAAAAGGAATGTTTAA	chr15	91326050	91326268
AMPL7153415100	*BLM*	AATTCTTGTTTCTCAGTACTCTTGGTTTCT	TCTACATGTGCATGTTTGGTTATATGCTAT	chr15	91328114	91328383
AMPL7153415095	*BLM*	GTGCCTTATGAATCTAATAAGCTTTTGCT	GAAGATGACAGAATGTCTCTACAATGGAG	chr15	91333839	91334113
AMPL7153415106	*BLM*	TGACAATGCTGTTAACAGTAATTTTCTTATTT	TTTTCACAGTAATGTACCATGCTATACAAATTA	chr15	91337267	91337472
AMPL7153415107	*BLM*	AACCATCATACAAGAGAAACTCACTTCAA	AGACCACCTTTTGCAATCTAATTTGTATCT	chr15	91337410	91337679
AMPL7153415093	*BLM*	AATCTAGGCATTGTTACCTTAATTATAGCAG	AACTCAAACATTCAAAACAGATGACTTAC	chr15	91341364	91341596
AMPL7153415092	*BLM*	GGTTTGTGATCTATTCCATCTGTCCA	ATTGTGTCGTGAATAAGCAGATCCTT	chr15	91346632	91346815
AMPL7153137310	*BLM*	GCAAAAATCCAGTCAGGTATATTTGGAA	GCTTGGACAAAGACACTATACTTCACAATA	chr15	91346761	91347036
AMPL7153415105	*BLM*	GCACATATACCCACTCCTATGATTTGT	TTAATCTGTGCCACGTAACAAAGGA	chr15	91347354	91347629
AMPL7153137289	*BLM*	AATGAGCCTGAATTCAGTGGGTT	AACTGCATCCTTCTAATTCAGTGAGAC	chr15	91352297	91352570

AMPL7153415109	*BLM*	TCACCTGTAAACATCTGCATTTTCCA	CCTTGTTCCCTATATTGAAAAAGATGTCAC	chr15	91354401	91354675
AMPL7153415113	*BLM*	GCAGAAAATGCACAAGGACACATT	GCTGGAGGATTTCGTTTTGGAAGA	chr15	91358205	91358380
AMPL7153415114	*BLM*	GGGTCTGCCACATGTAGAAAGATA	CAAGAAAGAGGGTCTATGTACATTGAGATT	chr15	91358332	91358545
AMPL7153004227	*BRCA1*	GACAGTAGAAGGACTGAAGAGTGAG	AGAATGAATTGACACTAATCTCTGCTTGT	chr17	41197588	41197863
AMPL7153004403	*BRCA1*	AAATATTTAAAATGTGCCAAGAACTGTGCT	AAAAATGATGAAGTGACAGTTCCAGTAGT	chr17	41199548	41199793
AMPL7153004264	*BRCA1*	GGCAGAGAAGACTTCTGAGGCTA	CTTCATCCGGAGAGTGTAGGGTA	chr17	41201020	41201295
AMPL7153004237	*BRCA1*	CCCATCGTGGGATCTTGCTTAT	AAAGCTCTTCCTTTTTGAAAGTCTGTTTTT	chr17	41202995	41203246
AMPL7153004216	*BRCA1*	GTGAAAGTATCTAGCACTGTGTATGTATGT	ACTTCCATTGAAGGAAGCTTCTCTT	chr17	41208939	41209214
AMPL7153004435	*BRCA1*	ATCATGGAAAATTTGTGCATTGTTAAGGAA	CCTCTCCTCTGTCATTCTTCCTGT	chr17	41215230	41215504
AMPL7153004897	*BRCA1*	GTAACTCAGACTCAGCATCAGCA	AAAACTGAGGCTCTTTAGCTTCTTAGG	chr17	41215783	41216054
AMPL7153004299	*BRCA1*	GCCTCGCCTCATGTGGTTTTAT	TAACTAGTATTCTGAGCTGTGTGCTAGA	chr17	41219562	41219778
AMPL7153004194	*BRCA1*	AAAACTCTTTCCAGAATGTTGTTAAGTCTT	TGATACTGCTGGGTATAATGCAATGG	chr17	41222881	41223071
AMPL7153004201	*BRCA1*	CTTCTCCCTGCTCACACTTTCTT	CGTTGTGTAAATTAAACTTCTCCCATTCCT	chr17	41223022	41223290
AMPL7153004391	*BRCA1*	AAAATCAAAGTGTTTGTTCCAATACAGCA	CAATTGGTGGCGATGGTTTTCTC	chr17	41226305	41226580
AMPL7153004289	*BRCA1*	AAATAAAGATGTCAGATACCACAGCATCTT	CCTGAATTATCACTATCAGAACAAAGCAGT	chr17	41228456	41228693
AMPL7153004180	*BRCA1*	CCAGAACCACCATCTTTCAGTAATTTG	CTTGTTTACAGCGATGCCAACA	chr17	41231286	41231552
AMPL7153004374	*BRCA1*	GGGAAGGAAAGAATTTTGCTTAAGATATCA	CTCAAAGTATTTCATTTTCTTGGTGCCAT	chr17	41234368	41234636
AMPL7153004417	*BRCA1*	AGATGATGTCAGCAAACCTAAGAATGT	CCAGTCCTGCCAATGAGAAGAA	chr17	41242865	41243139
AMPL7153004506	*BRCA1*	GGGCAAACACAAAAACCTGGTT	CCTTAGTGAGGAAACAAAATGTTCTGCTA	chr17	41243422	41243696
AMPL7153004511	*BRCA1*	ACTGCACTGTGAAGAAAACAAGC	AGTCCTCAGAAGAGAACTTATCTAGTGAG	chr17	41243644	41243919
AMPL7153004518	*BRCA1*	GGAAGCAGGGAAGCTCTTCATC	TGCATCTCAGGTTTGTTCTGAGAC	chr17	41243868	41244125
AMPL7153004524	*BRCA1*	ACCATCATCTAACAGGTCATCAGGT	ATTCAAGCAGAACTAGGTAGAAACAGAG	chr17	41244076	41244346
AMPL7153004530	*BRCA1*	CCTAATCTAAGCATAGCATTCAATTTTGGC	GGAAAACTTTGAGGAACATTCAATGTCA	chr17	41244287	41244548
AMPL7153004535	*BRCA1*	TGTTCTCATTTCCCATTTCTCTTTCAGG	GCTTTCCTGTGGTTGGTCAGAAA	chr17	41244492	41244765
AMPL7153004542	*BRCA1*	CTACATTTGGCATTATCAACTGGCTTATC	GCTCAGTATTTGCAGAATACATTCAAGG	chr17	41244713	41244988
AMPL7153004547	*BRCA1*	GCAAATGACTGGCGCTTTGAAA	CAAAAACAGAACCAAATAAATGTGTGAGTC	chr17	41244938	41245167
AMPL7153004553	*BRCA1*	GGGTTTTCAAATGCTGCACACT	CCCAAAGATCTCATGTTAAGTGGAGAAAG	chr17	41245115	41245309
AMPL7153004560	*BRCA1*	CTCTACAGATCTTTCAGTTTGCAAAACC	CAACTGGAGCCAAGAAGAGTAACA	chr17	41245252	41245524
AMPL7153004565	*BRCA1*	GTCTTTTACTTGTCTGTTCATTTGGCT	GAGGAAGTCTTCTACCAGGCATATTC	chr17	41245473	41245712
AMPL7153004570	*BRCA1*	CTACTGACTACTAGTTCAAGCGCAT	GAGCAGAATGGTCAAGTGATGAATATTACT	chr17	41245661	41245930
AMPL7153004576	*BRCA1*	TCACCTTTTGTTTTATTCTCATGACCACTA	CCCAACTTAAGCCATGTAACTGAAAATCTA	chr17	41245868	41246134
AMPL7153004583	*BRCA1*	GTGGCTCAGTAACAAATGCTCCTATAAT	AGGTAGATGAATATTCTGGTTCTTCAGAGA	chr17	41246076	41246295
AMPL7153004588	*BRCA1*	GGATCACTGGCCAGTAAGTCTATTT	CCCTGTGTGAGAGAAAAGAATGGAATAAG	chr17	41246240	41246511
AMPL7153004593	*BRCA1*	GATTCTCTGAGCATGGCAGTTTC	TGTGGCACAAATACTCATGCCA	chr17	41246457	41246728
AMPL7153004600	*BRCA1*	GCTGTTCTCATGCTGTAATGAGC	CACCTCCAAGGTGTATGAAGTATGTATTTT	chr17	41246683	41246958
AMPL7153004343	*BRCA1*	AGGTCCCAAATGGTCTTCAGAATAATC	GCTTTCTGTAATCGAAAGAGCTAAAATGTT	chr17	41247768	41248043
AMPL7153004868	*BRCA1*	ATAGGAAAATACCAGCTTCATAGACAAAGG	GCCACAGTAGATGCTCAGTAAATATTTCTA	chr17	41249215	41249428
AMPL7153004948	*BRCA1*	GCCTACCACAAATACAAATTATGACCAAG	TTCTCTTCAGGAGGAAAAGCACAG	chr17	41251690	41251965
AMPL7153004307	*BRCA1*	AGGAGGACTGCTTCTAGCCT	CCAAAAGACTTCTACAGAGTGAACCC	chr17	41256029	41256179
AMPL7153004311	*BRCA1*	ACAAATGGTTTTACCAAGGAAGGATTTTC	CTAACTGCAAACATAATGTTTTCCCTTGTA	chr17	41256124	41256316
AMPL7153004325	*BRCA1*	TTGTGCAAACTTCCTGAGTTTTCATG	GGTTTTCTACTGTTGCTGCATCTTATTTTT	chr17	41256817	41257091
AMPL7153004167	*BRCA1*	TGATGAATGGTTTTATAGGAACGCTATGT	CAGTTGTGAGATTATCTTTTCATGGCTATT	chr17	41258375	41258649
AMPL7153004280	*BRCA1*	CTTCTATAAAGTTAGGTGTTTCCTGGGTT	TCAGTCATAACAGCTCAAAGTTGAACTTAT	chr17	41267658	41267903
AMPL7153004135	*BRCA1*	AGCCTAATCTTACTAGACATGTCTTTTCTT	CGCGTTGAAGAAGTACAAAATGTCATTAAT	chr17	41275918	41276095
AMPL7153004143	*BRCA1*	GGGACACTCTAAGATTTTCTGCATAGC	GACGTTGTCATTAGTTCTTTGGTTTGTA	chr17	41276038	41276276
AMPL7152997061	*BRCA2*	TTTTACCTCAGTCACATAATAAGGAATGCA	GGTTCTAAGCAACACTGTGACGTA	chr13	32890491	32890764
AMPL7152996459	*BRCA2*	CTTTAACTGTTCTGGGTCACAAATTTGT	TTCAGATTCTTCTGCAGGTTCAGAATT	chr13	32893142	32893299
AMPL7152996984	*BRCA2*	CTTTCTTCAGAAGCTCCACCCTAT	GTTTGCCTAAATTCCTAGTTTGTAGTTCTC	chr13	32893248	32893519
AMPL7152997004	*BRCA2*	CCTCCCAGAGCCCTCAAATTATAA	CGAAGACTTTTATGTCTACTATTGGGAACA	chr13	32899005	32899249
AMPL7152997005	*BRCA2*	ACTGAATTATTGTACTGTTTCAGGAAGGAA	CAGGCTCTTAGCCAAAATATTAGCATAAAA	chr13	32899189	32899430
AMPL7152996997	*BRCA2*	CGGTTTCCAGCAGCTGAAATTTG	TGGTGTTACATGTGTACATTGTAGAACA	chr13	32900007	32900271
AMPL7152996998	*BRCA2*	GGGATTTGCTTTGTTTTATTTTAGTCCTGT	ATGAAACAAACTCCCACATACCACT	chr13	32900213	32900401
AMPL7152996993	*BRCA2*	CAGGCAATTCAGTAAACGTTAAGTGAAA	ACAATTATCAACCTCATCTGCTCTTTCT	chr13	32900529	32900804
AMPL7152997002	*BRCA2*	CAAATAGTAGATGTGCTTTTTGATGTCTGA	GGTTTAGAGACTTTCTCAAAGGCTTAGAT	chr13	32903487	32903761
AMPL7152997059	*BRCA2*	TTTTTGGACCTAGGTTGATTGCAGA	AAAAACCTGTAGTTCAACTAAACAGAGGA	chr13	32904928	32905201
AMPL7152996448	*BRCA2*	ATGGCTTATAAAATATTAATGTGCTTCTGTTTT	AGGTATCTACAACTGTTTCATATACTTCATCTT	chr13	32906363	32906522
AMPL7152996977	*BRCA2*	GGAAAGTCAATGCCAAATGTCCTAG	GGCTTCTGATTTGCTACATTTGAATCTAAT	chr13	32906464	32906739
AMPL7152996979	*BRCA2*	TGAAGTGGAACCAAATGATACTGATCC	GCTCCATTTAGACCTGAAAGGGTTAG	chr13	32906682	32906832
AMPL7152996981	*BRCA2*	TTTGGCCTGTGAATGGTCTCAA	TGCAGTCTGTATGAGATTCAAGATGC	chr13	32906784	32907056
AMPL7152996982	*BRCA2*	CAGTGGTAAATAAGAGAGATGAAGAGCA	TCTGTGAGCAAACAGTATGTATTTCCAG	chr13	32907002	32907260
AMPL7152996983	*BRCA2*	GAAACTGAAGCCTCTGAAAGTGGA	CTTCAAACTGGGCTGAACAGTTAATTA	chr13	32907208	32907479
AMPL7152996458	*BRCA2*	AAAAATACCGAAAGACCAAAAATCAGAAC	CACAGAAGGAATCGTCATCTATAAAACTAT	chr13	32907423	32907589
AMPL7152997015	*BRCA2*	GAAAAATATTTAGTGAATGTGATTGATGGTACT	GTCCCAAAAGAGCTAGTTAAGGACAA	chr13	32910336	32910486
AMPL7152997017	*BRCA2*	TTCACAGAATGATTCTGAAGAACCAACT	GCCAAGACCTCTTCTTTTATATCTGAAACT	chr13	32910432	32910696
AMPL7152997014	*BRCA2*	CAGTGTGAAAATGATCCAAAAAGCAAAAA	CAGAACATCCTTGGAAGTAGGAGTTAAAAT	chr13	32910637	32910829
AMPL7152997016	*BRCA2*	TATATGATCATGAAAATGCCAGCACTCTT	CTTGAAGGTGATGCTACTCTCATGT	chr13	32910770	32911032
AMPL7152997018	*BRCA2*	GAGCTGTTGCCACCTGAAAAAT	AAAATGGGTTCGTTTACACAAGTCAAG	chr13	32910985	32911257
AMPL7152997019	*BRCA2*	AGGAAATACTAAGGAACTTCATGAAACAGA	CCTCTGCAAGAACATAAACCAAATCTTT	chr13	32911200	32911352
AMPL7152997023	*BRCA2*	AAACAAGCAACCCAAGTGTCAATTAAA	CCAAAACTGTGATTTGAAATTGGACCT	chr13	32911297	32911509
AMPL7152997025	*BRCA2*	ATGAACAAATGGGCAGGACTCTT	AGGCTTGCTCAGTTTCTTTTGATTATCTA	chr13	32911459	32911678
AMPL7152997022	*BRCA2*	CTTGTGTTGAAATTGTAAATACCTTGGCAT	TCTGCCTTTTGGCTAGGTGTTAAATTA	chr13	32911619	32911821
AMPL7152997029	*BRCA2*	ATTTTCCAAGCAGGATTTTAATTCAAACCA	GCATTCCTCAGAAGTGGTCTTTAAGAT	chr13	32911764	32911969
AMPL7152997031	*BRCA2*	AAGTGCCTGAAAACCAGATGACT	CAGAGCTTCAGTAGAAACATTCAGTTTTG	chr13	32911919	32912194
AMPL7152997033	*BRCA2*	GGCTTTTATTCTGCTCATGGCA	ATTGAAACAACAGAATCATGACATTTACTTGAA	chr13	32912143	32912307
AMPL7152997028	*BRCA2*	TTCTGCAGAGGTACATCCAATAAGTTTATC	TACTTGAATCACTGCCATCAAATTCTAAGT	chr13	32912244	32912519
AMPL7152997030	*BRCA2*	AAATATACTGCTGCCAGTAGAAATTCTCAT	TTGAGCTTTCGCAACTTCCAAAAA	chr13	32912458	32912680
AMPL7152997032	*BRCA2*	GGGAAACACTCAGATTAAAGAAGATTTGTC	GCAATTCTTCTGGTTTCTGATCAAAGAAAT	chr13	32912616	32912864
AMPL7152997038	*BRCA2*	GAAAAATATTAGTGTCGCCAAAGAGTCATT	GAAAACCCAATAGAGTAGGTTCTTTGATCT	chr13	32912790	32913065
AMPL7152997040	*BRCA2*	GGACAACCCGAACGTGATGAAA	GAGAGAATTCTGCATTTCTTTACACTTTGG	chr13	32913013	32913286
AMPL7152997037	*BRCA2*	GTGAGACCATTGAGATCACAGCT	GGCTGAATTTTCAATGACTGAATAAGGG	chr13	32913230	32913496
AMPL7152996537	*BRCA2*	GTCCTGCAACTTGTTACACAAATCAG	GACCATCAAATATTCCTTCTCTAAGCCAT	chr13	32913440	32913593
AMPL7152997039	*BRCA2*	TGAGTCAGACTTCATTACTTGAAGCAAAAA	GAATAGCTGTTAGACATGCTACTGTTACTT	chr13	32913533	32913741
AMPL7152997041	*BRCA2*	GACAAAAATCATCTCTCCGAAAAACAAGAT	CCTCAACGCAAATATCTTCATTTACAGTT	chr13	32913673	32913926
AMPL7152997042	*BRCA2*	GTAAAAGATGCAAATGCATACCCACA	TGTGAAACACAAACGATTTTACCACTG	chr13	32913871	32914059
AMPL7152997046	*BRCA2*	GGCCACCTGCATTTAGGATAGC	CTGAATGTCAGCAAAAACCTTATGTGA	chr13	32914010	32914267
AMPL7152997049	*BRCA2*	CTCTAGATAATGATGAATGTAGCACGCAT	GCATCTGATACCTGGACAGATTTTCC	chr13	32914211	32914464
AMPL7152997045	*BRCA2*	GTGGGATTTTTAGCACAGCAAGT	ACTTGCTTTCCACTTGCTGTACTAAA	chr13	32914415	32914689
AMPL7152997047	*BRCA2*	GTGGTAAATTCATCTGCTTTCTCTGGA	CCATTTCTGAGTTTACACAGTGCTCT	chr13	32914636	32914859
AMPL7152997048	*BRCA2*	ACTTCCTCGTGTTGATAAGAGAAACC	ACTTTGGTTCCTAATACCAACTGTTGTT	chr13	32914806	32915004
AMPL7152997050	*BRCA2*	CTCCATATCTCTCTCAATTTCAACAAGACA	TGTCAGTTCATCATCTTCCATAAAAGCTTT	chr13	32914946	32915215
AMPL7152997051	*BRCA2*	CTTTGAAACAGAAGCAGTAGAAATTGCT	GGCAACACGAAAGGTAAAAATGAACA	chr13	32915157	32915364
AMPL7152996991	*BRCA2*	TGACATTGAAGACTGACTTTACTCTTTCAA	GCTTTTTGAAGCCTTTAAGGATTTTTCTTG	chr13	32918519	32918780
AMPL7152996992	*BRCA2*	AGGAGAACCCTCAATCAAAAGAAACTTATT	TATAAAGAGGTCCTTGATTAGGCACAGT	chr13	32918692	32918931
AMPL7152997006	*BRCA2*	TATGCTGATTTCTGTTGTATGCTTGTACT	GGCTCTAAAGAAACATGATGCATAAACAAT	chr13	32920740	32921012
AMPL7152997007	*BRCA2*	GTTTCCTAGGCACAATAAAAGATCGAAG	GGAAGTGTTAACTTCTTAACGTTAGTGTCA	chr13	32920954	32921145
AMPL7152997052	*BRCA2*	CCCATTGCAGCACAACTAAGG	CTAACACACTGTTCAACTCTGTGAAAAT	chr13	32928987	32929261
AMPL7152997053	*BRCA2*	CCAAAGTCTTTGTTCCACCTTTTAAAACTA	GGCAAAAATTCATCACACAAATTGTCATAC	chr13	32929197	32929460
AMPL7152996975	*BRCA2*	TGATAGATTTAATTACAAGTCTTCAGAATGCCA	TCATAAAAGCCATCAGTATTGTAGACAAACA	chr13	32930558	32930782
AMPL7152997062	*BRCA2*	TTTTTGTGTGTGTTTATTTTGTGTAGCT	ACATAAAAGTTAACACACAATCTTTTTGCA	chr13	32931852	32932104
AMPL7152997056	*BRCA2*	GTTGTTGAATTCAGTATCATCCTATGTGGT	TGCCGTATATGATTACGTAATGTAATGCT	chr13	32936596	32936869
AMPL7152996989	*BRCA2*	TTGTTTAAACAGTGGAATTCTAGAGTCACA	CATCTGTAAGTTCAATAATGGCCACTTTT	chr13	32937250	32937509
AMPL7152996990	*BRCA2*	GCAATAAAACTAGTAGTGCAGATACCCA	AATGACTGATTTTTACCAAGAGTGCAAATT	chr13	32937452	32937707
AMPL7152996995	*BRCA2*	AGACTTTTTAAAGTGAATATTTTTAAGGCAGTT	GTCTAGGGTCAGGAAAGAATCCAAG	chr13	32944418	32944605
AMPL7152996478	*BRCA2*	CCTGCTCGCTGGTATACCAAA	ATGGTAAGTTTCAAGAATACATCATACCTG	chr13	32944559	32944721
AMPL7152997057	*BRCA2*	GTGCCTGGCCTGATACAATTAAC	CCTCCTGAATTTTAGTGAATAAGGCTTCTA	chr13	32945019	32945219
AMPL7152997058	*BRCA2*	TATGTGGAGGCCCAACAAAAGAGAC	TGTTGCTATTCTTTGTCTAACACCAAAAA	chr13	32945164	32945409
AMPL7152996999	*BRCA2*	TGGGTGTTTTATGCTTGGTTCTTTAGT	AGAGAGTCTAAAACAGCTTCTCACCT	chr13	32950723	32950998
AMPL7152997009	*BRCA2*	CCACACCCTTAAGATGAGCTCT	CAGATTCCATGGCCTTCCTAATTTC	chr13	32953332	32953562
AMPL7152997011	*BRCA2*	TGATAAGAAACAAGCTCAGATCCAGTTG	GCATTAGTAGTGGATTTTGCTTCTCTGATA	chr13	32953509	32953783
AMPL7152996988	*BRCA2*	TGATAATCACTTCTTCCATTGCATCTTTCT	ATGGAGATTCCATAAACTAACAAGCACTT	chr13	32953839	32954114
AMPL7152997000	*BRCA2*	AAATCTGAAAGAGCTAACATACAGTTAGCA	CTCAGAACAAGATGGCTGAAAGTCT	chr13	32953987	32954239
AMPL7152997001	*BRCA2*	CCCTTCACTTCAGCAAATTTTTAGATCC	CTGGTAGCTCCAACTAATCATAAGAGATTT	chr13	32954186	32954370
AMPL7152996954	*BRCA2*	GAGTTTCCTTTCTTGCATCTTAAAATTCA	GAGGTTGCTTGCAGCAATTAACATA	chr13	32968750	32968944
AMPL7152996955	*BRCA2*	GACCTTAATGAGGACATTATTAAGCCTCA	AAATGTGTGGTGATGCTGAAAAGTAAC	chr13	32968890	32969102
AMPL7152997003	*BRCA2*	TATGTGGGTTTGCAATTTATAAAGCAGC	TATACAACAGAATATACGATGGCCTCCATA	chr13	32970986	32971251
AMPL7152996985	*BRCA2*	TTTTTATCAGATGTCTTCTCCTAATTGTGAG	GAGGTAAAGGCAGTCTACTCAAGAA	chr13	32972288	32972491
AMPL7152996986	*BRCA2*	TGCAAAAAGAGAAGAGCCTTGGAT	AACCAGACAAAAGAGCTTGGGTATTTA	chr13	32972442	32972716
AMPL7152996987	*BRCA2*	TAGCTGACGAAGAACTTGCATTGA	CGTCAATAATTTATTGTCGCCTTTGCA	chr13	32972665	32972939
AMPL7153122854	*DCLRE1C*	GACTGTCATCTCTGTGCAGGTT	TGAGTTACCTAAACGAGAGCATTTACAAT	chr10	14950347	14950509
AMPL7153194942	*DCLRE1C*	ACCAGTTGCCAGCTTCTCATATAAAT	TCATGGAACAAAATGTAATTTGCCCAA	chr10	14950454	14950717
AMPL7153775018	*DCLRE1C*	CTCTGCTTTTCAAATCAGAGTAAGTATCCT	CTTTTCAGTGACTCTGATGGAGAATCA	chr10	14950660	14950925
AMPL7153775019	*DCLRE1C*	AGAATTCTGGGAGGAGATGTGAGT	ACAAACTTTGTAGATTGTGAAGAATCCAAC	chr10	14950874	14951147
AMPL7153775020	*DCLRE1C*	GGATTCCTACTTCTTCTTCACTTTCACT	TTGTTTCCTATAAACCTGTTTTGTTTGTCC	chr10	14951089	14951364
AMPL7153208895	*DCLRE1C*	CCTCTGAACCCAGGACCATTTT	TACCTTCTAACAACTTCCTCAAAAGGAAAA	chr10	14961708	14961885
AMPL7153142471	*DCLRE1C*	CTCTCCTTTGTGTCCTAGCCAA	GAGAACCACAGCCATAAGAGAGTTC	chr10	14964911	14965182
AMPL7153142764	*DCLRE1C*	GGGACTACCTGTCAACTACCAAG	CCTAAATGAGAAGGGCCAGCTTT	chr10	14968768	14968976
AMPL7153208853	*DCLRE1C*	TACTCAAAGTTTCTCTCAGAAGACCTATGA	TGCCTACAACAGATTTGTAGAAAATGTAGA	chr10	14969970	14970212
AMPL7153208638	*DCLRE1C*	ATTACACAAACAATACGAGAGGAATCACT	AACTACTTTTCAAGAAAGAAGAGAGGGTTA	chr10	14974812	14975081
AMPL7153775021	*DCLRE1C*	GAAAAGAAAAGAATGAACAGTCACCATACC	CCACCTGTTAAATGTCTCTTTAAAATCCTG	chr10	14976349	14976554
AMPL7153142898	*DCLRE1C*	GACCTGTCACCCTACAAACTTCC	GGTAGTGAGCAGACATTGCACTT	chr10	14976623	14976898
AMPL7153142715	*DCLRE1C*	CACAGCAAGACTCCATTTCACAAAAAT	CTCACATGTATTTGCTTTATTGGGAGTTTA	chr10	14977396	14977633
AMPL7153142290	*DCLRE1C*	AAAAATCAAATCTCCCTCTATGTATTCTTTCT	CTGGTCACTGTCCGGGAT	chr10	14978290	14978561
AMPL7153095036	*DCLRE1C*	CCTTACATAACTGATCCCGGACA	GTGTTCTGTGAAGGAGGGCTTT	chr10	14978530	14978676
AMPL7153142434	*DCLRE1C*	CAAAATCAAAGAGAAAGATTGAGGGTATAGC	CCATTTCTGTGTTTCAGACAGCA	chr10	14981678	14981950

AMPL7153142514	*DCLRE1C*	ATAACTGAAGTATGTTACAAACTGAGGCAA	GATTTCTTAGTCACCAAGATGGCTCA	chr10	14987000	14987255
AMPL7153142669	*DCLRE1C*	GGGTGATGCGTTCATTTTGTAAATGA	ACCTCATGGGTTTTCTCTTGAAGC	chr10	14990918	14991193
AMPL7153194885	*DCLRE1C*	GATGAAGCCTTTGAGAGGCTGAA	GGCCGAGTATCCAACTATCTCCA	chr10	14995732	14995986
AMPL7153194892	*DCLRE1C*	TTCTCCCTATCGAAGCGGTCTA	CCGGACTCCTCTGATTGGAC	chr10	14995941	14996153
AMPL7153734010	*EME1*	TGAATGCTCCAGTCTGTGTCAT	CCTTCTTCAGAAAGGCAAATGTTGG	chr17	48452504	48452654
AMPL7153734654	*EME1*	GGTGATAGTGACTCTGAGGAGTTG	TTTGAGCCAGAGGAATAAATTCTTCTTCAT	chr17	48452605	48452864
AMPL7153734655	*EME1*	GGTTGCTAAGCAGTGAAAGTGAAG	AGCATGGATCTAAGATCAGGTCCTT	chr17	48452810	48453068
AMPL7153734656	*EME1*	CAGAGAGGAGTGCAGCAGATAAC	GGCTTTCATCCTGGTAACCAGT	chr17	48453020	48453295
AMPL7153734657	*EME1*	CTGAGAAGACAGGAAAGAAAGAATGCAG	CAGATGGAAAAGGGACTGTCAGT	chr17	48453244	48453420
AMPL7153734662	*EME1*	CTTAAAACACATCATTGTAGTGCTGGAT	GGGAGGAAATGTCAGCCAGAAAA	chr17	48453312	48453584
AMPL7153734661	*EME1*	GGGTGGGAGTTAGTTCATTCACC	GGGCAGAAACTGCATTAATACTTGC	chr17	48455852	48456124
AMPL7153734653	*EME1*	ACAGAGGGCTGACTCTTACTAGG	AGTGACCTACATCTGCAATGAATAAACT	chr17	48456074	48456349
AMPL7153734651	*EME1*	GATGCTCTGGTCCAGTCTTCTT	AGTCTCAGGTGACCAGTGAGTAC	chr17	48456383	48456640
AMPL7153734659	*EME1*	GTGTGGCTTTAGTGGACAGTGA	GGGACATTTGCTTTGACTGCAA	chr17	48456734	48457006
AMPL7153734658	*EME1*	GGGAACAGGTGTAGAAGAGTAATGAG	GGATAGGCATTCACAACTGCACT	chr17	48457614	48457840
AMPL7153734020	*EME1*	GAACCGAGTCAGCCTGGAAAT	CAACAGTTGTCCATGGAAATCTGG	chr17	48457792	48457943
AMPL7153734660	*EME1*	GGATCACCATGGATGGTACCAT	GGGAAGTGGAAGTTTCCAGCTT	chr17	48458075	48458348
AMPL7153496376	*ERCC1*	CTGAGGAACTAAAGAAAGCTGAAGGT	GATTTACCCAGGAAACAGCTCCTT	chr19	45912774	45913046
AMPL7153496378	*ERCC1*	TTTTTATTTTTAGTAAAAATGAGGTGTCCACTT	CCCAAATAAACACAACCTGAGACC	chr19	45916705	45916887
AMPL7153496379	*ERCC1*	ACTGTGTCTGGTCTTTGGTTCTTTTT	GGGAGGCTTTTGTGCTCAACT	chr19	45916820	45917052
AMPL7153150363	*ERCC1*	GTGGGCAGGGAGATGGAAGGAAAT	AGTCTTGCTGGCCTTTCTTCTTC	chr19	45917128	45917344
AMPL7153150369	*ERCC1*	GTGCCTTAAAATTGGAACTGAAGCT	CTAGTATTGGCAATTCTTATGACTGACCAT	chr19	45918035	45918310
AMPL7153141695	*ERCC1*	CCAGGTGGAGCCTGAATGAG	TTTTTGTAATTCCTGGCTTCTAGGTTTCT	chr19	45920037	45920205
AMPL7153150375	*ERCC1*	CTGCATTTCCTCTTGGAAGGGAT	CCCTTAGTATTCCAGTGAGAGGGAA	chr19	45922269	45922542
AMPL7153150380	*ERCC1*	GTCCAGAACACTGGGACATGA	CCCTGTGGTTATCAAGGGTCAT	chr19	45923502	45923743
AMPL7153150358	*ERCC1*	GAGTCATCCCTAGAACAGCGTT	CCTCAGACCTACGCCGAATATG	chr19	45924367	45924588
AMPL7153150360	*ERCC1*	CCTTCCAGAGGCTGTGAGATG	ACTCACAGGGCCTCAGATGT	chr19	45924544	45924720
AMPL7153152476	*ERCC1*	CCCATCCTATCCTCTTCGTCCTA	CTGCGGGATGAGAACGTAGAC	chr19	45926461	45926698
AMPL7153278104	*ERCC2*	AAGACTCAGGAGTCACCAGGAA	AAATTCATTCAAACATCCTGTCCCTACT	chr19	45854839	45855098
AMPL7153415129	*ERCC2*	CTGGCACGTAGATGCACGATA	CAGATGCCAACCTCAACCTGA	chr19	45855320	45855552
AMPL7153414556	*ERCC2*	GTACTTGGCCACCTGGACA	CCGAATGACCTTCTGTCCCTG	chr19	45855499	45855651
AMPL7153389919	*ERCC2*	ACCTGGGAAATGAACGGGAAAC	CCCAGCTTCTCATCCTCCGTAT	chr19	45855687	45855935
AMPL7153415159	*ERCC2*	GTAAGAAAGTCATTCTCACGAATCTGGA	CCCACCAGCCACACTTGTA	chr19	45855851	45856125
AMPL7153414577	*ERCC2*	CCAGAGAGCTCTGGGAAGACA	GTGGCTCTGAGGAGTGACCTA	chr19	45856290	45856461
AMPL7153415161	*ERCC2*	GCAAGTTAGGTCACTCCTCAGA	CAGAAGAGTTGGATGTAACCAGGAT	chr19	45856434	45856662
AMPL7153415157	*ERCC2*	CACACAGGAAACCTGTCACCAT	CATCGTGGCCTTCTTCACCA	chr19	45857878	45858051
AMPL7153415158	*ERCC2*	GGTGCTCTCCATGTACTGGTAG	CAGAAACTCCTAGTTCTAAGACAGAGAAG	chr19	45858008	45858186
AMPL7153414561	*ERCC2*	ACTCAGCCCACCTGACTGATA	CTCGCTTATCGTGACCTCTGT	chr19	45858853	45859017
AMPL7153415130	*ERCC2*	ATCAGGGAGGATACATTCCCATT	CATGGCAACCTTCACCATGAC	chr19	45860309	45860576
AMPL7153415151	*ERCC2*	CTAGCCTCTCCCACTCACCATA	CATCAAACCCGTATTTGAGCGT	chr19	45860509	45860780
AMPL7153415152	*ERCC2*	CAGATGTGATGATGACAGACTGGAA	CCATCATCATCGAGCCCTTTGA	chr19	45860733	45860951
AMPL7153415140	*ERCC2*	CATGCAGCTGGAGAGAGATGAG	GCAGGAGAGGGAATGACTGAGT	chr19	45860794	45861036
AMPL7153415150	*ERCC2*	CCTTTTCTACGCAGACATATTCCAACA	CCCAGCACAGAGGACTTGATTT	chr19	45861979	45862254
AMPL7153415138	*ERCC2*	AGTCTGTGAAAATGTAAATCTGAGCACA	TCACCCTCCTTGCTAACTTTGC	chr19	45864581	45864829
AMPL7153415139	*ERCC2*	TGGCGTAGGTGCTGACAAG	GCTGGTCCTGCTCATGCTTC	chr19	45864784	45864956
AMPL7153415142	*ERCC2*	CTACAGGCGTGGAGGACAC	TGATAGCGTCTCCTCGCAGA	chr19	45866923	45867188
AMPL7153415143	*ERCC2*	CTCAGGAAGCCCAGGAAATGCT	CTCCGCAGGATCAAAGAGACAGAC	chr19	45867117	45867385
AMPL7153415135	*ERCC2*	CGCTGCTCGTCTGTCTCTTT	CTCCATGAGCGTCAACCTCA	chr19	45867353	45867573
AMPL7153415154	*ERCC2*	GAGGTTGACGCTCATGGAGT	TTCGACGAGGCCCACAACATT	chr19	45867554	45867703
AMPL7153415136	*ERCC2*	GATGCAGACGTTGTCTGGAGAA	GCATGCCAATGTGGTGGTTTAT	chr19	45867575	45867800
AMPL7153415137	*ERCC2*	GTCCAGGAGGTAGTGGTAGCT	CTCTATCCATCTGCTCATACTTCTGC	chr19	45867757	45867946
AMPL7153414586	*ERCC2*	GAGATGCAGACAGGCAGACTC	GGTGCCCATACTTCCTTGCTC	chr19	45867979	45868124
AMPL7153415145	*ERCC2*	ACCAGCCTCCTCACTGAGTATC	GCACTCTTGTCCCAGGAATTTG	chr19	45868081	45868227
AMPL7153415146	*ERCC2*	GTCATCCAGGTTGTAGATGCCA	GGAAGGACGTCGATGGGAAATG	chr19	45868152	45868397
AMPL7153415155	*ERCC2*	CTGGGCCACGATGAATGAGAAT	TGAAGAGCTTCGAAAGTTGCTCAA	chr19	45871723	45871996
AMPL7153415156	*ERCC2*	GCCCTCCTGCTTCTCATAGAAG	CACTGTTCCCTAGGCCCTATTG	chr19	45871950	45872155
AMPL7153415132	*ERCC2*	CCAATAGGGCCTAGGGAACAGT	CTGAACCCGTAAAGGCAGACAAAGGA	chr19	45872132	45872315
AMPL7153415133	*ERCC2*	CGGATATGCCTGCCGATAACAA	CTGTTCTGAGTTGGTGGCATTG	chr19	45872241	45872508
AMPL7153415147	*ERCC2*	CGCCCAGCCTATTCACCATTAT	TACTTCCCGTACGACTACATCTACC	chr19	45873224	45873465
AMPL7153415148	*ERCC2*	CTCCCGCATGTAGGAGAACTG	TCCTGAGGACCTGAGGGTTAC	chr19	45873414	45873618
AMPL7153415149	*ERCC2*	CCTCAGTGTCTCCTCGCTATCA	CGGCCTCTCGCTGAATATTCAT	chr19	45873630	45873862
AMPL7153619565	*ERCC3*	AAGAGGTGGAAGGAAAATGTTATGCT	AGGAGTATCTTTTCTTCTGGAGACTAACA	chr2	128015083	128015357
AMPL7153670402	*ERCC3*	AAAAGGGCAACAAGGATTGTTTCTG	TTTCGACAAAAGAAGAGCAACAGCA	chr2	128016706	128016981
AMPL7153619581	*ERCC3*	TGCCAGGACTTTCTGTAAGAGC	TAGGGAGGAGCTCTCCATTGTT	chr2	128016934	128017168
AMPL7153619562	*ERCC3*	GGTATCAAGAAGGCCTTGGTCCTA	GGCATTCTAATGGCTAATGTTGTCTTCT	chr2	128018750	128018979
AMPL7153619560	*ERCC3*	GTATGAGAAATAATGATGGGCCATAAGGA	TTTGATCTGCCGGAAGCAAATG	chr2	128028739	128029014
AMPL7153564461	*ERCC3*	CCATGGGATGAGATCTGAATGAGGA	CCTGGTATAAAGAGGAGAAAAGAGTGATT	chr2	128028967	128029154
AMPL7153564483	*ERCC3*	AGCAGACATGAATTCACTGTCCA	CTGAGCTCCTTTTTCCCTCTGAT	chr2	128030346	128030620
AMPL7153670409	*ERCC3*	AGCCCAAGAAGTTCCTGAGAGA	TGTTTGCCCTAAAGGAATATGCCAT	chr2	128036635	128036785
AMPL7153670410	*ERCC3*	CAAGTTTTCAATTCTTACTTGTTCAGTCGA	TGTGTGGTGTTTAAACTTGTAAAATGACC	chr2	128036730	128037002
AMPL7153670393	*ERCC3*	AGGATCCTGTATGAAGTGGTAGCA	GCGAAGATGACAAAATTGTGGATTTAAATT	chr2	128037889	128038135
AMPL7153670394	*ERCC3*	GTAGAGCTTAGGCCCAATCAGAA	CAGTGTGAATGCTCCCATTGTTTC	chr2	128038082	128038236
AMPL7153670405	*ERCC3*	AACCTAACAACTGGAAATTGGTTTGT	TCAAGATGTGGTCCACCATTGAC	chr2	128044213	128044488
AMPL7153670406	*ERCC3*	CCAGCATGGAGTAGGTGCTAAT	GGTGGGATTGCACTCTTGTTTCT	chr2	128044380	128044621
AMPL7153670384	*ERCC3*	TGGCTTTTCAGCAAGGTGTGAT	GGAATGATTCTGTCAACCCTGATATCAAC	chr2	128046124	128046370
AMPL7153536698	*ERCC3*	CAGCTGTGGGCTTTAGGTCAAT	TTGAGCACAGTAACAAGATTTTAAAACCC	chr2	128046319	128046489
AMPL7153619584	*ERCC3*	GCTTCTAACAGCAGCTCAGTGA	GCTTGATAACAGCAGAATTAAATGTTGTGT	chr2	128046860	128047135
AMPL7153619569	*ERCC3*	TGGGTTAAAGACACCTGAGAGAACT	GCAGTGGAATCTCCAGGAGGTA	chr2	128047188	128047463
AMPL7153564474	*ERCC3*	ACAGAAAAGACAGCATAAACATCAGGT	GGGTTTCCCTAAAGTAAAATGAACTTTTCA	chr2	128047693	128047968
AMPL7153619577	*ERCC3*	GCTGACAACATGGAGCACCTAT	GTGCATGAGTACAAACTAACTGCCTA	chr2	128050108	128050320
AMPL7153670388	*ERCC3*	GCTGACAGCTGCATACAAGGAG	CCTTGTCTGTTTTGTCCATTTGGTATCT	chr2	128050272	128050455
AMPL7153564458	*ERCC3*	GGGCAGTATCCTGAATGTCAGT	GCACCAAAGTGGATGAATATGGAG	chr2	128050988	128051174
AMPL7153670391	*ERCC3*	CATTTGCAGCCTGTAGTCCTTG	GGGCTTACTTCTGTGTTGGTATCT	chr2	128051127	128051323
AMPL7153564475	*ERCC3*	GCTAAGAGCCACCTGCATCC	GTAGCTGCCATGGGCAAAAG	chr2	128051391	128051666
AMPL7153564476	*ERCC3*	GGTCCGCTCGGTCTCTTT	TCGCACGAGCTAACAGATCG	chr2	128051632	128051847
AMPL7153420586	*ERCC4*	CGGCTCTCTTCGGTTGAGTT	GTCAGTGTCGAGCAGTTCCA	chr16	14013936	14014112
AMPL7153420587	*ERCC4*	GTACGAGCGACAGCTGGT	CCCTCCATCCCTGTCAGAGG	chr16	14014072	14014318
AMPL7153420589	*ERCC4*	GTTCATTATCTCAGAGAAAGACAGCACA	TCATAGCGACTGTTGCTTGTGAT	chr16	14015783	14015976
AMPL7153420590	*ERCC4*	CCCTCGCCGTGTAACAAATGAA	TGATGTAGGGAGCTGAGTCCTT	chr16	14015931	14016196
AMPL7153420043	*ERCC4*	GTGGCTATATGCCCAGTCTAGAAT	TCTTGACAAGACTCGATTATTCTGTGG	chr16	14020293	14020463
AMPL7153420603	*ERCC4*	CTCAGGCATCTTGGTGTATAGAGC	ACAGTAGATCTCACAAACAAAGATGACAA	chr16	14020412	14020679
AMPL7153420596	*ERCC4*	TTGCTTTTCGTGTTGTTTGTAGCA	CATGGTAGGTGTCATAGAAACATGGATT	chr16	14021773	14021966
AMPL7153420597	*ERCC4*	ACAGCACAAACCTGAAGTTGTAGA	ACAGCAAATTCCTATTAACAACATTGCAAATAT	chr16	14021914	14022189
AMPL7153420598	*ERCC4*	ACCATTTTAACCATTTTTAGATACACAGGAA	GTTCGTAATATCTTCAAATCCTGAACTAAGGA	chr16	14024451	14024652
AMPL7153420599	*ERCC4*	CCAGCTTGGAGCCAAGACTAAA	GTTGAATATAGCACTTTTCACACCTGATTC	chr16	14024598	14024842
AMPL7153420588	*ERCC4*	GTCATGTGACCATCAGAGACTGT	TTGCACTTTAAATTTCCATACCAAATACCG	chr16	14025938	14026212
AMPL7153420595	*ERCC4*	TTTTAAAAGCCTTTGGAAGACTTTATGGG	GGCTAGTCTTGAACTACTTTTAAAGAGCTA	chr16	14027976	14028251
AMPL7153420583	*ERCC4*	AGGAGGACAAGTGAGGTAATAGTAACAT	GTAGAAGCCCGTTCTTTGTTTTGG	chr16	14028953	14029228
AMPL7153420584	*ERCC4*	TTAGGAAATCTCACAAAAGACCTAAAGACC	CAGCATCCGATGACAAATTTACATCAA	chr16	14029173	14029416
AMPL7153420585	*ERCC4*	CCCGGAAGAAATTAAGCATGAAGAAT	GTGCAACTGAAAGCTGTGATTCTTTATAA	chr16	14029363	14029634
AMPL7153420602	*ERCC4*	GCTCTAGGTTGCTGATTTCACATG	CTATCCTTCCTGAGCAGGACTATCT	chr16	14031530	14031805
AMPL7153420600	*ERCC4*	CCAAGGCACTTTTTACTGACTTTTTCT	ACAACCATGCTTGCTTTTTCCC	chr16	14038372	14038600
AMPL7153420601	*ERCC4*	AGTGTTGACAATGTTTTCTCCCACA	GCAGAGTAACTCTGGAACTTCACAG	chr16	14038553	14038763
AMPL7153420591	*ERCC4*	AGATTTTGTTATATTCCTTCTTTGAGAGTTCT	GAGTGAGGATGTAATCTCCAACCTCTA	chr16	14041405	14041611
AMPL7153420592	*ERCC4*	CATTGACATTGAACCCGTGACTT	AGTTTGGAACTAATGTCATTGCTGGA	chr16	14041561	14041816
AMPL7153420593	*ERCC4*	AGGTGCCTTGTTTCAGGAGATC	GGGACCAGGATTATACTTCTCTGACT	chr16	14041768	14041997
AMPL7153420028	*ERCC4*	CACTGGCCATTACAGCAGATTC	GGCTGCTAATTCTGCGATGTTC	chr16	14041935	14042087
AMPL7153420594	*ERCC4*	GCTCCTTGATGCACCACGTTAA	GAAAAGTACAGGCATGGGATAAGAAAAC	chr16	14042043	14042244
AMPL7153670442	*ERCC5*	GTGCAGTCCGTCGTAGAAGAAT	CCAAGACCATCATCCTGCAGAT	chr13	103498549	103498809
AMPL7153670434	*ERCC5*	ATTAGGAAATTGAAGTTGTGAGGATGAAGA	TTTGCAGAGCCGATGAAACAAAG	chr13	103504398	103504571
AMPL7153670435	*ERCC5*	GGAACTCAATAGAAAATCCTCATCTTCTCA	ATGTGAATTTTTCTAAGAATGCAGAAAGAAAAA	chr13	103504518	103504714
AMPL7153670438	*ERCC5*	CTCTGGAAAATAAATCACAGCAATGTTTCT	GAGAATCGCAGGAAATCAAGACAAAAAT	chr13	103506028	103506298
AMPL7153388425	*ERCC5*	CTCTCGGCTGCATTTATTTTCCAC	AACACATCAAGGTTTAAATGTGTCTGAATG	chr13	103506522	103506797
AMPL7153564428	*ERCC5*	GTTTTATTCACCACTGTAGCCCGTAT	CAGGAACTTTCACAGTTTGATATACCTCA	chr13	103508292	103508562
AMPL7153670432	*ERCC5*	AGTGTATGAAATGTAAATTTCATGGTGCT	GCTGAAGTTCTTAATTCTAAGCATGAATGT	chr13	103510577	103510815
AMPL7153670429	*ERCC5*	AAAAAGCCAATTGTTCTTTGTTCCCT	TATGCTGGTTCAGATAGTTCTTTTTAAGCA	chr13	103513749	103513923
AMPL7153670430	*ERCC5*	TTTCACAGTACCAACTCAAAGGCT	GGTGCTAGTTTATAGAATTCAGAGTCATGA	chr13	103513869	103514143
AMPL7153670468	*ERCC5*	TGTCACACTGTAAAACTGAATGGTGA	CTGCCTTCGATTTTCACTCTCCA	chr13	103514330	103514603
AMPL7153670469	*ERCC5*	CTGGGAAGTAGCTCAGAAGAGGA	CTGTTCTCGGTGGAGCTGTTTA	chr13	103514555	103514782
AMPL7153670470	*ERCC5*	CCAGGAGCAGAAGAAATGCGTA	CGATCTTTTCTGTCCTTAATCATAGACTCA	chr13	103514738	103514998
AMPL7153564435	*ERCC5*	GACTGAAGCCTTTCCGATAAGTGA	ATCATCACTTGAAAGAAGAGAAGAATCGAA	chr13	103514944	103515194
AMPL7153670471	*ERCC5*	AACGAACTTTGCCCATATGAGAGTAAA	CCTCTGAAGGGACACTTACTATGC	chr13	103515137	103515285
AMPL7153670472	*ERCC5*	CCTGTCAGTTTGCAAGAAACAAGTA	TCCAGATTTCTACAAAAGCACAGACA	chr13	103515236	103515484
AMPL7153670422	*ERCC5*	AGAAAGCTCTTGATGATTGCAGGA	AGGTTTGGAAGTTTCAGGGAATTCT	chr13	103517941	103518090
AMPL7153670423	*ERCC5*	TGTGATTAGTGATGAGGAACTTCAAGC	AGAAGTCGAAACACAATGTTACGGT	chr13	103518038	103518291
AMPL7153670461	*ERCC5*	GGAGAAAGAGCTTATTGGTAATTTCAGTCA	TCTGAAGAAGGTGGTAAAGGAAACC	chr13	103518503	103518774
AMPL7153564411	*ERCC5*	GGTCACTGTGTGTCCCTAACTC	CATCAGTGATGGTTCCGGAAGT	chr13	103518854	103519090
AMPL7153670428	*ERCC5*	CATCCTGGACCTGACTGATCAG	ACAAACTCTGAAGGTAAATGTCAGAAAGT	chr13	103519046	103519249
AMPL7153536589	*ERCC5*	TGGTTGGATATAGATATAGATATACACACGTAC	ACTTCCAAGCAAATAAGCCAAATTTATTAACT	chr13	103520351	103520509
AMPL7153564415	*ERCC5*	TGTTACTCTTTAGGATTGGACCGGAATA	AGCATGACAGTTCATGCTGTAAATAATAGT	chr13	103520449	103520697
AMPL7153536612	*ERCC5*	TGTATGAACTATAATGTCTCATTGCTGTGT	GGTGAGTTGCAATGTCCGTAATTTTT	chr13	103524491	103524641
AMPL7153670457	*ERCC5*	CCTAATCCTCATGACACCAAAGTGAAAA	CATATTTGACAAGGTGTTTCCTTCTCTTTT	chr13	103524587	103524794
AMPL7153564420	*ERCC5*	AATCTTAGGAGATACAGGGAATGGAATCA	GAGTTAACTGGTCTTTAACAGCTGTCA	chr13	103525499	103525772
AMPL7153670445	*ERCC5*	ATTTGGTTTAGAAACTTGACTTACTTGTCT	TCTGTTTAGTCTCTGGCTCTTAATACGT	chr13	103527591	103527743

AMPL7153670446	*ERCC5*	GCACAACAGGAGAAAGAAGATGCTAA	TTGTTATGCCTCTCTTCTGGGTTTTT	chr13	103527689	103527876
AMPL7153670447	*ERCC5*	GAATTTGAGCTACTTGATAAGGCAAAAGG	TCACTGAGTTCTGCGAATCTGAAG	chr13	103527821	103528092
AMPL7153670448	*ERCC5*	CTGCGAAAGAGCCAAAAACCAGTG	CGACAAATTCATTACAAATGGCTGTCATAA	chr13	103528044	103528311
AMPL7153652066	*ERCC6*	CAGAAATGCCCGTTAGAAAAAGGG	CCACAGAACACGATGACCTTCTG	chr10	50666809	50667081
AMPL7153652067	*ERCC6*	AGCGATGAAGTTTCTCATCTCCAC	ATCAGTATAGTGGTCTTCTTTTTATAGGATGG	chr10	50667034	50667307
AMPL7153618337	*ERCC6*	AGATGACCATTTTCTTCACATCCAGAA	AAATGTTTCCAGGAAGAAGTTCATTGTTG	chr10	50668320	50668595
AMPL7153618362	*ERCC6*	TCCACACCTGCCCTGATTTTATTT	CATGAAGCACGATGCCATCATG	chr10	50669320	50669586
AMPL7153611172	*ERCC6*	CTGCCTCCACCAGTACATAATCTG	ACTGTACTTATACAGAGGAGAAAAGTGTCT	chr10	50669527	50669681
AMPL7153652069	*ERCC6*	GCTAGAAACAGCCTACTCATTTTCTAATC	GAGACGCCAAGTTTGAAGGAAC	chr10	50678162	50678368
AMPL7153618342	*ERCC6*	CTTCACCAGGTGTGGAATTCGA	GCAAAACTTCTATGCCATCTGGTGA	chr10	50678324	50678599
AMPL7153618343	*ERCC6*	GAAAGACCTAACTTTTCATCAATGCTTTCA	TAAATGATGCCACATCATCTGAAGAGAAA	chr10	50678544	50678818
AMPL7153618344	*ERCC6*	TACTTCAGCTCCTTTAGCCTCAGA	AACAAGTGCAATTTTTGCAGGTATTACAT	chr10	50678765	50679040
AMPL7153618347	*ERCC6*	CTCCAAAGGCTGGTTGAATCCT	ACAAATAGAGTGCTAAAAGACCCAAAACA	chr10	50678869	50679144
AMPL7153618348	*ERCC6*	GATCATTGGATTTGAAAAACCGCCTT	CAGCTGTTATCATCTCTCTCTTATATTGCT	chr10	50679089	50679262
AMPL7153618345	*ERCC6*	CACTTTGGAAAAATTCCCTGCTCAA	TAGCTTTGTTTCTTATAGTCAGTGCTTGT	chr10	50680351	50680591
AMPL7153618363	*ERCC6*	AACCAGAACATCAAAGGACAGTCA	GGCAGAGGCTGAGAACTGTAAC	chr10	50680865	50681140
AMPL7153618335	*ERCC6*	GTAAGGGTGTGGATACGCTTAGT	GGTGTTACCATTTACACAAATAGAGAAGC	chr10	50681442	50681717
AMPL7153611160	*ERCC6*	GATTGAATCCCATTTTGTGAGTTGATGG	GGAAAATGATTGTTGTTGAGTCTTTGTTGA	chr10	50681996	50682155
AMPL7153652068	*ERCC6*	TGACCCTGCTTGTGCCATATTT	GTCATCTGAGTGTACATGTACTCTTTCTTA	chr10	50682103	50682324
AMPL7153618353	*ERCC6*	CTGGCACAATGTAGATCCTAGTTTCT	GCTCTTTTGATCAGGAAGTTTTGTTGATAT	chr10	50684173	50684448
AMPL7153618336	*ERCC6*	CGCAGAGGAGAATCAGAGTGAA	GAATGGAAGCAATTGAGAGGTAAGAAC	chr10	50686322	50686597
AMPL7153618359	*ERCC6*	TGGCCATCTTTCTCACATTCTGAA	CCCTCATCGGATCATTCTGTCTG	chr10	50690626	50690901
AMPL7153611166	*ERCC6*	CTCTCGGAGGTTATTTTGCATCG	GTGTAAATAATTTGCAGAGGAGCGTTTT	chr10	50690849	50691001
AMPL7153652055	*ERCC6*	GGAAAATGCACATTTTAAATTTCTTGTGCA	CTTGGACGAAGGACACAAAATTCG	chr10	50691253	50691452
AMPL7153652056	*ERCC6*	GGTGACAGCAGCATTTGGATTT	AGGGTGTCACTTCTTATTTAAAACTGACTT	chr10	50691406	50691614
AMPL7153618346	*ERCC6*	AAAAACCAAATATGTAAAATGGAGGGCATT	GAGCAGTTGATGACTGTATATGCTCT	chr10	50701096	50701360
AMPL7153652058	*ERCC6*	AAATATTAATTGAGCTCCACAGACTGACA	CCACCATTTGCCATTTTCTCTTTTCTT	chr10	50708534	50708789
AMPL7153652057	*ERCC6*	CAATTTTAATGAACAGCACGTGGCTAT	GTGATGCTGAATTTGACGAAGGTTTTA	chr10	50713756	50713990
AMPL7153618332	*ERCC6*	CTTTTTGAACAGAAAACCTGGCACTT	GAGACCGTTCTAATTTGCCTGTTTTT	chr10	50713937	50714166
AMPL7153652063	*ERCC6*	AATAAACCTGTCACCTAAGATAAAGCAAAC	GATTGATGATGACTTTTTCCCAAGTTCTG	chr10	50732015	50732213
AMPL7153618355	*ERCC6*	TACAGAAGCAGCTTCAGCTTCTTC	GACCTTGGGAGTCAGACATGAG	chr10	50732158	50732418
AMPL7153652064	*ERCC6*	CAGACTCTTCACCCTCAGAGTCT	CTTTTGAAAGGAAGAAGCAAGGTTGTAAT	chr10	50732358	50732631
AMPL7153652065	*ERCC6*	TGGAGCTTTTCTAGCTGCTCTTTT	GCGAAACGCATTGCTATTGTTCT	chr10	50732578	50732851
AMPL7153652070	*ERCC6*	CACTAAGGCAAAGAAACGTATTTTTCTCA	TCACTTCATTGCTGTTTTCTGTCTTCT	chr10	50736339	50736614
AMPL7153652059	*ERCC6*	CCCTAAATGATGACTTTTTCAAAAATGCAA	AAGGGACATGTTTTTAGCATTTTTCTCTTT	chr10	50738712	50738975
AMPL7153652060	*ERCC6*	ATTACCTGAATATCCCTGTCATGTTTTACC	CAGATCCAGGCAGTAGAGCCTA	chr10	50740523	50740776
AMPL7153652061	*ERCC6*	TCATAGACGTCCACACCCAAAC	CCTGTCAGTAATAATGAAGAAATGGCAATC	chr10	50740708	50740947
AMPL7153652062	*ERCC6*	CCATCACCACCACTTTCTTGCTT	CTACAATGAATCCTATATAACGAAAAGGCT	chr10	50740894	50741075
AMPL7153665574	*GEN1*	TCATTTTGAAACGCTGACTGATGAA	GAGACTCAGATCAACTGCAATGGT	chr2	17941051	17941309
AMPL7153665575	*GEN1*	CCTTGCGTAATCTTGGTGGGAAA	AAAGATGGTGAATCTAGACATGTTAAATAGATC	chr2	17941262	17941523
AMPL7153665577	*GEN1*	TGATACTAGTCTAATGTTCTTGCCTTTGTG	TGATCTCCCTGTTTTCTGAGACCA	chr2	17942566	17942825
AMPL7153665578	*GEN1*	CGGTATGGGTCTTCTGGAAAATCG	CACATCCCTGCTAGTCACAATAAAATTAAT	chr2	17942777	17942963
AMPL7153665579	*GEN1*	AGCAGTGAAAACATTCTTTAACATCTTGC	AAAAGGTAGTTCGTAAACAAATGCTGAAAA	chr2	17946115	17946389
AMPL7153212645	*GEN1*	CCATGCCCAGCCTGGAATAATTA	CATCTCTATCCAAACCTAGTTTACTCTTGA	chr2	17947724	17947909
AMPL7153599819	*GEN1*	ACATGTTGACTGTTACACAATGTCATCTA	TGCCGATTATTTTAGTCACTGAATAGCTT	chr2	17947850	17948114
AMPL7153599818	*GEN1*	CTCATTCTTTCAGCTGCTGACTCT	GGAAAACAATTGTTTCCTGTACTATTCGT	chr2	17949905	17950165
AMPL7153599817	*GEN1*	GGTAGGTGAAAGGGAATAGATCAGC	TGGCATGTAAAAAGTACTATGTGAACAAGA	chr2	17952364	17952634
AMPL7153599815	*GEN1*	ACCTTTCTATATGCTAGGTAGTGTGCT	TGCATCCATTACGTTCATGATCCTT	chr2	17953768	17953933
AMPL7153665576	*GEN1*	TGGGTCTCCATAAAGGTTCACCT	TCATGCCAGGAAAAATCATGAGTTTTTAAA	chr2	17953885	17954090
AMPL7153599814	*GEN1*	ATTTCTTATTAAATGGGAGGAGATTGGCT	CCTGATAACCTTCACCAATTTATCCTTGTT	chr2	17954268	17954536
AMPL7153599813	*GEN1*	GCTGTTGTGAGGGATTCCCATT	GACCAATCAGAGTGAATATCTGTTCCTATC	chr2	17954370	17954645
AMPL7153665580	*GEN1*	AATATCACCTAGGAGTGCTATTTGTCTC	TCAAGTAAAAACACCACCTTCACCAT	chr2	17955466	17955711
AMPL7153599810	*GEN1*	CTTTTGAATAAACTAGTCAAAGGAATAGGC	GGAATATTTTGCTTAAAGTGAATACATACCAG	chr2	17959116	17959359
AMPL7153212820	*GEN1*	TTGTTTTGAAATAGAATGGGAAAAGCCTG	CATATATGTACACACACATGTGTGTGT	chr2	17959301	17959575
AMPL7153599809	*GEN1*	CATCAAGCCTGACCGATTGAATT	AGGTGGCTCTTCTATAACATTTGCA	chr2	17961172	17961447
AMPL7153599835	*GEN1*	TTTTTACCCAAGTTAAAGCAAAGAAAATAAATG	GCAAGCTATTCAATGAGGCAGAAATAGAT	chr2	17961825	17962067
AMPL7153599836	*GEN1*	CCCTGATCCTACATTACCACAGGA	CAAGTGTAGATCAGCAATCACGGATATATT	chr2	17962014	17962234
AMPL7153599837	*GEN1*	ATCTAGTCAACCCAATACCTCATCTCAT	AGAATCCTCATCCAACACTTTCTTTATGTT	chr2	17962176	17962432
AMPL7153599838	*GEN1*	TGTGAATCAGAAAGGTACACTGCA	CCTGGAAGATCCTTTGAAAGATGTGA	chr2	17962378	17962653
AMPL7153599839	*GEN1*	ATTGCTAACAGTGGTTCTGATTGTACA	GTCAAGGCAAACACTCTTCTTCATTAAAAT	chr2	17962600	17962873
AMPL7153599840	*GEN1*	AGTTGATATGCAAACCACTCGGAAA	CAGCTTTCTTCATTTTCAGCTGTTTCATA	chr2	17962818	17963076
AMPL7153599841	*GEN1*	ATTAAAGAAACTGAACAGTGTGTCAGATCT	TCTGTCTCTGCTATTGCTGATAGTACTAAA	chr2	17963017	17963267
AMPL7153555077	*LIG3*	TTTTTGGCCTCCTACTCTTTCGT	TCTTAGATGGCTTCCCTGGAATGATA	chr17	33309993	33310219
AMPL7153555078	*LIG3*	TCTTCCTGAGAAGAAAGCCTGTTC	AATGTGGTACCACTCTTTCATATCACC	chr17	33310169	33310444
AMPL7153555079	*LIG3*	GCCCAATCCCTTCTCAGAGTCT	CAGTAAGATGAGAAAGCAGTGTTCTCTAC	chr17	33310392	33310605
AMPL7153555106	*LIG3*	TTTTCAGAGATCGTTCACTGAAGGA	TCTTTAGCCAGAATCCTAGGTAGAATACAA	chr17	33312953	33313227
AMPL7153555096	*LIG3*	AGAAAGGCAGAATGAATTCATCTCTGT	CGGAGGAAAGGCCTATTCAGGA	chr17	33316443	33316718
AMPL7153555093	*LIG3*	TTGCCCAGCCTTCTTTGAGTTTA	TCTGCTTATCGTTCAAGTTGTAAACAGT	chr17	33317905	33318068
AMPL7153555094	*LIG3*	GCTGCTGCCAGGAGTCATTAAG	ACAGTATGTGTCCCAACCAGAAAAA	chr17	33318018	33318235
AMPL7153555061	*LIG3*	TGGGCTAGTGTCTCTAAGAAGTGG	CTTGCTCTGCTCAAAGAAGACTCT	chr17	33318537	33318734
AMPL7153555062	*LIG3*	GGGTGACGTGTCAGAGACAATC	ATCTCTCTATGGGCATGGTTTGAC	chr17	33318688	33318897
AMPL7153555109	*LIG3*	CCGTCAAACCATGCCCATAGAG	GACTCAAAAGTCCTGACACCAGTT	chr17	33318871	33319135
AMPL7153555111	*LIG3*	GTCAAACAAAGGCAGTGGTTTTGT	CAAAGGATGTGGAGCTTGGAGA	chr17	33319492	33319767
AMPL7153555064	*LIG3*	AGTCCCAAGACCCAGGTAACTA	ACATGCCATTGGGACATTTCTTCA	chr17	33321182	33321349
AMPL7153555065	*LIG3*	TGCAAGTCCGTTGAGTATGCAA	TACCACAGCTGGAATGGCTATG	chr17	33321303	33321544
AMPL7153555071	*LIG3*	GCCATGTCATCCCTCACCAAAG	ACTCACACTCATTCATTCTTGCACAT	chr17	33323042	33323258
AMPL7153555069	*LIG3*	CCAGAAGTGGACTGGTACTCCT	GTACCCTCCTTTCCAACCTGAAG	chr17	33323512	33323770
AMPL7153555101	*LIG3*	GGCAAACCCTTTCTTGAAGTAGC	GCAGTTGACCCAAGGAGTTCTC	chr17	33324644	33324918
AMPL7153555083	*LIG3*	GGGATGAGAAGCAACCTGCTTT	GTGAGTCGACAGGAGAAGCCAA	chr17	33325078	33325352
AMPL7153555058	*LIG3*	GTGAACCCATCCTCATGGAGTT	GCAGATCAAGCCAGTAACAAGG	chr17	33325561	33325834
AMPL7153555066	*LIG3*	AGAGGTAAACTCTGGCACTCTGA	CTCACCTTGCTGATCTTCACCAT	chr17	33326207	33326473
AMPL7153551411	*LIG3*	CCGCCTGCAGAATGAACTAGAC	CAAGCTGCAACCCTTCTTTCTG	chr17	33326428	33326585
AMPL7153555055	*LIG3*	ACATCAGCTGTTTAAGTGGCTTCT	GGACTTTCAGCGAGGTGAGATG	chr17	33326695	33326970
AMPL7153555087	*LIG3*	CGGCCACTGATACTATCTTCTTAACC	GGGTGGAGAATACATATACAGCCTAAG	chr17	33328219	33328458
AMPL7153555073	*LIG3*	CCAGACTGGGAAGGCAATGAAA	TGGAGTTACTCAGCTTCCCTTCT	chr17	33328876	33329111
AMPL7153551423	*LIG3*	CCTCAGCCAGTACCAAGAAAGC	GATCTCAGGGCTGGAATGTGAG	chr17	33329066	33329219
AMPL7153555089	*LIG3*	ATCAGCAATCCCTACCCTCTTAGT	ACCTTCATAGCGGAAGGCTTTG	chr17	33329488	33329723
AMPL7153555090	*LIG3*	TCTGTCCTGCAGGCAACATG	CGTGGTGTGTTGCTGTTTTTACC	chr17	33329674	33329836
AMPL7153555099	*LIG3*	CTGCTCACCCTATGTCCTCTTG	ACCACAAACTCAATGCTGCATTC	chr17	33329980	33330188
AMPL7153551443	*LIG3*	CTCATTCTGTCCCGTGCTCATT	AATGCCACAAAGTAGCGTCTGA	chr17	33331205	33331380
AMPL7153555086	*LIG3*	CACACCAGACTTCAGCCGT	AGAGTATGGCCTGAGGGAGAG	chr17	33331338	33331560
AMPL7153195194	*LIG4*	GCAATGAGTCTGCCAGATCAGA	CTGAGGGAGTGTCTCATGTAATAATTGG	chr13	108860832	108861047
AMPL7153195200	*LIG4*	ATCTGCAACACGACTATGATCTTCC	GCCACACCGTTTATTTGGACTC	chr13	108860994	108861173
AMPL7153195207	*LIG4*	TTGGTACTCAGGTCATTAATAACAGCATAC	GCTTTATGATTCATATGTGCCCATCAAC	chr13	108861121	108861395
AMPL7153195213	*LIG4*	TCATATTCACGGGCAAAATGTTCTTTG	GTGGAACAGATAGCCAGCCAAA	chr13	108861340	108861614
AMPL7153195217	*LIG4*	CCAAATTCTGCAATTCTGTTCTCCA	CTGGACGACCTAGAACAACTTAGG	chr13	108861559	108861831
AMPL7153195221	*LIG4*	TGTTTAGATGCGAGCTTACCAGATG	GCCAAGTATTGGAAGCCTTTTCA	chr13	108861775	108862050
AMPL7153195228	*LIG4*	GCTGCTTGGTGGAGCTTTTCTA	GAGTATGTCAGTGGACTAATGGATGAATT	chr13	108862005	108862260
AMPL7153195235	*LIG4*	CCCAATATCCTCCAACAATTAAAATGTCC	GCAGAAAACACAAGCTCATACTAAGAATGA	chr13	108862202	108862408
AMPL7153195241	*LIG4*	TCTATTGCTTCATTCAATGCATCAATTACT	CTTTCATGCAAAAGGGAACTAAGTTTGATA	chr13	108862348	108862595
AMPL7153195246	*LIG4*	GCAGATCAGAATCCTCTACCATTCTTTTAA	GAAACCAAGCTAGATGGTGAACGTAT	chr13	108862535	108862806
AMPL7153775231	*LIG4*	ACATCTCCATCTTTGTGCATTTGC	CATAATGATGCTGCTGAGTTGCATAAT	chr13	108862756	108862995
AMPL7153775232	*LIG4*	ACAGACTTTTTCCAGATCTGTAGTGAC	AGCCAAGATGTTTACAGAAAGGAAGTTT	chr13	108862941	108863216
AMPL7153775233	*LIG4*	AAGGTCGTTTACTTGCTGTATGGTT	CACAGACTCTTTTTATCCAGCAATGAGA	chr13	108863163	108863437
AMPL7153775234	*LIG4*	TCTCTCTTTCTAGCTGAGGAAGAATTAG	TTGCTTTACTAGTTAAACGAGAAGATTCATCA	chr13	108863381	108863656
AMPL7153775201	*MDC1*	TAACGCCCTGAGTTCTTTCTTCC	TCACCTGATTTTGCCTTTGCTCT	chr6	30668170	30668443
AMPL7153775221	*MDC1*	CCCTCCAATCTTTGCCATTCCA	GAGGCTATCTTTTAACCTGTGCTTCA	chr6	30670250	30670524
AMPL7153775220	*MDC1*	AGGGCTGAAGCACAGGTTAAAA	ATTTGTGAGCTCTCTCTAAATGCTAGG	chr6	30670493	30670740
AMPL7153775190	*MDC1*	CTAGGGCACTATATGAGCAGTCTTG	TAACCACCTCAGGTCAACCCTT	chr6	30670817	30671091
AMPL7153775188	*MDC1*	CACCCATCTCTACAATCCTCTAGGT	TTGGGACCACAAAGCTGGGAAA	chr6	30671110	30671385
AMPL7153775202	*MDC1*	CTGAAGTCATTTTTCCCAGCTTTGT	TTCTTGAGACACCAATTCATGCCT	chr6	30671352	30671627
AMPL7153775203	*MDC1*	TGCTGGTTCCACCTTTTGGATC	TCACCACAGACCAGCCTATTTC	chr6	30671577	30671849
AMPL7153775204	*MDC1*	CAACTGGGTTGAGTAATAGGCTCA	CTAGGGCCACTAGGAGAAAGACAAA	chr6	30671800	30672056
AMPL7153775205	*MDC1*	GGGAGTCTTGACAGAGGACCTA	GGACAAATAGGTCCTCTGTCAAGAC	chr6	30672009	30672284
AMPL7153774947	*MDC1*	GGGCTATAGGGACAATTGATTCAGG	AAAAATCGGTCCTCTGTCAAGAC	chr6	30672233	30672405
AMPL7153775207	*MDC1*	GGTCTTGACAGAGGACCGATTTT	AGCCTACGTCTCAGGCTACTAG	chr6	30672381	30672557
AMPL7153775208	*MDC1*	TCTTGACAGAGGATCTATCTGTTCTTCC	ACTCCTGAGCCCACATCTCA	chr6	30672506	30672687
AMPL7153775209	*MDC1*	CTTCACAGAGGACCTATTTGTCCTG	GGACAAATATGTCCTCTGTGAAGAACC	chr6	30672630	30672899
AMPL7153775210	*MDC1*	GGGCTATAGGGACAGTTGATTCAG	CCACATCTCGGACCACTAGGA	chr6	30672848	30673046
AMPL7153775211	*MDC1*	GTTGGTCTGTGGAGGTGGAAAT	GACCAGCCTGTCACTTCTGAG	chr6	30672938	30673191
AMPL7153774954	*MDC1*	TCAGAAGTGACAGGCTGGTCT	CGACCGACCTGTCACCTCT	chr6	30673171	30673315
AMPL7153775213	*MDC1*	TAGTAGCCTGAGAGGTGGGTTC	CACATCTCAGGCCACTAGGAG	chr6	30673274	30673537
AMPL7153775214	*MDC1*	CTTGACAGAGGACCTATTTGTCCTG	CCCAAAGCCTAAAATTAGAACTCGGAA	chr6	30673491	30673654
AMPL7153774958	*MDC1*	CTGGAAAGGGTGTCATTCTGGA	TTTACCTTCTATCAAGCCAACCGTT	chr6	30673601	30673750
AMPL7153775215	*MDC1*	CTCCCATCTTGCCTGGTCTTAC	CAGTATCCAAGTTCTATCTCTTTTCTCCAC	chr6	30673702	30673939
AMPL7153775189	*MDC1*	AGGAATAGATTAAAGTGAGGCTAGGTGAA	GTGATACACAGGCCTCGTGATA	chr6	30675069	30675315
AMPL7153775194	*MDC1*	GTTGATTATCACGAGGCCTGTGTA	GTTAGAAGAGAAGGTGCCCAAAGT	chr6	30675287	30675560
AMPL7153775195	*MDC1*	CTCTCTGTGTATCTCTCTCCAGGAT	AAGTCCTGAAAGAGATAGGGAGAGTT	chr6	30675510	30675713
AMPL7153775196	*MDC1*	TCCTCAGATGTCTCAATTTCTACCTTCA	GACTGTGGATAAAGTCATGGGTATACC	chr6	30675659	30675932
AMPL7153775197	*MDC1*	CCACCCTCTCTGCTGTTTCTTTT	CCCTTCCAGGTACCCTAGATG	chr6	30675882	30676143
AMPL7153775186	*MDC1*	CTGGGAAGCTGAAGGAAGAGAAAC	GGAGGAGATGAACTTAGGCCATCT	chr6	30679043	30679317
AMPL7153775230	*MDC1*	TCCTACCCTACCCATACTAGCCT	AAATCAACCAGGGTTCTAACAGCT	chr6	30679345	30679619
AMPL7153775222	*MDC1*	CAGCTGTTAGAACCCTGGTTGA	GCAGGACCTCCCTATCTCAAGAG	chr6	30679594	30679814
AMPL7153775223	*MDC1*	ACCACCAGATCTGTGAGGTTCT	CTGCTTGTGGTATCTCTAGAGGAAG	chr6	30679769	30680035

AMPL7153775224	*MDC1*	TCCTCTGCATCTGTTTCACAGTC	CAGTGATGACAGTGTGGAAGCA	chr6	30679970	30680237
AMPL7153775225	*MDC1*	GGATCCCAGGTGAGCTCTTATC	GGAAGAGCTCCCAGTGGAAAATAG	chr6	30680193	30680342
AMPL7153775226	*MDC1*	GTGATCCTTGAGGACAGCTTCT	CTTCCATGGTTATCAACAGCGATAC	chr6	30680296	30680532
AMPL7153775227	*MDC1*	GCTGAGACTTCTTCCTCGTCATCT	CTTCATCGACAGCGACACTGAT	chr6	30680483	30680744
AMPL7153775228	*MDC1*	GTTGCTGGGATCCTCTCTTCTTC	TGAAGTTGTAACTGAAATCCAGCTTGA	chr6	30680696	30680969
AMPL7153775229	*MDC1*	CTCCTTCACTAAAGGCTGATCCTTT	TCCCTTCCAGGGAAATAAATTCTATAGACT	chr6	30680917	30681181
AMPL7153775187	*MDC1*	CTCCATAAAAATTCAGCTGAGTGAATGA	TGAGGAGACAGGAATAATGAGTGTACA	chr6	30681278	30681553
AMPL7153775192	*MDC1*	TCTCCTCATTCTCCCTGCCAATA	GGAGCCTTAATGGTACTCAAATCCTG	chr6	30681545	30681819
AMPL7153775193	*MDC1*	GGCTCAAAACCTTAGGAGGTCT	CAGGACTCTGATCTTGGAGCTT	chr6	30681771	30682003
AMPL7153775191	*MDC1*	AACCCTCCAAATACCTTACCACAAAA	TGATAACTCTGATGCTGTTTGTGTGA	chr6	30682746	30683021
AMPL7153127229	*MRE11A*	ATGGAGTTATGCTCAGGAAACAATACTTAA	CCATGTGAAATGACTCTCACTGGTAT	chr11	94153180	94153453
AMPL7153126988	*MRE11A*	AGCAACTAGCTGGCAGTCTCTA	GCTGGATTGAAAAACTTAGCCAGAAAG	chr11	94162999	94163237
AMPL7153307880	*MRE11A*	TAGCCCTTGGTCTGTTTTCATTCC	TCAGAGTCTATTCTCACAAAAACTGTTTGT	chr11	94168843	94169114
AMPL7153307899	*MRE11A*	TCAGAGAGTTGACAATTCATAATGCAGAA	GCATCATTCAGTTTTGCAGATAGGACT	chr11	94170270	94170515
AMPL7153126844	*MRE11A*	CTACCAATGGTGATTACCTTGGTCT	CTGGCTTTTTACAGACAAACTATTTGACAT	chr11	94178904	94179179
AMPL7153126799	*MRE11A*	TCTGTATTTTCCACTCAACTGCCAA	GCTAATGACTCTGATGATAGCATCTCAG	chr11	94180324	94180505
AMPL7153307885	*MRE11A*	GCCTCTTCCTTTGTTGGTTGCT	TCATTGTGTGTACTTTCTCCTTCTTCTC	chr11	94180454	94180640
AMPL7153307874	*MRE11A*	TGTAGGCTGAACTGAAATCAACCAA	TACATAACTGGAATAGGCAACATGTTTGT	chr11	94189280	94189555
AMPL7153307877	*MRE11A*	AATCTCAGAGCTACTCTTAAAGACAGACT	AACTATTTCTGCATCCAGTGATTTGTATCT	chr11	94192507	94192782
AMPL7153127202	*MRE11A*	AGGTAGCCATTATTCAAAATGTGAACTGTA	ACTTGCAATGAGTATGCACATTTTAACATT	chr11	94194062	94194272
AMPL7153127067	*MRE11A*	GAAGACAAGAGAATTATTCCCACTGTCA	AGTGTTCTTCGCTTTAGCCAGAAAT	chr11	94197127	94197375
AMPL7153127092	*MRE11A*	TGGATTAGCTACCCGATCCACAA	TGTGAATACTGAAGGATTAGTGTGTGTTTT	chr11	94197327	94197489
AMPL7153127281	*MRE11A*	CACTCTCCTCACTACTTTTCAAAGAAAC	ATTATGTAGTTCAGGAAAATTCCTGTGGT	chr11	94200884	94201159
AMPL7153307901	*MRE11A*	CACATTCACTGAAGAACAGTGTCCT	GCAGTTTTTCATGGAGGATATTGTTCT	chr11	94203489	94203739
AMPL7153307902	*MRE11A*	TGGGTTAAAAATGTCTGGATGATTAGCT	GACTCGGTGTTCATTTCTCTCCA	chr11	94203684	94203837
AMPL7153127025	*MRE11A*	TTAACATAGGCCTTAAACCTATGAGATGA	CTCCAACCAAAAATGAACAACAGCT	chr11	94204655	94204830
AMPL7153307900	*MRE11A*	TGAGCTTCCAGGTTGTGAGATATAAAAC	ACTTTCTGGTACATAGAAGGATGCAATAAA	chr11	94204777	94205051
AMPL7153307890	*MRE11A*	CCTCAGCACTTGGCTCAAACTT	CCAACAGTAAAGTGAAATACTTTGAGAAGG	chr11	94209413	94209680
AMPL7153126907	*MRE11A*	CTCAATTGTTTAAAGAATCACACATGGACA	CATGCAGATACCTACTTTTACATACACACT	chr11	94211844	94212099
AMPL7153127256	*MRE11A*	CTACACAATGAGAAAAAGGGAAGGCAT	TGCCTATGATTGCATTAATATTTTGGAGGA	chr11	94212744	94213019
AMPL7153307897	*MRE11A*	GGAAGGCAAAACAGTTGTGTGT	TCAGGTGGATTTTATTTTGTTAGGTGGT	chr11	94219000	94219254
AMPL7153307898	*MRE11A*	CCTTGAGGGCTTATTTTCATGAAAAAGATC	ACCAGATTGAAAGTCCCTTTGAATAAATAA	chr11	94219196	94219409
AMPL7153307883	*MRE11A*	GGAAAAATAACTTGTTAACCAAGGGAATATGG	AAGTTTGTCTCAATTTGTTTGAATATCCTTTAA	chr11	94223935	94224207
AMPL7153126930	*MRE11A*	CTGTTCATAAACAGGTTCCTTATTTACTGC	GTGCATTTTCTGACATTTGAGTACAGT	chr11	94225828	94226050
AMPL7153775180	*MUS81*	GTTCGAATCCCGACTCCAGAA	ACCCACCTTCTGAAATACGAAGC	chr11	65628147	65628365
AMPL7153775181	*MUS81*	TTCGCTGGCTGACCGAGTG	GGGTAGGGTACCTGGTCAG	chr11	65628288	65628431
AMPL7153775183	*MUS81*	CCCGATGGGAAAAGCTGCT	AGGGCTCCCAGGAGAGTAC	chr11	65628375	65628647
AMPL7153775172	*MUS81*	TCTGAGTAGGTTGCATCCCAGAT	CATCACTGGTGAGATTTGGACTCA	chr11	65628743	65629010
AMPL7153775175	*MUS81*	TCAAAGATGACTGGCTGGCTTT	GTGGCTGGGATAATCTTTGCCT	chr11	65629366	65629618
AMPL7153774907	*MUS81*	CACTGAGCCTACCCTGTCTTTTC	GCAAGAAAAGGCAGTCTTTGGTG	chr11	65629644	65629819
AMPL7153775176	*MUS81*	CCCACCAAAGACTGCCTTTTCTT	GGGTGACTCTAAGGCCCTACTA	chr11	65629794	65630067
AMPL7153775170	*MUS81*	AGGCCCAAGGAACTGCTCTA	GCCCACATTCAGCAAGCTCA	chr11	65630362	65630614
AMPL7153775171	*MUS81*	AGAAGTTGGCCGAGTCAGAAG	ACAGTTCAGCTGCAGGTGAT	chr11	65630570	65630772
AMPL7153774888	*MUS81*	GGTTCTGGCCTCACATACCAAC	CTCTGATCATCTCCCTCGTTTCTC	chr11	65630845	65631011
AMPL7153775185	*MUS81*	CTCTGATCTAGGCTTCCCTCCTT	CCACAATGTGATCCAGTACCAACTC	chr11	65631037	65631310
AMPL7153775169	*MUS81*	CCAATCCTAGAGACCCAGGTGAA	GGATTGCTCTGGGCTGAAAAC	chr11	65631174	65631440
AMPL7153775184	*MUS81*	CACCATTTTGAGTGTGACATCATGG	CTGGCCTAGACCATGAACCATG	chr11	65631882	65632155
AMPL7153775173	*MUS81*	GTGGTCCATGGTTCATGGTCTA	CTCACCCACCTTGCTTCTTTTG	chr11	65632127	65632383
AMPL7153775177	*MUS81*	AAAAGAAGCAAGGTGGGTGAGAT	AAGCTAGGCAGAGACAGTACCTTAT	chr11	65632362	65632636
AMPL7153775178	*MUS81*	TTCAACGCAGGAGCCATCAAGA	GGATTAAGGTGAATTCCATGCATGGA	chr11	65632589	65632861
AMPL7153775179	*MUS81*	CCACTGTCAGCCTCAGAAACTC	CAGTAGAGCTGGGATAAGGTCCT	chr11	65633242	65633501
AMPL7153775174	*MUS81*	CTACAGAGGTGAGGGCAAGAGA	GCTGCAAACACTTAGATTCTAATTGTACC	chr11	65633357	65633630
AMPL7153741664	*NLRP2*	GCAGGAACTGGCATTTGAGACA	GGTGGTGAGGATTTCTACCAGTTG	chr19	55481294	55481566
AMPL7153741665	*NLRP2*	ACAAGGAGGTAGACAAGGCTGAT	GGACACAGTGTGGACCGATTTC	chr19	55481513	55481692
AMPL7153741647	*NLRP2*	GGAGATAAGAGAAAGGAACAAGTGATCC	CTAAGTCCGGCATTTCTTTGCA	chr19	55485749	55486024
AMPL7153741654	*NLRP2*	ACGTCCTTTTTAGTACCTAATCTAGGCT	GGGTTCTTCAAAGGAACAGGAGA	chr19	55488999	55489274
AMPL7153741646	*NLRP2*	GCCTCCTTTTCTCTAATTGGGACT	GTGTTACATAGGAAAGTTAAGAGGACTTCA	chr19	55492905	55493135
AMPL7153741549	*NLRP2*	TTTTGGTTTTCCCTATGGGTAACTGA	TGTATCTCTCAGCCATAACCTGGA	chr19	55493464	55493634
AMPL7153741635	*NLRP2*	CTGGCCTGGAGATAGCAAAGAG	AGCTGAGGTAGAACGCATATTTGAAT	chr19	55493587	55493805
AMPL7153741636	*NLRP2*	CAGAGGACAACCTCATCCACAA	CATCAAAGCCGTCAATCACGAAC	chr19	55493757	55493940
AMPL7153741637	*NLRP2*	TAGCCCAAGCACGGAAAATCTT	AGGATCCGGAGGTCCCTCA	chr19	55493895	55494106
AMPL7153741638	*NLRP2*	TGAACAGGGTGATGTTACCCAAG	TTCAGAGTCGTGCACACGAT	chr19	55494030	55494292
AMPL7153741639	*NLRP2*	CTTTGAGCTAATGAGGAGCAACG	CTTTCCAGATCCTCTCGGTGAA	chr19	55494208	55494481
AMPL7153741641	*NLRP2*	GCTTCACCGAGAGGATCTGGA	GTAGCCTGCTTGGATCAGGTC	chr19	55494457	55494731
AMPL7153741642	*NLRP2*	TCCGGAGTAGAAAGACTCAGGAA	CGTTGAATGTCCACCCTTACAAC	chr19	55494683	55494860
AMPL7153741643	*NLRP2*	GAATTGCTGCGATGCGACATAA	GCTTGACGCAGAATGAAGATGG	chr19	55494815	55495002
AMPL7153741644	*NLRP2*	GCACTTAAATGCAGTAGACGTTGTG	AGATGGAACGACTGGTAGAGTGAA	chr19	55494955	55495131
AMPL7153741648	*NLRP2*	GATGGACACACATTTGGTGTAACC	GTACCATTTTCATACCCTAAGGATTTGAGT	chr19	55496370	55496637
AMPL7153741652	*NLRP2*	TAAGTACGATACAGGTGTACACACTAAAGA	CTTGTGGTAGCTTATGTTTGTATGATGAAG	chr19	55497462	55497737
AMPL7153741649	*NLRP2*	AAAAATGACGTGGTCCTATTTCTCC	GCATGACCATTGCTCGTCTGT	chr19	55501359	55501629
AMPL7153741650	*NLRP2*	GTCAACCGTGTTGCCATTTGT	GGGCGTGTGCATATACACC	chr19	55501835	55502089
AMPL7153741653	*NLRP2*	GAGAACTCACAGGTTCGGGTTT	GCCACAGATTCAGTAAGGTGTAAGTATAG	chr19	55505588	55505862
AMPL7153741634	*NLRP2*	CGATCAGCACTGGCTGCATTAA	CCTAAACCTACCACTCTGGAGAAAG	chr19	55508637	55508907
AMPL7153741878	*NLRP2*	CTTAACAGACTTTCAGGTACTTGGGAA	CACCTGGAAAATCGATGACTTCATG	chr19	55512042	55512314
AMPL7153195966	*NHEJ1*	AGGCCTCTGACTGTATCACTATTTTAGA	GGAGCCATGAGAGGAAGAAAGTT	chr2	219940988	219941263
AMPL7153147908	*NHEJ1*	GCCAATGAAGCAGCAATTCCAG	CTAATATCTTCCTGGTAAGAAGAAGGCA	chr2	219941876	219942150
AMPL7153148159	*NHEJ1*	ATGTGGCTAGAAAACTATCATATTGGACAG	GCTTCTCTAGCAATACAGTGTAACCA	chr2	219942722	219942980
AMPL7153148104	*NHEJ1*	TTCCAACCACACAAGCCACCTA	GCACCTTCCTCCTCTTTCCTAAGTA	chr2	220011296	220011568
AMPL7153775168	*NHEJ1*	GGGAGGCACTTCTCTAATGCAA	GTCTTTTGGCTAATTGATATCTCTTCCCT	chr2	220012278	220012551
AMPL7153148018	*NHEJ1*	AAATAAAAGCACCTAAAGCTTCCTCTCA	GCAGCTTTCCTCTGTCATTTGGAT	chr2	220022118	220022376
AMPL7153148055	*NHEJ1*	TCAACAATGGGCGAAGGAGATT	TTTCCTTTGCTGTTACTTATGAGGGAA	chr2	220022330	220022481
AMPL7153775167	*NHEJ1*	GCAAGCTGGTTGATGTTCACAT	GCATGACCCTGAGGCTCTTTAC	chr2	220022852	220023127
AMPL7153241492	*PARP1*	TGTAAAATCCTTTCTGAGGTGGTTTAGTAC	GGCACATGTACATACCCTCTGT	chr1	226549026	226549301
AMPL7153241452	*PARP1*	AGGTAATCTGACAGCACATAAGTGAC	TCAAATAGAGCTCATCTCCCTGGAA	chr1	226549588	226549863
AMPL7153241477	*PARP1*	CGAAAAGATAAAATGAATCTCCAGCTGTT	AGTGTGAAGGCCTCTTCTGAGA	chr1	226550668	226550901
AMPL7153241443	*PARP1*	GGGAGTTCTGCCTATAGCCCAT	ACTCAGTGAACAGCTGCTCCTA	chr1	226551571	226551839
AMPL7153241486	*PARP1*	TAGACCTCTTGCTCAAAGTTCTTTATGG	ATGGCTTCTTTTGTCCTTCCCTT	chr1	226552622	226552897
AMPL7153241541	*PARP1*	GAACAAATGAGTCACTTCTTTGGGTTATT	CCTGGGTCTCTTTTGTGCTAATCC	chr1	226553521	226553796
AMPL7153241439	*PARP1*	CCTAACACACATGGAAGTTTGGGA	ACCTGGGTGAGTCTGTCTCATT	chr1	226555101	226555373
AMPL7153241521	*PARP1*	AAAACAGCTCTGCCTCCATCAA	GATCTCTCAAATCGCTTTTACACCCT	chr1	226555725	226555986
AMPL7153241524	*PARP1*	GGCTTCTTCATCCCAAAGTCGT	CACATACCACGCTACTTCTGGA	chr1	226555930	226556139
AMPL7153213427	*PARP1*	AAAAATCGAAACCCTCGTGAAGT	CGCATACTCCATCCTCAGTGA	chr1	226557991	226558165
AMPL7153213433	*PARP1*	TGGTAGCCCTGTGCTTACCT	CCTGCGATCACACTGCACA	chr1	226558116	226558275
AMPL7153241511	*PARP1*	CAAGAACAGGCAGACAGCTCTT	CCTTGCTTGAGTTGTTGATTTGGG	chr1	226561840	226562108
AMPL7153241459	*PARP1*	GAAGCAGACAGTGTAAGGGCAT	ACCCACACATGTGTATCTGACAC	chr1	226564775	226565044
AMPL7153308437	*PARP1*	TGATGGCACAGCACTAACCAAT	AGAAAACTGTAGGTGCTTTTCCTCTG	chr1	226566769	226567007
AMPL7153241498	*PARP1*	GGCATTTGGATGTGTGCTCCTA	AGAACAACTCATTTGCCTTCTACCTT	chr1	226567193	226567426
AMPL7153241532	*PARP1*	CCACCAATGTCCCTGGCTTT	CCGAGTTGTGTCTGAGGACTTC	chr1	226567570	226567813
AMPL7153235208	*PARP1*	TTGGTGGAGGCGGAGACGT	AGTTTCAGTTTTGTTTTAAACTTGTATTTTGGT	chr1	226567765	226567922
AMPL7153241447	*PARP1*	CTGTGTTCGACTCTTCCTCACT	GCTGGCCTTGCTGAATGATTCT	chr1	226568679	226568946
AMPL7153241504	*PARP1*	ACAATTCCTGCAAAGCAGCTCTA	AGCCAAATCCTCTTTGGTCTATGG	chr1	226570689	226570962
AMPL7153241431	*PARP1*	AAACAGGATGACGCAGCTGTAA	GAGCGATGCCTATTACTGCACT	chr1	226573068	226573301
AMPL7153241432	*PARP1*	CTGTGTCTTGACCATACACTTGGT	CCATCCCACATTAGTAAGTGTCCTTTTT	chr1	226573237	226573455
AMPL7153241508	*PARP1*	CATTGGGACAGTCACTCCACAA	GCTCCTCCTCCAGTTGGATTCT	chr1	226573953	226574211
AMPL7153241430	*PARP1*	ACAACTCCTTGAATTGTCTTCCCA	ATTAAGATACTGCATCACCGCAAGT	chr1	226576271	226576540
AMPL7153235177	*PARP1*	CGAAGGGAAACAGAGGAGTGGTA	TACAGAGGATAAAGAAGCCCTGAAGAA	chr1	226578010	226578164
AMPL7153308436	*PARP1*	CCACCCTTCACTCTTGACTCCT	CCTTGGTACCACATATGGTTTTTCC	chr1	226578106	226578355
AMPL7153241441	*PARP1*	CATCATTGCAAGCAATCCATAAAGTTCA	GGTGTCCTAGTGGAAGGATCCT	chr1	226579846	226580120
AMPL7153235189	*PARP1*	AACATGGGCACCATACGCT	GTCAAGAAGACAGCGGAAGCTG	chr1	226589775	226589948
AMPL7153308438	*PARP1*	CCAGTATGTACACACCTGTCACT	TTACTTACTTCCCTCTGTTTCTCTTCCT	chr1	226589899	226590113
AMPL7153241467	*PARP1*	GCCTTCCCGGACACAGTTAA	GCAGCGTGTTTCTAGGTCGT	chr1	226595443	226595690
AMPL7153775149	*PARP2*	CCAGAACACATAGCGACATGCA	CAGTAGGTGGCATCGTTCCTTT	chr14	20811642	20811916
AMPL7153775153	*PARP2*	TTTAAAATCTACACGTATTCGGGTGTGTA	CATCTTGCTTGTCTTCTGTCCTGT	chr14	20813030	20813246
AMPL7153775154	*PARP2*	TGGCTGGAGGAAAAGCTAATAAGG	CCTGAAAGAAGGACGAAATAGGAGT	chr14	20813198	20813347
AMPL7153775161	*PARP2*	CGGGCAAAGTTAATCCTGACAAG	AGGATTCCTAAAATGAGTACTAACAGCATC	chr14	20813464	20813729
AMPL7153775150	*PARP2*	GAGATGAATGTTGATGTTGCTGTAGAATG	AAAAAGGGACTTTAAAGATCTGAACACTG	chr14	20814899	20815174
AMPL7153775152	*PARP2*	GTGACGAAGGATGAGTCATTGGT	CCAATTTAAACCCAGAGAATCCTTTGG	chr14	20818603	20818873
AMPL7153775164	*PARP2*	AGACTATGGCAGTTAGCAAGTAACTTG	TTTTTGTAGTCACTTTTCAGCACTCA	chr14	20819035	20819308
AMPL7153775151	*PARP2*	TCTTTGGAGATTTTCTGTCTTGTAAGTCC	ACAGAGCACAGTTTTGCCTCTAAA	chr14	20820320	20820584
AMPL7153775162	*PARP2*	AGGAATCTAGAGATGATTTCTTAGATTGTCT	GGAGCTAGGAATTATAAGGAGAGAATGC	chr14	20822186	20822456
AMPL7153775155	*PARP2*	GTACAGTTCACTAGATGGGATCCTAGT	GATTATAGCTGCTGCTGATGGATACA	chr14	20822910	20823183
AMPL7153775160	*PARP2*	ACCTGTTTCTCATACCTGTTTTCTAAAGG	CAATGTCTCCCAAAGCCTGAGA	chr14	20823796	20824071
AMPL7153775163	*PARP2*	CCTTATACCAGTGTCCTCCCACAT	AGGTGGTTAAGGGTGCAAGAATATG	chr14	20824007	20824239
AMPL7153775156	*PARP2*	TCTCTTGTAGAGTCTACATCAGCCTT	CCTCTTATAAGTTCTACAGCTGTACTTTCG	chr14	20824420	20824663
AMPL7153775158	*PARP2*	ACAGCTGTAGAACTTATAAGAGGGAGT	AAAAAGCACTGAGAACATTACTGTATCC	chr14	20824640	20824915
AMPL7153775157	*PARP2*	AAAAACAGGGTCAGTGGTATGCA	CCCAGGTAAACCTCTTGGTTCTG	chr14	20825128	20825403
AMPL7153775166	*PARP2*	GGGATTTTCTGTTTGGCTTTGGA	CAGAAGCTGCAATAGAGAACGTAGT	chr14	20825479	20825754
AMPL7153775159	*PARP2*	CTATTGCAGCTTCTGAACCAGAGA	CAACACTGCTTATTTTCTTGCTTGAAGA	chr14	20825739	20826014
AMPL7153241342	*PRKDC*	TTCTCTTTAGTGTTTCAGGAAAATCAGCT	GAGAGTGGGCTTTCAGAAGAGAC	chr8	48686627	48686838
AMPL7153084240	*PRKDC*	GTCCATCAGGCACTTCACTTGA	CATGCTTTGTGTTTTCTGTGTTTGTTTTT	chr8	48686793	48686974
AMPL7153084222	*PRKDC*	GTTGGCTCCTCGAGAAACAGTAG	AATCTGTTTGGGCATGATTGTATATACCA	chr8	48689337	48689611
AMPL7153084213	*PRKDC*	CACTGTCACAAAAGTGAAAAGTGCA	GTTCTGGCTTACATATTCTGCTCCTT	chr8	48690204	48690479
AMPL7153084197	*PRKDC*	CCTTAAAAACTGTCTATTTCTGGAGGTTGT	GGATTGGAGACAGACATCTGAACAAC	chr8	48690931	48691117
AMPL7153137768	*PRKDC*	CAGTCTCCATGGCCACCATAAA	CCTCACCCTTAGTGGGATCACA	chr8	48691069	48691272

AMPL7153084189	*PRKDC*	GCCTACAAAAGAGACACAGCTGT	CCTCAGAGAGATCATCAAGATTATTCGAT	chr8	48691219	48691482
AMPL7153084181	*PRKDC*	GATGATCTCTCTGAGGCAAAGCT	CAATGAATTCTTGGTGATCTTTGCCT	chr8	48691466	48691739
AMPL7153084173	*PRKDC*	GCACTCCATTTGGTTTATTGACCCA	TGGGTTTACTCGTTAACCTTTTATTCTCC	chr8	48694576	48694851
AMPL7153084159	*PRKDC*	AGAAGGTCCTTCAAGGTAACAGTATTTTC	AGCTCTTCCAGGTCATGAATGG	chr8	48694764	48695038
AMPL7153084165	*PRKDC*	GGAGTCTTGGGCCAGGATC	TTTGACACGTGGCTAGCCT	chr8	48694997	48695209
AMPL7153084150	*PRKDC*	GGCCGTTGTCTCATATACTAAAGGC	GGCTTTTTAAAAAGTAGATCAAGAGGCAT	chr8	48696209	48696461
AMPL7153044116	*PRKDC*	CTGTCAATATCCCAGTATCACTATTCCAG	TGAGCGACTTCAAAGTGGAGTTC	chr8	48697573	48697721
AMPL7153084141	*PRKDC*	GGGAATCTCCAGCTCATTTCTCAG	TCCAAAGTCCAAACTAAACATCAGTCTTTA	chr8	48697674	48697931
AMPL7153084124	*PRKDC*	GTAACTATTGACTGTCACAACGACTCT	GTTGGGTAAAACTTAATTGCAATCTAGCTT	chr8	48701406	48701673
AMPL7153084115	*PRKDC*	GCTAGTTCAGCTCTTACATCATTGCT	ATTGATTTTTGGAAGATATTTGATTGCCTAAA	chr8	48701578	48701853
AMPL7153084099	*PRKDC*	TAGTGTCCACGTGTGTGCTTTAA	CTTCATCATAAGCAGCGAAAGCTATT	chr8	48706769	48706925
AMPL7153299497	*PRKDC*	TCTTATGACCAGTAGAAGTATCCTTGAAGG	CTCAGCATTAAGTTTCAGCTCTCTGT	chr8	48706869	48707093
AMPL7153084090	*PRKDC*	CTGTAAAGTTCCTTACCGCCAAGTA	GGGATACATTGACCATTGCTTTCTTCT	chr8	48710756	48711001
AMPL7153084080	*PRKDC*	AAGACTTTATGCTGACATAATGGAACACT	GCTTCAGTCATGCTCATGGGTT	chr8	48711719	48711993
AMPL7153084072	*PRKDC*	CACTAGCGTAAAACATTCCTCCAGT	CCACACACACATACAAAATTTTGTTTTGTT	chr8	48713323	48713597
AMPL7153084055	*PRKDC*	TGAAATTACTGCAAAGATTTACCTCCCAA	CTGAAGGAGCTGCATAAAGAGTCA	chr8	48715744	48716014
AMPL7153044056	*PRKDC*	CCAGCCAATCGTCTCTGGTTTT	GAAGCAGGCCAATTAGAAACTTCTG	chr8	48715968	48716117
AMPL7153044039	*PRKDC*	GAAAAGCTAATTTAGCAAAATCCTGTGACA	ATGGAAGTGCAAGAGCAGGAAGAAGA	chr8	48719638	48719793
AMPL7153299689	*PRKDC*	AACTCCTGATCAGGGAGCTGATA	GAGTCTGTGACATTGTCTCATTTTTCTTTG	chr8	48719744	48719922
AMPL7153084035	*PRKDC*	AAGGATGGAAAACAGCTGTGCTA	GAGCTGTGTGTATAGTCACCCTCTA	chr8	48729886	48730160
AMPL7153084023	*PRKDC*	CATTAAGAAACATGCACTTTCACTAAGCA	GTCACAGCTTGATTGATTTATAACATGGC	chr8	48731863	48732138
AMPL7153044012	*PRKDC*	AGTATTACCTGCATAAAACTCTGAATGC	GACCAGTCCCTGCTGACATTTA	chr8	48733271	48733444
AMPL7153044017	*PRKDC*	CCCGTGCATAGCTTTGTCAA	CATACTCAAATTGGCCTTCTGTAGTTACA	chr8	48733402	48733555
AMPL7153298983	*PRKDC*	GACTCAGGTCATGCAATTCCCT	TGAGTGGAAATCACTTGAATACTGTTCTAC	chr8	48734034	48734258
AMPL7153083994	*PRKDC*	GGGTTCTCACTGTCTATACTGGCT	GTTCTCATTGGTATTCTTTCCAACTTTGT	chr8	48734204	48734412
AMPL7153083973	*PRKDC*	GCTGTACCTCGTTTTGCTTAAAGTCA	AAAATACTGGAGTGTTCATTGATTTGGC	chr8	48736330	48736605
AMPL7153299713	*PRKDC*	GTTACTATCAACTTTATTAGCTCATTTTCTGCC	TCCCTCCTGATGTCCTCAGATG	chr8	48739107	48739253
AMPL7153241353	*PRKDC*	GACCTCACACTTACTTAGCAAGCT	CCAAGTACCACAAGCTAAACAGCT	chr8	48739202	48739468
AMPL7153083950	*PRKDC*	GCCACAGCAAACAATGTATATTTGATGA	GCATTTTTGCTTCAGATCCTAATTTGTCTT	chr8	48740682	48740957
AMPL7153083939	*PRKDC*	CCATGTTTCCTCACATAGGCAAAC	TGCACGACTAGTTTCCTTCAATGAG	chr8	48743083	48743358
AMPL7153083931	*PRKDC*	GAAGCCTGTTCACTTCGTCTGTA	CCGAGGTGAAAATGAACCAGCA	chr8	48744299	48744561
AMPL7153083921	*PRKDC*	GAAGCTATTTTAAGCACACTCAGCAA	GAGTGCCTGACCTCTCTGATTG	chr8	48746720	48746989
AMPL7153083899	*PRKDC*	CCTTACTTCTCTCACAAAAGCCCAA	TGGCGTTTCCGAAGTACTGTTC	chr8	48748800	48749067
AMPL7153083910	*PRKDC*	GTCTCCACAAACATCGGAGTGA	GGTACAAATATGCTGATGGTACCATTAGAT	chr8	48749023	48749204
AMPL7153083866	*PRKDC*	AAAGGAATGTGTATTCTGAGAAGGCAA	CATGTTCGAGCATCCTCTGTCA	chr8	48749662	48749809
AMPL7153083884	*PRKDC*	GACCACATTACCTGAAATTCGCATTC	CCATTTGCCATTGTCTAAGCAATATACTTT	chr8	48749761	48750028
AMPL7153083839	*PRKDC*	ACTCCTGAAAAATACTCTATGCTACCTTTG	TAGATGGTGTTACTGTAGTTGAACAAAAGG	chr8	48751629	48751886
AMPL7153137958	*PRKDC*	GTATAGCTTACCTGTAATTATCATGAATCCACA	GCCTGTCATACTTTGAAAGTGCAATAATAAAAA	chr8	48752565	48752820
AMPL7153298405	*PRKDC*	GCAGATATCCTATTCTTATGGTGCCTT	GGACTTCGTTCAAGTCATGAGACA	chr8	48761548	48761741
AMPL7153083789	*PRKDC*	AGGGCTAGTGTTCACTCACCTA	CCTCCTCTTGCAGACAGGTATG	chr8	48761695	48761956
AMPL7153043839	*PRKDC*	GAGGGAAGCTCTTGGTCACTTT	GCTTAACCTACATTTCCACTTTTGTGTATT	chr8	48761952	48762117
AMPL7153083768	*PRKDC*	TCAAGGGTCATAACAAATCAAAACAGAGTA	GTCAATATGACATGAGAAGGAAAATTGTCA	chr8	48765168	48765439
AMPL7153083761	*PRKDC*	GCACCATACTGAAGTCTGTCAATCC	TCTGTATTTGTCTTCCTGCTTCTTACTTG	chr8	48766573	48766848
AMPL7153083754	*PRKDC*	ACTGTAATCATCATTTACGACACACTACAT	CAGAATGGAATGCCGCATATTAATACA	chr8	48767706	48767981
AMPL7153083723	*PRKDC*	TGAATTATGTGTCATAGGCTCTATAGGCT	TTTTGGCAATTTAAGACCATTCTTTGATGT	chr8	48769640	48769913
AMPL7153083655	*PRKDC*	AGATAAATATTGGGTGGCTAATCAGAGAAC	CAAACTGGGAAATCCAATAGTACCATTAAA	chr8	48770898	48771149
AMPL7153083680	*PRKDC*	AGCTTGGCTAAGAAGAGACGGATA	TGTCTTACTTTGTCCTTGTTTTCCCATAAT	chr8	48771095	48771286
AMPL7153083617	*PRKDC*	AATGTTCATTCTCAGTATGACACCAATGA	ACTGCCTTGTCCACTGAATGTTAA	chr8	48771343	48771608
AMPL7153083606	*PRKDC*	GGCTAGGAATTAGATCTTGGAAAATTCTCC	CAACCGGAGTTCAGAGCTATTCA	chr8	48772009	48772242
AMPL7153083612	*PRKDC*	GTCTAGGGTCTTGGGAGCTGTA	CACTGGCTTGATGAAAACCTTAGATTAAAA	chr8	48772197	48772445
AMPL7153083598	*PRKDC*	CAAAACAAAATAACCCATCGATTTGAATCC	TGCCAGATTGCTACTAAAACAGAAAAAGTA	chr8	48773402	48773612
AMPL7153083589	*PRKDC*	TTGAAAGCCGACTTGAATATTGGC	GGAGTGCTTTTCCATGAGCTATTACA	chr8	48774519	48774793
AMPL7153083577	*PRKDC*	TTGAACTCCTACAATAACAATAGTGCACA	GCCATGTTGTGTGAATTGATTTGTCA	chr8	48774887	48775145
AMPL7153043719	*PRKDC*	ACATACATAAAAATATATGTACCCAAAGCATTG	AAAGATGATGTTCATGCTAAGGAATCAAAAATT	chr8	48775888	48776054
AMPL7153299716	*PRKDC*	TACACGAGCCATGGAAAACTTGATT	TTGACTGTTAAAATTCTGTCTGTCTAAAGGAT	chr8	48775996	48776254
AMPL7153043703	*PRKDC*	GTGTCTAGGCACACCAGTCAAG	CTGTAGCCTGGATGCTTTGAGA	chr8	48777052	48777198
AMPL7153240862	*PRKDC*	CCACCACAATTGTGCTGAAGAATTC	CCTCTTCAAAGCAGGGATGTTCATATAATT	chr8	48777151	48777371
AMPL7153083531	*PRKDC*	TTTTTGGAACAGAGAGGATAATTTCCCA	AACTATTGAAGCAAAAGTAAAACCCAACA	chr8	48790206	48790473
AMPL7153083518	*PRKDC*	AGAACATTTTGAATAACCTATTGGGTGGTT	CAGGGAATTTCCTCCAGGAACT	chr8	48791914	48792109
AMPL7153083526	*PRKDC*	CATGCAGTCCACATAATTATTGAACCG	TGCAGTTTCTTATGTCTGTCTTGCT	chr8	48792057	48792250
AMPL7153083513	*PRKDC*	GGGATTACTGTGCTAGTCTCCCTA	ACAAATGTGTCCTGTTTTTCTTTTCCT	chr8	48793841	48794116
AMPL7153138809	*PRKDC*	TCTTATTTGTCAAACCAATGAGCTTTTGG	TGCAACACTGGAAGAAGTGTGA	chr8	48794361	48794567
AMPL7153138858	*PRKDC*	GGAATCTTTGGCCCACCATGAA	AAAGGTAAATTTTGTTTTCTGTCATTCTCA	chr8	48794523	48794729
AMPL7153299376	*PRKDC*	AAACAGTTTATTTAAAGGGAAACTTTGTTACC	CCCAGTTATGGTACTTCTCCTTTCTTTTT	chr8	48798473	48798742
AMPL7153083490	*PRKDC*	AGAGACACCTGCAAGTGACATATG	GAAATATCTTTTTGCTAGGCATTTCAGTGT	chr8	48800036	48800310
AMPL7153083481	*PRKDC*	TCAGCATGCTCAATACATAAAGGACTT	AGAGCTCACCTAATTTACTGTTTTGTCA	chr8	48800973	48801248
AMPL7153083468	*PRKDC*	TAAGCTAGCATGGAGATCAAACATGG	CCTAGAGACCCATCTGAGAGAGAAAA	chr8	48801338	48801613
AMPL7153083476	*PRKDC*	AGTACCCACCTCTGTGCTGTTA	GCTGACGAAGTGTTTTTATCAAACAATCTG	chr8	48801565	48801819
AMPL7153083448	*PRKDC*	CCCTTCTAACAGAAACCTTTGAAGTAGT	CATTGCCATGCATGACATTATAGCAG	chr8	48802745	48802989
AMPL7153083457	*PRKDC*	CCAGTGCCAAAGCACTTTTCTG	AATCTTAGTGGAGTTGAAAGGAAGCAT	chr8	48802941	48803098
AMPL7153137841	*PRKDC*	CTCTGGCAGCACGACACACA	GCCCACCCTCTTGTACTTCG	chr8	48805654	48805832
AMPL7153137846	*PRKDC*	CTCTCGCCAATGAACGTGTTG	GATTAAAGAAATGTAGGTATATGTGTGCATTTT	chr8	48805725	48806000
AMPL7153083439	*PRKDC*	TGCAATGATACCCAATTACTCAATTCTTCT	GTTGCATGTTGGAGAGGGACTA	chr8	48809626	48809901
AMPL7153083432	*PRKDC*	AGGTTGTCCTTCATTAGCTGTGAAT	CCTTTTCAGTTAAAAATTAGGCCAACACAT	chr8	48810956	48811231
AMPL7153083428	*PRKDC*	ACCTTATAAGAGAATCACACGAGCCTTA	TGTGCCTTCTTTAATTCCATTCCTTCT	chr8	48812842	48813116
AMPL7153298404	*PRKDC*	TGCAGGAAATATGACACACGGAT	CACCAAATCGCTTTTCAAGCGA	chr8	48815035	48815233
AMPL7153137777	*PRKDC*	GGTGAAGCGCAAGGCTATAAAG	AAGGGTATTCAGTTGTCAGTGGTTATAAAA	chr8	48815189	48815420
AMPL7153083420	*PRKDC*	ACAACTAATCGATGGGAGTAAGAGGA	GCGTAACATTTCTTTAACATGAAGGGAATT	chr8	48817381	48817591
AMPL7153083417	*PRKDC*	TGACCACTGAATTAGACAAACCAAACT	TCTCTTGTCATCAACGTGATAACTTAAACA	chr8	48824902	48825177
AMPL7153043547	*PRKDC*	TTAAAGCAATAGGATCTAAATAATGGGTGAAA	GCAGTGCCCTTTAGAGAGATGAA	chr8	48826412	48826559
AMPL7153083412	*PRKDC*	AACACATCCAGGAAAATGACAGGT	GCTGGCATTAGAAATGTTTTGATTTGTGAA	chr8	48826512	48826715
AMPL7153083398	*PRKDC*	ATGACCTGGATACATCCAAATAAAAGCA	CAAGTGGGTGGCAAAGAGTTTTT	chr8	48827817	48828068
AMPL7153083385	*PRKDC*	GCCACCATGCCTGGTCAATATATTAAAA	GCCCAGAAAGGATTTAATAAAGTGGTGTTA	chr8	48830676	48830899
AMPL7153083392	*PRKDC*	AGGTTCTTTGTCTTCTTCAGATGCTT	CAGTGATGTTTAAAAGCAACCACCATTTAT	chr8	48830843	48831118
AMPL7153043518	*PRKDC*	CAAACATATTTTGAACACACTGGGCTTA	GCCTGGATGGATACCTGAAGAC	chr8	48839631	48839789
AMPL7153083378	*PRKDC*	TTCTAAACATTACCTGACAAGGCTGAA	CCTGCGTTTCAGTTTAACTTGTTTGTATTA	chr8	48839740	48839951
AMPL7153083366	*PRKDC*	GGACTCACAACAGCGATGAAGA	GCCAATTTTTGTATTTGAGAATAGGGCAAA	chr8	48840281	48840548
AMPL7153043505	*PRKDC*	AATCATTTAAAAATTACCTCTTTGCCAAATTTC	CTCCCGATTCTTGTTCAGTGTACTAA	chr8	48841634	48841784
AMPL7153083325	*PRKDC*	TAGACTTTAAAACTGCATAGGCCACAA	ACCATGGGTGTACTCATTTTCATATGAATT	chr8	48842306	48842536
AMPL7153083338	*PRKDC*	GGGCAACCTTGTAGATTGCAAAATT	CTCAAAATGTTACGTGATGGGTTTGTAG	chr8	48842481	48842684
AMPL7153083306	*PRKDC*	AAGGAATTTCCTATATTACAGCCAATAACTAAC	TGGAAAGTTATTTGTTGAGTGATTCGTGA	chr8	48843157	48843417
AMPL7153299692	*PRKDC*	CAATAGGTTACATCCAACAGAAATGCA	CCGTTTTGAAGATTGTTGAGAAATTGGAT	chr8	48845412	48845644
AMPL7153083300	*PRKDC*	CCCAACAGTCTGTATTTCAAGTGTAAG	ACATACATCTGTGTATGTGTCTTAATGCTT	chr8	48845588	48845823
AMPL7153083291	*PRKDC*	AAAGGAAGTAAGGTACATTTATGGCTTTCA	GAGAATCCATCACTACATATTGTCATGCT	chr8	48846480	48846693
AMPL7153083287	*PRKDC*	TCCTACACTTTTCAAAATGTAAGGCATTG	TTTGTGAGTTTTGATTCTGTTTTTCAGTGT	chr8	48847423	48847682
AMPL7153083273	*PRKDC*	GATTTTGGTCTACCAAAATTTAGGAGTTCA	AAAAGGGCCAGTTCTCAGGAATT	chr8	48848149	48848329
AMPL7153298406	*PRKDC*	GCAAACTTACCCACAGTACTAATGC	CTAACTCACGGTTTGCTGATTATTTCC	chr8	48848281	48848492
AMPL7153240895	*PRKDC*	TGCCACAAAGTACTAACTAAAACTGTCAT	GCTTCCTCCAGTCTGTTGCAAG	chr8	48848777	48848956
AMPL7153083267	*PRKDC*	TTACTGTGTCAAGGTACAGCAAGAC	TCACATGGCTTAACATGACACCTTAAATA	chr8	48848908	48849159
AMPL7153299703	*PRKDC*	GTATGGTAGAAAAACATCTGCATGTAAGTG	AAGGAGTTATCTATTGCTATCCGTGGATAT	chr8	48851937	48852155
AMPL7153083218	*PRKDC*	TGAGACATACTCCTGCAAAAAGTCC	GCGTGTAACTATAACAGTACATTAGTAGCA	chr8	48852100	48852372
AMPL7153083201	*PRKDC*	CAAAGGGAATTTAATTCTGTGTGTGAAAGA	TCTGTTCTGTGTTATGTCGTCTGATTTT	chr8	48855705	48855980
AMPL7153083192	*PRKDC*	TAACTGTGTGTGAAACATCACAGATGT	TTTGTACTAAAGGCAATTCGTCCTCA	chr8	48856287	48856560
AMPL7153083184	*PRKDC*	CTACCTGAGGGCACAGCATATC	GGGTTAATATAGGTGTCAAGCTGTCT	chr8	48856408	48856677
AMPL7153083174	*PRKDC*	TGGTTAAGAGTTCAAAGAAATAATCAGGCA	GGTATGATGAACTTTATGACATTCTGCTTC	chr8	48866092	48866367
AMPL7153083168	*PRKDC*	GTTTGGGCTCTCTTACTGCTGA	TAAATGTAGTTTGAGGCCTTCACTTGT	chr8	48866251	48866524
AMPL7153083161	*PRKDC*	TCGACTGTAAAATAAGACCTTGGTAGAGA	TTTTTCTTGATTCCCGCACTACATTG	chr8	48866790	48867056
AMPL7153083155	*PRKDC*	AAACACATCACCCAAAACACAACTC	GTATTGCTCCTCTTGGAGAAGTAGTTT	chr8	48868340	48868603
AMPL7153083150	*PRKDC*	AAAATCCAAGTTCCTCACACCATAAGT	GTTGACAATTTTGGTTTTGCTTTTCTTGTA	chr8	48869652	48869910
AMPL7153083142	*PRKDC*	TAGGATTTCTTCTCTACATTCACGAAACTG	CCCTTTAGGAGTGGTTTACATGATGC	chr8	48869796	48870059
AMPL7153080558	*PRKDC*	TGACAAATCCCTTTATACACATACACAGAAAT	GCTGCAGGAGACCTTGTCC	chr8	48872370	48872645
AMPL7153195768	*RAD50*	TCCAGGTCCCTGGTGAGATTAG	ATGTAGCGACCTGTAAACTGAAGG	chr5	131892980	131893187
AMPL7153195453	*RAD50*	GGTAAACTTCTGTGGTTCTCTTATAACGA	TTCCAGTGCCAAGTTTCTATAATAAGCT	chr5	131894920	131895138
AMPL7153195464	*RAD50*	TCATTTTGGATGTTTTTCTTTTTGTTCCCT	TGGGCTCTCACATCTGTTTCTTG	chr5	131911346	131911497
AMPL7153195470	*RAD50*	CTGTGTTTTCCTTCAAAGGTTGCT	ACAGACCTAAAAGACTGAAAATGAACAGA	chr5	131911450	131911675
AMPL7153201367	*RAD50*	GCCATTTCTAACGGGATAGGTGA	ACACTTAGAGCTCAGACTGACCTTT	chr5	131914875	131915042
AMPL7153195484	*RAD50*	TGATTTTCATTTTCTGTAGGCATGGTGA	GCAAACACAGTTCAATAACTTAATGGACT	chr5	131914989	131915249
AMPL7153195490	*RAD50*	CTCATTGTAACTGTAATCTTAGTGACAGCA	GCTTCCTTACTTGTAATCTGATCACGAATC	chr5	131915449	131915697
AMPL7153195493	*RAD50*	CTGAAGCAATATAAGGAAAAAGCTTGTGA	GCCAGTCCACGATGTATACTTATATCAAAA	chr5	131915638	131915849
AMPL7153195497	*RAD50*	GTCTTCATCTATCAGCCATGTAAGCT	GGATGGCAAAATGGATTCAAATAAAGTTCA	chr5	131923188	131923463
AMPL7153195499	*RAD50*	TGTTCTGCTTCAAAATTTTGGGATTATTGT	CTGTTCTCTGGTGATTGTGATATAAGTCAT	chr5	131923401	131923673
AMPL7153195501	*RAD50*	TTTTCAAGGGACTGATGAGCAACTAA	AGCATAAGTCCTTGGAGATTCTTCATTC	chr5	131923617	131923868
AMPL7153195506	*RAD50*	GGAGAAACTGGGCAAAATGTACAAG	GACTGAATTAATGAATCTCTAGCTCGGAT	chr5	131924254	131924445
AMPL7153195510	*RAD50*	AGCAGATCGCCATCAAGAACAT	CAAAATGGAGTCCAACCAAAAATTAAAACA	chr5	131924394	131924661
AMPL7153195515	*RAD50*	AATGCTCATTCTTTACATATGCATTTAGAATG	CAAACAATATCTTACAGCTTTTATGAGCTCC	chr5	131925294	131925544
AMPL7153201598	*RAD50*	AAACAGTAATATTTGGAACATTCTGAGGAGT	TCTACATTGCTGTTTTTCTCAGCCT	chr5	131926799	131926956
AMPL7153201599	*RAD50*	AATTATTGACTAGGAACGTGAGTTAAGCA	AGGTAGGCTATTTTAAGTACCAGACAGTAT	chr5	131926902	131927154
AMPL7153195530	*RAD50*	CCCACTTGAAGACTTTAAGAACTTTAAGA	TATGTAGCCAGTCTTCAAGCTGTTTTT	chr5	131927407	131927677
AMPL7153195535	*RAD50*	CTCACTGTTGGGATATTTTCCCAAC	CTTTCTGAAGTCTCCTTGTATGTAAGAACT	chr5	131927624	131927868
AMPL7153195541	*RAD50*	TTATTTGGTCATACCAAACTCTTGTCATGA	CATCAAACAGCTTGTCTTCGTAACTG	chr5	131930435	131930661
AMPL7153195545	*RAD50*	GAACTAAAAAGAAAGGAAGAGCAGTTGTC	CCTTAACAGTTACCTGTGAATGAAAATTCA	chr5	131930606	131930808
AMPL7153201633	*RAD50*	AGAAAAAGATACAACCGTATTCAGAATACT	ACTGCAAATCACTGATGACTTCTTGT	chr5	131931183	131931402
AMPL7153201634	*RAD50*	GAGTTTTTCAGACAGAGGCTGAGTT	CTACATGTACAGTGAAGGTAAATCCCATT	chr5	131931351	131931536
AMPL7153201607	*RAD50*	ATCCACTGATATGATACCCTGAAAAGAAC	GCAGTTTGTTTCTTAATTCTGGTATTTCCT	chr5	131938863	131939047

AMPL7153195567	*RAD50*	TAGGCAAAGCATAATTGATTTGAAGGAGA	CATGGAACAAGTTTTATTCTGCCTTAAACT	chr5	131938988	131939224
AMPL7153201605	*RAD50*	TTCCTTTTGTTTACATCATTTGAATTGCTG	CGATCTAAGTCTATTCCTTGTAGCTTAGC	chr5	131939418	131939682
AMPL7153195577	*RAD50*	GTTGAAAGAAAAATTGCACAACAAGCA	TCAAAGTCTCTATGAGTACAGAAGGACA	chr5	131939626	131939779
AMPL7153195583	*RAD50*	GTATTTTCTATGCCCTTACATTAATTACTGTG	AACATGTATGGATATTCTTACCTTTATCTCTCT	chr5	131940446	131940712
AMPL7153195588	*RAD50*	AAGTGCTTTTATTAAGACTGTGAAGTCTGA	CCGACGTGGTGCTATGAACATA	chr5	131944219	131944486
AMPL7153195593	*RAD50*	TCTCCTGTTATGTGCCCTTAAGTACA	TCGCATTCACTTAGTTGAGCTATTACTTT	chr5	131944749	131945024
AMPL7153201620	*RAD50*	ATTCAAGATGGGAAAGACGACTATAAGAA	GCAAAACAGACCTACCTTCTGTGTA	chr5	131944871	131945103
AMPL7153201621	*RAD50*	TGAAGATATGAGACTCATGAGACAAGATATTGA	GGCCTCTTTCCTTTTTGCATTTCTAT	chr5	131945045	131945195
AMPL7153195603	*RAD50*	CTTCACACTGGCTTATTCTCCCT	CCTTTTCATTTGCAAAACCTGCATTTG	chr5	131951548	131951823
AMPL7153195608	*RAD50*	AAAGAAAACAACATTTGAAGGAAATGGGT	GGCTGAGGCTAAAATTCTTTTAAAACAGG	chr5	131951767	131951920
AMPL7153195615	*RAD50*	AAAGAAATCTATGACTTTTCCACTTCAGGT	GTGGTTCTCGAAGTTCTTTCTTAAAATGAA	chr5	131953682	131953883
AMPL7153195622	*RAD50*	GGCGACAGAAAGGTTATGAAGAAGAAATTA	CCCAGTCTAATACTTACTGGTCAAGA	chr5	131953823	131954003
AMPL7153201647	*RAD50*	TTGCTGCTGTTTATTTTTGTAGCTCTTAC	ACCATGGTACTCACCTTGTCCA	chr5	131972632	131972906
AMPL7153201648	*RAD50*	GACCTGTGGCGAAGTACCTATC	CTACAAGATGTTGCCAAGATAAGAATTCTT	chr5	131972861	131973049
AMPL7153195632	*RAD50*	TGTAAATGACAAAAGGCTACAGAGCAT	GATGTTTGTTTCTCTCTTTCAGTTACTTGG	chr5	131973711	131973979
AMPL7153195639	*RAD50*	CTTGCCTGCCATGAGATGAGAA	TGGTTTTGAAGAACTATCACAACCTGT	chr5	131976302	131976575
AMPL7153195773	*RAD50*	CCTGACACACAGCACAAGTTCAT	GCTCCACAAAATCTTCATCATGAGTGAT	chr5	131977759	131977943
AMPL7153195777	*RAD50*	GCGTAACTTCCAGCTTCTGGTA	CTGAGGACCTACATTTCTATGGCATTTAAA	chr5	131977893	131978098
AMPL7153665564	*RAD51*	GCCTTGGCTTTTCCTAAAGTCTTT	TGTAAAGGTTGTAATACCTTCCACTAGGTA	chr15	40990843	40991118
AMPL7153665562	*RAD51*	TGTAGGACACATAACATCTGTGTTAGATT	CACTACATTCCCTTTTGTACTATTCCCA	chr15	40993187	40993462
AMPL7153665558	*RAD51*	CTACTGTGCCTGGCCTATTTATTTTTC	GCAGTAGACCAAGATCACGCT	chr15	40993812	40994060
AMPL7153665555	*RAD51*	TTGCCATCAAGATCACTGTGGT	GTTTTGTTTTATAACCTGGAAGCTTTCCT	chr15	40998308	40998559
AMPL7153560226	*RAD51*	CATCTTTCTGATGAGCTCCAAGAACA	GTACCTTTTATGGCCATCCTAACAGAT	chr15	41001131	41001406
AMPL7153560225	*RAD51*	GCCTTGGAGGAATTATAAAGATGTCATGA	AGAGACATTCTTCGGCCAAACTAAC	chr15	41010909	41011184
AMPL7153665557	*RAD51*	TTTAAGTGTCAGAAACAAATTGCTCATCTG	CCAAATAGCCATTACAAGATTCAGGGA	chr15	41020832	41021093
AMPL7153665561	*RAD51*	GTGGTAAGGAAGGGACCAGAATC	CCTAGCAAGGCTCCTGGAATTC	chr15	41021633	41021900
AMPL7153665563	*RAD51*	GGGAAAGTGGTGGCAGCATTAA	ATGTCTTCACAGTTAATATGACATTCCGA	chr15	41021975	41022231
AMPL7153665556	*RAD51*	ATTTTCAGCTCTGTTACAAAGTCAGGA	GGGAGCAGTGCACTCTCATTAG	chr15	41023171	41023441
AMPL7153420718	*RAD52*	TTCATTCTCCAGCAGCGATGAA	CATTCTTGGGCTTAGTCTAAATTAGGATCA	chr12	1022461	1022729
AMPL7153420713	*RAD52*	GAAATGTCATCCTTCCTCAAAAATTCCC	ACCAGAAACCACAAGCAAAATCTG	chr12	1022976	1023128
AMPL7153420714	*RAD52*	GCTATAAGTTTGGAGGTCCCAAGATC	CTGATGACGCACTTTTCCTTGAC	chr12	1023078	1023318
AMPL7153420711	*RAD52*	CGCCTAAAAGGTATTCTGTGGCTA	GAGAGTGTTCCCTCTAACAAGAGG	chr12	1023525	1023798
AMPL7153420720	*RAD52*	CCAGGGTTACCGCAGAGA	CCCTCTCATGCAGAAGCCT	chr12	1025420	1025662
AMPL7153420708	*RAD52*	CGGATGAGCTCAGGCTTCT	GACCCAGCCATGCTGTGATA	chr12	1025632	1025850
AMPL7153420709	*RAD52*	ATACCGGGAGCTGCAGTCCTG	CAGTTGAAGGAATTTGTTTTTCTTCTTTCCT	chr12	1025800	1026022
AMPL7153420712	*RAD52*	AGTCCTCACTTTTTCTCCTTGCAT	CTCCTGAGAAGCAACCTGTACAAAA	chr12	1034516	1034791
AMPL7153420716	*RAD52*	ACGCTGCTTGGATTTTTGAACC	CCGCTCTCGTGTTTGAGTTCAA	chr12	1036195	1036470
AMPL7153420710	*RAD52*	CGTCCAGCTACCTACTTCCCTA	GGAGGTAAGGTAGGTGGACTCA	chr12	1038898	1039147
AMPL7153420715	*RAD52*	GCAGCTGAGTCCACCTACCTTA	CATTTAAGTGTGGTAAGGAATTAACACAGC	chr12	1039120	1039368
AMPL7153420717	*RAD52*	AACAGTTAACAGCAGACTTCCGAA	GAGACTCAATGATGTGAGGAATGGT	chr12	1040275	1040549
AMPL7153420719	*RAD52*	GCTCACACATGTACTACGATGCT	TTTTCAGTAGAGGCTCTTGCCTTTT	chr12	1042046	1042320
AMPL7153775127	*RBBP8*	GTCCAAAGACGACTTGATACCTCTATAATG	AGGACTGTCATACAAAGGTATACAAAGC	chr18	20516727	20517002
AMPL7153775138	*RBBP8*	CTGGTTTATTATTTAGAGGCCAGACTGAT	ACATTAAGGTCAAATTGGACATCAGTATCA	chr18	20526303	20526559
AMPL7153775132	*RBBP8*	CACGGTGGAGCTCTTAGAATTATCC	GGAAAGATCACTTTCAATCCCAAAACTAAT	chr18	20529506	20529780
AMPL7153775134	*RBBP8*	AGTCTTGGAATTCCAAAGAAGGAAATCTC	ATGTAAACCATCCATTTAATTACAACCAATTT	chr18	20548702	20548977
AMPL7153775140	*RBBP8*	GGCACGGTGTGAGATGTAGTAC	GCTGGAGTTGTTCAGAAAGCTTTTTATT	chr18	20554917	20555172
AMPL7153775141	*RBBP8*	GTGAATGAAAGGAATACTCTACAGGAAGAA	GACACTTTCTTCAAGCACTAATCTAAGAGA	chr18	20555114	20555381
AMPL7153775130	*RBBP8*	ATCTACTTAGGTTTGGAACATTAGATGCA	CATTCAAGCTCAGCTGCTTGATG	chr18	20562037	20562216
AMPL7153775131	*RBBP8*	TGTCATTCTTCTCTAGGAATGATCAACAG	AAACCAGAGAACTAAAATACAACTCCAACT	chr18	20562164	20562391
AMPL7153775124	*RBBP8*	TCCCAATGTCTTAACTTAAACAGCTT	TTCATTAGGATTATGTTGTGGATGAGTTGAA	chr18	20564633	20564901
AMPL7153775125	*RBBP8*	CCCTTAGAAATGAGAAAAGTTTCCAAGTC	CCCACCAATAACCACAATTTTAAACTTGTA	chr18	20564841	20565007
AMPL7153775135	*RBBP8*	TGCCATTTATGGTAGGTATTTTGGACTTT	AGATTCTTCTTGAACACCAAGTCCAAG	chr18	20569007	20569281
AMPL7153775136	*RBBP8*	AATTTAGCTACAGTTGTTGCTGAAACA	GCCTTTACCCAAGATCTTTAGATAGAACAA	chr18	20569227	20569393
AMPL7153775143	*RBBP8*	TCATCCCTTTTTAAGAATGAACAAATCCCA	TCATTGTAATGGGATCTCACCAATAGC	chr18	20570835	20571071
AMPL7153774840	*RBBP8*	ATTTTCAGATTCTACTTCAAAGACTCCTCC	GCTAAAAGGGAGTGTTTTCAGATGTTTTT	chr18	20572710	20572885
AMPL7153775144	*RBBP8*	CCTTCTCTTTTACAGCCTGGGAAAA	GCTCCAAATGTTTATCAGTGTTAGAATCTT	chr18	20572831	20573096
AMPL7153775145	*RBBP8*	TGAACAGAATAGGACTGAGTACGGTA	CAGACAGATCCAGAGGTTTATCCATC	chr18	20573040	20573273
AMPL7153775146	*RBBP8*	AGTTTTCCATGAATGGAGACTGTGT	GGAGAGCATTTATTCAAGGGCTGAA	chr18	20573222	20573496
AMPL7153775147	*RBBP8*	CTGTTCACAGGAATGCATCATCC	GTTAGCTCTAAATGAAATCACAAGCAATCA	chr18	20573448	20573666
AMPL7153775123	*RBBP8*	TTATTTCTGTTTTAGAGTGCTGGTTCTCAT	TCCTATAAATACAGACACCTGAAGGAAGAA	chr18	20573668	20573943
AMPL7153775121	*RBBP8*	GTCCTTTACCAGACATATGATTTGCAAAAA	AGGAGGTTGACTAACAACTCATTGG	chr18	20576216	20576491
AMPL7153775139	*RBBP8*	AAGCAGTAAAACCTAAATCCTTACCTGTT	TACAGTCCATGTCCACTGTCTCT	chr18	20577405	20577631
AMPL7153774833	*RBBP8*	TGGCAGTCAGTCAAAATTAGGAGG	GTACAACTACAAACTTCACACAACGTTATT	chr18	20577584	20577759
AMPL7153775148	*RBBP8*	TCATGATGGTCTACATATTAAGCAAAAGCT	AACTTAACTTACTGAATTCTGCAACTGAGA	chr18	20581483	20581751
AMPL7153775137	*RBBP8*	GCCTGGAGCATGAAACCCAATA	AGACAGCTCTATTGTACTTCCAGTCA	chr18	20586204	20586451
AMPL7153775126	*RBBP8*	GGGATTATTTCTCCTCTGAACTCTAAGC	CTGGCTCAAATAAGAGGTTTCTATAACCAA	chr18	20596733	20597006
AMPL7153775142	*RBBP8*	AGCTTCTAGTTTGTAATCAATAAGCATAACA	AAATGTTACAATCTATACTCTCACCTCTTTC	chr18	20602011	20602257
AMPL7153775128	*RBBP8*	ATGTAACCATTTGGTAAATATTTGCTGTCA	GCGTTGTAAGGCTGACGTCTTT	chr18	20605894	20606166
AMPL7153775129	*RBBP8*	CTTGATCCTTGTCCTCGTCCAA	GGTGCAAAAGCAAAATATCACAAACATTTT	chr18	20606122	20606360
AMPL7153774999	*RMI1*	GAAATGAATGTGACTAGTATTGCATTAAGAGC	CCACTGCTCAAACACTTGTTTATTCA	chr9	86615898	86616069
AMPL7153775239	*RMI1*	ATGTTAACTTGAGTCAGGCCCAAA	GCTTCAGCTGTAACTAGATCATTTGTTGTA	chr9	86616019	86616260
AMPL7153775240	*RMI1*	CCAGATACAGAAGTTGAGAGGAAAGAA	CTAACACTTTCACGTTTTCTGGTTTCAATA	chr9	86616203	86616472
AMPL7153775241	*RMI1*	GAAATATATCTTTCCGTCTTGGTGTTCTCT	CTGTAAGCTCATCATTTTCATCAAGACTTG	chr9	86616412	86616676
AMPL7153775242	*RMI1*	GGTCCTTCTGATGAAGAACTCTTGG	TCCATCTGTTCTTTCTGGACAGTTTC	chr9	86616621	86616896
AMPL7153775243	*RMI1*	GGAGGAGGCCTTGCTTTTAGAA	AAAAATACTCCTGTTTTGGTCTCTAACAGA	chr9	86616848	86617107
AMPL7153775244	*RMI1*	CTAATGAAGACAAATCATTTGGTTGTCCA	TCTGTACATAGTTGACCACCTCTCTATTT	chr9	86617048	86617258
AMPL7153775245	*RMI1*	AGCAGTTCAGATAGCCATTCCTTAAATAAT	CACTTTCACTGTTGTAACTTCCTTTGG	chr9	86617191	86617431
AMPL7153775246	*RMI1*	TGTCTGTTCTAATGGCCAGCAAA	TCTCTTTGACATTTCTGCAACCCTT	chr9	86617381	86617652
AMPL7153775247	*RMI1*	TCCTCTTCAATACCAAAAGTTCCTGG	ATTGTGAACAAATTCTAAATATTTCCTTGTTGT	chr9	86617601	86617844
AMPL7153775235	*RMI2*	CCGCTGCTTCTCAAAGTTGG	GTCCGCCATCACTACCCTG	chr16	11439267	11439532
AMPL7153775238	*RMI2*	ACTATGGGCTTTTCTTCTTTTTCTAGGAAA	TGGTTGTTTTTAACGACAGTTCCAAC	chr16	11444472	11444678
AMPL7153775117	*RNF8*	GCAGCCAGAACCTAGGTCA	TTCAGCCCAGCAAGACACCTT	chr6	37321879	37322140
AMPL7153775109	*RNF8*	GTTTGCCTTTTTCTACTTGGTGGTT	GATACCAGTTGGTATGTGACACCA	chr6	37328030	37328265
AMPL7153775110	*RNF8*	AGGTGACTGTAGGACGAGGATT	GGATGGCTAGGAAAGAAGAAGTTATACTTT	chr6	37328219	37328471
AMPL7153775113	*RNF8*	AGAGAGATTCCTTTAAGAAGACGAAAATCA	GACAAGGATATATTGTCTCCCAGTCTT	chr6	37336194	37336425
AMPL7153775114	*RNF8*	ATGCGGAGTATGAATATGAAGTTACTGAAG	AGTCAACTTAGGATCCAAATTGTCAGAG	chr6	37336368	37336643
AMPL7153775115	*RNF8*	GAAAGGTGAAGTGGCCAGTACA	CCTGCATCTGTATTTTGAGCCTCA	chr6	37336591	37336812
AMPL7153775116	*RNF8*	TTTAAGGTGACCATGTCCAGGATTC	CTGAAGACCACTCTTGCCCTT	chr6	37336763	37337031
AMPL7153775119	*RNF8*	CTCTGCTTGTTGCTGGATTGTG	CCACCTGCTCTGTTTACAGACTG	chr6	37339210	37339484
AMPL7153775111	*RNF8*	CTAACTAGAGGGAGAGAACAGGCAT	CTCTCTTTATTTTCCCTAGCAACCCTT	chr6	37342304	37342578
AMPL7153775107	*RNF8*	GAAAGGAGGTTAGTGCTCTGTATTAGAAA	TCATTCTCTAGCACATCATTCATGTGG	chr6	37344497	37344772
AMPL7153775108	*RNF8*	CAAGCACAGAAGGAAGAAGTTCTTAG	CTGTAAGGGACCCAGCTACTTG	chr6	37344719	37344994
AMPL7153775120	*RNF8*	CCTAACTTTGAGATCACAAGCATCCT	TTCCCTAATGAGAACAATTCGTCGTT	chr6	37348848	37349123
AMPL7153774807	*RNF8*	TGGTAAATAATCTGAGCTCAGAAGTGAAAG	CCCTAGTTCAAGAATTGTTTATCTGACTCT	chr6	37349067	37349241
AMPL7153775112	*RNF8*	GGAGTCCACGGAAGGGAAATAC	CAAGTGACTGAGGGCTTCCTAA	chr6	37358455	37358687
AMPL7153734513	*RPA1*	AGGCTCTGAATAGGCTCTTTTAACAAT	TTTCCGAAAGTGGACAAATCTGTCT	chr17	1745974	1746249
AMPL7153734497	*RPA1*	GGCAAACAGTTTGTGTTGAACTAATG	CCCAAATTATGGCAACCAAAGGA	chr17	1747100	1747375
AMPL7153734501	*RPA1*	AGCACAGGTATGAGTAGCTCTCA	CTTAGCTGAAAACTCACTATTGCCTTTTT	chr17	1747795	1748069
AMPL7153734510	*RPA1*	GGGAAAGCATTTTGAGTAGCTTATCAT	CTGAATCCAAAAGGTAGAACTCCGAATA	chr17	1756317	1756561
AMPL7153734507	*RPA1*	CACTTTCCCAGAGCATTCCTTCT	GGAAAACTCCCTTTAGTCCCAGATT	chr17	1775638	1775911
AMPL7153734502	*RPA1*	GTGGCACCTCCAAAATTTAGAGTTATTG	CCGATTTCATAAGTACACTCTGAACTTACT	chr17	1778858	1779133
AMPL7153734508	*RPA1*	AACTGTCAAACCCATGTGTCATCT	CTCACACTTTCGTCAACCAGTTC	chr17	1780340	1780613
AMPL7153734509	*RPA1*	GGAAGGGAAGCTTTTCTCCCTA	TCTGGCCTACTATTATTCTTCACCTCATAG	chr17	1780568	1780795
AMPL7153734506	*RPA1*	GCCACTGCTCTCAAGATGTCAC	CCAGGCGGTGTCAGTTCTAAAG	chr17	1782163	1782392
AMPL7153734514	*RPA1*	GTAGTCTCAGGTTCTGTGGCTT	CTAGTCATCCCTGCTCTTCCTTC	chr17	1782472	1782740
AMPL7153734505	*RPA1*	CTTCAGTGCTTGCCGTTCATC	GGTCCTGTGCGTGTTGAAGA	chr17	1782771	1783031
AMPL7153734504	*RPA1*	CTTCTGTGTTCTTCCTGCTAGTGA	CTGATTGTAACCAGCTTTTCTCAAAGC	chr17	1783781	1784053
AMPL7153734516	*RPA1*	GTTGCATGTGGAGAAGCAGAAG	GAAACCAAGTTTCTAAATCCATCCTTCTG	chr17	1787037	1787312
AMPL7153734511	*RPA1*	AATGATTCCCTGTGATCATGTGTAATCT	GGCTTGGTACATGCAGTTCTCT	chr17	1791825	1792034
AMPL7153734512	*RPA1*	CCACAGTGGTGTATCTTCGCAA	GTCTCTACTGTTGTGACCACAGT	chr17	1791990	1792231
AMPL7153734515	*RPA1*	ACCTGGCCCTCTCTGGAAATTA	GCATTTGTCCAAGGATAGCTTCAG	chr17	1795006	1795206
AMPL7153733758	*RPA1*	AGAATCAGTGGGTGACTTGTTTCC	GGGTCAGTGTTATAGAATGAGAACCAT	chr17	1795148	1795299
AMPL7153734503	*RPA1*	AGTCTTATCAGTATTTGCAAGTTGTCCA	CTGGCAGTGAGTACAGTAACTCC	chr17	1798235	1798495
AMPL7153734499	*RPA1*	GGGCTCACTGAAACCACTGAAG	GTCACACGGAGGTGGGAGGAAA	chr17	1800276	1800543
AMPL7153755163	*RTEL1*	GCTCTGTGCCCTTCTGAGAAC	ACACCAGCCCTTTTTATGACAGAC	chr20	62290726	62290994
AMPL7153547282	*RTEL1*	CGTAACCCTTGCTCCGAACT	GCCCTCTCGGCAATCTTG	chr20	62292622	62292775
AMPL7153755165	*RTEL1*	CCTCCGAGACGGCATCTCT	TCTCCTTGAGTTCTGCTTGAGAGA	chr20	62292733	62292968
AMPL7153755166	*RTEL1*	TGCCCACTTCACCCATTTTTGA	CCGAGACAGGTGTAAACTCGTC	chr20	62293051	62293326
AMPL7153573060	*RTEL1*	GCCACGTTTCAGTTATGCTCAC	GCCTACCTGTAGATGGTTACTCTCTT	chr20	62293765	62293986
AMPL7153573061	*RTEL1*	GCATCCATCCTGAGGTGAAGAAAC	CTTACGGCACAAGTGGATCTGT	chr20	62293936	62294199
AMPL7153547232	*RTEL1*	CCTTTCTTGGCCGTGCTTC	CTGCACACACTCCAACCCGA	chr20	62294133	62294288
AMPL7153573051	*RTEL1*	GACCCTGAGGGTGTGTGTTTA	GAGCTGCTTAAACTTTTTCGTACTGAAT	chr20	62297315	62297585
AMPL7153584048	*RTEL1*	TGCTCACAGGATCTTCTCCTCT	ACATCAGGAGTCGTCAGCTACA	chr20	62298788	62298943
AMPL7153573053	*RTEL1*	CACATGTCTTGGCTTCCCTCTT	CACCTAGGGCTTCAGGAGTTTG	chr20	62303836	62304084
AMPL7153755164	*RTEL1*	CCTGTCCTGTTCTGTGGTGATTC	GACCAAGCAGTCTGGCTATGAA	chr20	62305250	62305473
AMPL7153573015	*RTEL1*	TTTTTCCATGCCAGGAATCCTG	GGATCACTGTGGGTCGAGGA	chr20	62309407	62309626
AMPL7153573041	*RTEL1*	ATTGCAAAGCTGAAGAGTAAGTGTTG	GCCTCACTCGTGATGTGACAG	chr20	62309520	62309795
AMPL7153573018	*RTEL1*	TGTTTTCTTCTCGGCAGCTACA	GATCTGGGCTTCAGCAAACAG	chr20	62311065	62311235
AMPL7153020483	*RTEL1*	CCTCGGCAGCTACATCTTTGAG	CCGATCAGAAGATGAGGTTCTGT	chr20	62311192	62311441
AMPL7153573036	*RTEL1*	CCATTGGGCAGGAGATGGA	CCCAAGTCTAGAATCAGTTAACTAATACAG	chr20	62311895	62312165
AMPL7153020780	*RTEL1*	ATGCTGCAGAAAGTCGGGT	CCTTACCTTTTAGGACCAGAAGACC	chr20	62316813	62317088
AMPL7153573046	*RTEL1*	GGGTCTTCTGGTCCTAAAAGGT	ATCCACGGCTGGGCATGTG	chr20	62317062	62317316
AMPL7153584919	*RTEL1*	GCAGCCTCCTTTGTTCTGACT	TGCCGCTGGTAAGGATGAG	chr20	62318826	62319083
AMPL7153020907	*RTEL1*	ACAGGGAAGGTGCTGAGCTA	CGCTGATGTCAGAGGTGTCA	chr20	62318986	62319177

AMPL7153020935	*RTEL1*	CGGTGACACCTCTGACATCAG	CCCTCACCGTCTGTCAAACG	chr20	62319154	62319410
AMPL7153573047	*RTEL1*	CATCATCGACAAGCACCAGATCT	GCACTCCTCGGAAAACCTAGAG	chr20	62319319	62319507
AMPL7153573019	*RTEL1*	CTGCTCCCAAGAGCTGGTAG	ATCCGATGAGGGCAAGCAGGA	chr20	62319580	62319833
AMPL7153573048	*RTEL1*	CCTGCACTTCCAGGCAACAT	CTCCATCTTCCTGGCCAAGTC	chr20	62319640	62319907
AMPL7153020981	*RTEL1*	GCGACTTGGCCAGGAAGATG	TTTGTAAGATAAGAGGCAACACTCAGG	chr20	62319884	62320131
AMPL7153584918	*RTEL1*	CCTCCTGTCTGAGTAGCCCTA	GCGGCAACCCTTGCATAGTAA	chr20	62320165	62320439
AMPL7153021030	*RTEL1*	TTTCCTTCCAGACCATCAGTGC	AGGAAACTCCCATCCTGCCT	chr20	62320396	62320671
AMPL7153584923	*RTEL1*	GGTCCTGACCCACAGATGGA	AGGAACTGCATCTTGAGGACAAC	chr20	62320719	62320964
AMPL7153584053	*RTEL1*	GGCCGTGGTGTGATTGTCA	TCAGTCACCAGACCGCCTTA	chr20	62320887	62321048
AMPL7153573035	*RTEL1*	GTAAGGCGGTCTGGTGACT	CACCTGTGGTCACAGAGGAA	chr20	62321027	62321221
AMPL7153021096	*RTEL1*	CTCTCTGGGCAGGAGTGGTA	GAAGTGAAGACCCTCATCTGCT	chr20	62321105	62321379
AMPL7153573039	*RTEL1*	CAGCAGATGAGGGTCTTCACTT	GTCTCGGATGACATGGCCAAA	chr20	62321356	62321530
AMPL7153573040	*RTEL1*	CCACGTCAGGGTGTATGACAAC	CTTAGCTTTCCTGGTGGAGAAGAAG	chr20	62321487	62321751
AMPL7153021136	*RTEL1*	CCTTCTTCTCCACCAGGAAAGC	CTAGGAGGGAGAGGCAGCCTT	chr20	62321725	62321941
AMPL7153013653	*RTEL1*	CCCGAGAGTAGCCTGTGTG	AGGGTCTGTGTCCATCCCAA	chr20	62322177	62322336
AMPL7153584912	*RTEL1*	TGTGCGTCCAGGTGCTTT	GCGTTCCCATGCGTGTCTA	chr20	62323018	62323245
AMPL7153008089	*RTEL1*	TCCCAGGGAACGCTCAATG	CTTGGCTCAACTCCTGCTTCA	chr20	62324060	62324233
AMPL7153021311	*RTEL1*	CTGGTGCACAGACGGACAG	AGACCACCTTGGACCCAGAT	chr20	62324170	62324442
AMPL7153584043	*RTEL1*	GGTCCAAGGTGGTCTCTGTTC	TGCTCAGGCCGATAGCCAC	chr20	62324427	62324589
AMPL7153021374	*RTEL1*	CAGGGAACTTTCCAGATGCTCT	CACTCAAGAGTTAGCCTCGTGAA	chr20	62325668	62325918
AMPL7153021397	*RTEL1*	GCAGCTCTCCAGGAGTTCCT	CTTGTCGAGGTCGTCGTCTT	chr20	62325994	62326266
AMPL7153573059	*RTEL1*	GCAGCGCTGACAGCCTATAA	TCTGTGCACGTCTGTGAGAAG	chr20	62326224	62326476
AMPL7153021415	*RTEL1*	CAGGGTTCAGCATGTTTGTGC	CTGGAGACAGAGACTTGAGTCTGA	chr20	62326415	62326680
AMPL7153755167	*RTEL1*	CCCTCAGACTCAAGTCTCTGT	CAGCGATGTCTCTCCCATGA	chr20	62326653	62326860
AMPL7153573014	*RTEL1*	GGCCACTACAGTTCCTGCT	TGATCAAGCCACGTTGGGT	chr20	62327013	62327253
AMPL7153775094	*SLX1A*	CGACAGGGACATCACGGTTC	GGAAAGCGAAGGCTGTCTCTC	chr16	29466688	29466952
AMPL7153775095	*SLX1A*	CTCGCCTGCGAGGAGAGACA	CTCCGCGTCATCAAAGGGA	chr16	29466919	29467146
AMPL7153775089	*SLX1A*	TTCTTCATTTGTAAGATGCAAATAACAGTCAC	CAAGGGCATTGGCCCTCTAG	chr16	29468806	29469080
AMPL7153775102	*SLX1A*	GTGCCAGATGGACACTGAGAAA	CATAAGGCACCCTTGACTCAGA	chr16	29469287	29469552
AMPL7153775104	*SLX1A*	CGAGAGCTTGTTCCGAAGCAA	CGAGCAGTGTTGACAGTGAAC	chr16	30205294	30205524
AMPL7153775105	*SLX1A*	CTACCTGCTCTACTGCCTGAAC	CCGGTCCTTCTCAACAGGC	chr16	30205452	30205680
AMPL7153775097	*SLX1A*	CTCGCCTGCGAGGAGAGACA	CTCCGCGTCATCAAAGGGA	chr16	30206261	30206488
AMPL7153775100	*SLX1B*	CGAGAGCTTGTTCCGAAGCAA	CGAGCAGTGTTGACAGTGAAC	chr16	29465952	29466182
AMPL7153775101	*SLX1B*	CTACCTGCTCTACTGCCTGAAC	CCGGTCCTTCTCAACAGGC	chr16	29466110	29466338
AMPL7153775090	*SLX1B*	GAGGAGTTTCTTCAGGAAGAACCA	GAGATACAGAGTCAGATGCCACAA	chr16	29469021	29469225
AMPL7153775093	*SLX1B*	GCTTGTGGCATCTGACTCTGTA	AAGGACACTGAGGATCAGGTCT	chr16	29469199	29469459
AMPL7153775096	*SLX1B*	CGACAGGGACATCACCGTT	GGAAAGCGAAGGCTGTCTCTC	chr16	30206030	30206294
AMPL7153775098	*SLX1B*	TTCTTCATTTGTAAGATGCAAATAACAGTCAC	CAAGGGCATTGGCCCTCTAG	chr16	30208148	30208422
AMPL7153775099	*SLX1B*	GAGGAGTTTCTTCAGGAAGAACCA	GAGATACAGAGTCAGATGCCACAA	chr16	30208363	30208567
AMPL7153775106	*SLX1B*	GCTTGTGGCATCTGACTCTGTA	AAGGACACTGAGGATCAGGTCT	chr16	30208541	30208801
AMPL7153775103	*SLX1B*	GTGCCAGATGGACACTGAGAAA	CATAAGGCACCCTTGACTCAGA	chr16	30208629	30208894
AMPL7153420615	*SLX4*	CATCAGTTCCGCTCCACCT	CCCTGTACCAGAAGGTGCTG	chr16	3632340	3632554
AMPL7153420616	*SLX4*	CAGCTCAAAGGGCTGGTACA	ACGTCCTGGTCTGGCTCTT	chr16	3632513	3632718
AMPL7153420622	*SLX4*	GGGCCAGAGGATACATGAGG	CAAGGGACCTGCTAAGACCAAG	chr16	3633047	3633283
AMPL7153420623	*SLX4*	GCTTTCATGATGCTTCCTTTGATGTC	CTGAAGCTGAAGGAGATATTCCAGTAC	chr16	3633228	3633474
AMPL7153420624	*SLX4*	AGTCCAGGGTCTGGTGAGT	GAACGTGGAGTGTGGACCA	chr16	3633428	3633572
AMPL7153420611	*SLX4*	CCAGGTGTGTGAAACCCAGTTA	TCACGCTACTGGGATCAGATGT	chr16	3634695	3634946
AMPL7153420633	*SLX4*	CCCACCCTCTTAGTGTAAAATAGTAACA	CAGAGGAAATTGCAAGAGAAGTCCT	chr16	3638937	3639186
AMPL7153420634	*SLX4*	ACAGAGCCGAATTCAGAAAGCTC	CTCTCGCCAATTCCCATTGAC	chr16	3639107	3639342
AMPL7153420093	*SLX4*	TCCGCTCCAGGTTCCAGTG	GACAGTGACGATGAGCAGGAG	chr16	3639302	3639450
AMPL7153420635	*SLX4*	GGGCTTCTGTTGGCCTGAT	ACTCCCGTCTCTCCAGGAA	chr16	3639400	3639663
AMPL7153420636	*SLX4*	TTGCCTTCTGCCGTCAGAA	GCAGAAGCCGTGACTGTTCT	chr16	3639624	3639848
AMPL7153420637	*SLX4*	GAGGCTGCTGATTTGGGTCT	GCTGTTTTCCATCATTGATGTTGATGC	chr16	3639804	3640069
AMPL7153420638	*SLX4*	GCTCTGGGAAGGTTCCTGATCT	ACGAAGAAGATGAGGTCATCCTCT	chr16	3640020	3640202
AMPL7153420639	*SLX4*	GCTCCTCATCCGAGTCCAGTA	TCTCCAGCTGTGCCATCAAAG	chr16	3640157	3640416
AMPL7153420640	*SLX4*	GGATGCTCCTGTCCCTTTTCTG	GTGAGCCCGAGGAACAAAGTGG	chr16	3640373	3640634
AMPL7153420641	*SLX4*	GCCAGGCGATGAGAAACCTC	TCTTCCGCACTCAGATGATGC	chr16	3640570	3640720
AMPL7153420642	*SLX4*	TTGATGAGAAGAGCTGTTCGTAATCC	GTTGGAGCCAGGAAGAGATGAG	chr16	3640670	3640915
AMPL7153420644	*SLX4*	CCACCTTGTCCCACTGTTTCTG	ATCCAAGGACCACGAAGAAGATCAAGAAAA	chr16	3640928	3641134
AMPL7153420645	*SLX4*	TAAATTTCTTCCATTTCTGCTTCATTCACG	CGATGACTGTCGATGTTTCGTGT	chr16	3641074	3641339
AMPL7153420646	*SLX4*	CTGCCAGTCCTCGTCAGTTAAT	CTCCAAGTTACAACTCTCTGATCAGTAAA	chr16	3642651	3642921
AMPL7153420625	*SLX4*	TTGGGAGGGAGAAGGTGAGAACAT	GGTCAATAACCCACACCTGAGT	chr16	3644297	3644562
AMPL7153420626	*SLX4*	TGTCCGTCTGAAACTGGACATC	TTGGAGAGGAAAGGAGAATGCAGGT	chr16	3644518	3644734
AMPL7153420613	*SLX4*	GCTCACGGATGTCAGGATGT	TGTGAATCCTCCACTCAGGGTT	chr16	3645443	3645712
AMPL7153420614	*SLX4*	ATGGCACCACAAACCCAGTCAGA	GAAGGTGTCCTCGGATAGCAAG	chr16	3645643	3645827
AMPL7153420627	*SLX4*	TAGAGTCCCACCCAGCATCT	CTTTCAGGGCCTTATGCAGGA	chr16	3646130	3646401
AMPL7153420628	*SLX4*	CATCTCACCTGCAGTCCCTTC	CACTCTCCTTACTGGCTTCTTTCA	chr16	3646145	3646419
AMPL7153420604	*SLX4*	CAGTTCTGGGATTGCCAACCT	CTGAGGAAGTGGAATTGTCTAGCA	chr16	3647317	3647588
AMPL7153420605	*SLX4*	TTAAAATCCTGCTGGCAGGAAGT	TTGATGGTGTCGCTTTTGTTTCTC	chr16	3647534	3647725
AMPL7153420610	*SLX4*	ACACAAACCTGCTTCTGGTCTTA	CCAGTAACTTTTCTCTTTGGCTGTTT	chr16	3647789	3648031
AMPL7153420612	*SLX4*	TACTGCGTCCAGCCCATAAAAA	CTTACCTTAAAGAGCAGAACCAGTCA	chr16	3650882	3651113
AMPL7153420063	*SLX4*	CTTCACAGCACACTGCTTCAAG	TGGTTCTCCTGTATTGACTGTTTTCC	chr16	3651065	3651223
AMPL7153420620	*SLX4*	GGGTAGGTTGACAACAAAGCTG	GTTTGTTCTTCTGCCAGATTTGTCAA	chr16	3651990	3652194
AMPL7153420621	*SLX4*	CGTTCATGGCTGAGAGGTTCTT	CTCACGGATCTTTGGTGCTTCT	chr16	3652146	3652336
AMPL7153420619	*SLX4*	AAGAAACTCAACAAATGCTGATTCCTT	GCACAGCACAATTGGTCCTACA	chr16	3656379	3656624
AMPL7153420074	*SLX4*	GCTCTCTTGAACTGCTGCATTC	ACAGATGGTTGTGAATATTTTCCTCCTT	chr16	3656578	3656732
AMPL7153420606	*SLX4*	CCCTTTCCAGGAAGTTTTCCAG	CCCTAGGACTAAAAAGCAAAGGGTAAC	chr16	3658381	3658615
AMPL7153420607	*SLX4*	CGGTTCACTTGCTTGCCATTTG	GCTAGCTTTTTCCAAAGGGTGAAAAA	chr16	3658566	3658800
AMPL7153420608	*SLX4*	CCTGACACTTCCTTGATTCCATGT	CAAACTCTCCAAGTATCTAATGGAGAAGTA	chr16	3658750	3659008
AMPL7153604817	*SMARCA5*	TGCGACGTAGCATCCAGG	TCGCCCTCACCTCCATCTCG	chr4	144434989	144435264
AMPL7153775074	*SMARCA5*	TAAGATGTGTTTTGGTCAGAAATTGGTCT	GTGGTATTGTTGTAACACAAAATGGACTAA	chr4	144438419	144438687
AMPL7153775076	*SMARCA5*	GCATAGTGCTTTTGAAACTCATCCAT	TCATCTTCAAAGGTGAAGTTGGAGT	chr4	144442428	144442690
AMPL7153775077	*SMARCA5*	TCATTCAACCTGCTGCTCAGAAG	CCACTGAAGACTATCGAAAATAAGTTTGGT	chr4	144442642	144442807
AMPL7153775087	*SMARCA5*	GTCAGCCACTTGACTATGATGTTTAAAC	CAAATAGTAGCCAACACAAATCCAAAACAT	chr4	144445433	144445708
AMPL7153775081	*SMARCA5*	ATGTGTAGGCTTAGATGAAGAGATTGC	GCTAACAGTTGTAGACTGTAAGAAACTTTT	chr4	144446526	144446796
AMPL7153775079	*SMARCA5*	TCATTTTTCACTGCTTCATGGTTATTTTGAA	GCCTGAATTCAAATTATTTTAGCCTCAAAAATG	chr4	144447393	144447668
AMPL7153775070	*SMARCA5*	ACTTGTCATCAGTAAATGTAATGCGTAGT	CAAATTCTTACATGAAGGCTTTTCACATCT	chr4	144448951	144449220
AMPL7153775072	*SMARCA5*	GTCCTTGCAGAGGATATAAACTCATTCTC	TGTAGTCTTGAATTCCCTCACTATTTCTGA	chr4	144449550	144449768
AMPL7153775073	*SMARCA5*	ATGGTGTATTTGGTTCTTTTCTTTCAGTTG	AAAAGGCAGGCCACTTTTATTACAAAAAT	chr4	144449708	144449951
AMPL7153774739	*SMARCA5*	AGGGTATTTGTTAATATTATCTCTGATCTGAGT	TCCCATCATAAATATGCTTTAACACTACTCC	chr4	144451554	144451721
AMPL7153775069	*SMARCA5*	ATCACTTGATACTCACGCAGAGAAATT	GGGAAGAATCAATAGTGCTATCAAGTACAT	chr4	144455886	144456161
AMPL7153774734	*SMARCA5*	GGATTAAGTATGTAAAGTGGCAGGGATT	TTAGGATGTTCAATAACCTCATTTTGTCCA	chr4	144457529	144457687
AMPL7153775068	*SMARCA5*	TATAGATATACTCAACTCAGCAGGCAAGA	TGCTCCTTTGTTTTAACATGAGCATTTTAA	chr4	144457628	144457896
AMPL7153775075	*SMARCA5*	CCCTTAAATTTTTCTACTTTTGAAGCCACT	GTCCTATGGATAAAACAAAATGGTTGTCAA	chr4	144459684	144459941
AMPL7153775066	*SMARCA5*	CCCAACAGGTTCACGAGTACTA	TGTTTGGTTCATTGTATGCATTGATGG	chr4	144459734	144459969
AMPL7153775063	*SMARCA5*	CAGTGTACCCATTTCAGTGTTATTTATGTG	TAAGACGATTTTCAAACAAAAACAGGTTGT	chr4	144461444	144461719
AMPL7153775078	*SMARCA5*	AAGTTTCAAGTTTCCAGTAAAATAAAGTCTGT	ATTTCCTGTTTCTGGTCTAAAACATTCTATA	chr4	144464595	144464870
AMPL7153775083	*SMARCA5*	TCCTTAAAGAGAAGCTTTTGTGTACGT	TCCAGTAAAATAAAGCTTTCATGTAACCCA	chr4	144464901	144465173
AMPL7153775064	*SMARCA5*	CTGAATGCTTACTCTGTTGACTAGACA	CGTTTAGGTGGTTCAATCCACTCT	chr4	144465686	144465924
AMPL7153775065	*SMARCA5*	CCCATGTGCTAGATTGCATTCAC	CTGAACTCCTGGTAACAGAGTGAG	chr4	144465877	144466152
AMPL7153775086	*SMARCA5*	GTTGCTTGCAGAATTGGCTGTT	ACCTTCCATAATTTCTGGATAGGAATGAA	chr4	144466517	144466790
AMPL7153775088	*SMARCA5*	AAACTTGTGAAATGTCAGTTAGTTGGTTC	CCCAACATAACCTAACACAGTCCT	chr4	144466994	144467269
AMPL7153775060	*SMARCA5*	GAAGGTTTGCTAATAGCATGTTTTCCAA	TCAGAATAAGAAAACAAGAACCTTCCAACA	chr4	144467855	144468130
AMPL7153775061	*SMARCA5*	GGTTAACATGTGAGGGTATGCTGT	TGTCAAGTGCTTTCTTGATGCTTATTCT	chr4	144468388	144468651
AMPL7153775062	*SMARCA5*	GGGAGAGGCGAGAATTCAAAGA	CCATGTTTGAATGCTACTCTGAAGAAATTT	chr4	144468601	144468791
AMPL7153774761	*SMARCA5*	ATTCTGATTTGAAATGCAACATTTTAGCT	CCATATGATATTCTCAGCTGATGAAAAGGT	chr4	144468973	144469127
AMPL7153775082	*SMARCA5*	ACTTTCATAGATTGGACGGTACAAAGC	ACTCTGAACTCTGCTACATTACACAAAA	chr4	144469070	144469338
AMPL7153775071	*SMARCA5*	ATTGAAATCATGAAACTTGTGGATGGAAAG	GCCAAGCAGTGTTAAATACTTAGAAAGG	chr4	144471090	144471330
AMPL7153775080	*SMARCA5*	GGGAAAAGTCTCATACCTATTACTTTGTAG	GACCCGTAAATTAAAGAACTACTGGTTT	chr4	144474148	144474395
AMPL7153496631	*TP53BP1*	ATCTCCACGATAGCAGGGAATAAAAC	GTTGAGTGTCCTTTCCTTCTTCACT	chr15	43699536	43699811
AMPL7153496706	*TP53BP1*	GCCTAACACCTACAGGTCTAAGGA	GCAGAAAGGCAGTTTGTTGGAT	chr15	43700075	43700349
AMPL7153496422	*TP53BP1*	TGCTGTCCTCCTGCCTATACAA	CCCATTGGCTCTGCTTCTTTTCT	chr15	43701047	43701322
AMPL7153496382	*TP53BP1*	GCCTCTTCTCAACACAGCAAGTT	GTAAGAAGGAGGCATAAGGTAGCAAAT	chr15	43701773	43702014
AMPL7153461382	*TP53BP1*	TACTTGCCTTGCCTCCTCTACA	CTTTGCCTCTCAACAAGACCTTG	chr15	43705206	43705445
AMPL7153496423	*TP53BP1*	GGAGAAATGCGTAGCCCAGAAA	TGGTGTGTATCCCTCTAGTAACTGTG	chr15	43705400	43705574
AMPL7153461420	*TP53BP1*	GAAGGGCCACTGCCACATATTA	GGAGTTCTCTCAGGCAAAAGAAAACTTAT	chr15	43707693	43707882
AMPL7153496687	*TP53BP1*	GGACCGTTCCTCTTCAGAAGTG	ACCAACCTGTTTGGCTTATCCTG	chr15	43707831	43708036
AMPL7153496431	*TP53BP1*	ACCATCTGAGAAGCCACACATAG	AGGCAGCAGATATCAGCTTAGGTA	chr15	43708295	43708443
AMPL7153496432	*TP53BP1*	AGCTGTTCAGGAAGCAAAAGTCT	AGCAAGCTTCTTTTATGCTTTCCAC	chr15	43708396	43708646
AMPL7153496676	*TP53BP1*	AAAAATCTCTACAAACTCTGCTTCTTTCA	CTTAGATGCCTCCTCTCCAGGA	chr15	43712469	43712741
AMPL7153496677	*TP53BP1*	CACGGAGCCCTACAAAGCTATT	GTTCTCACCGGCTTCTCCTATC	chr15	43712697	43712971
AMPL7153496428	*TP53BP1*	TCTCCTCCATTCCCTTGTTTTCTG	GAGACTTTGTGAAGAAAAGATTCAGTAGC	chr15	43713161	43713436
AMPL7153496650	*TP53BP1*	CTGTGGCTCTCTCTCTCTGGA	ACAAGTCTCTCAGCTATGCACAG	chr15	43714017	43714183
AMPL7153496651	*TP53BP1*	CCTTTCCCTGAGCTTCCACTG	CATTGTGTTATTATGGCATCTTTTATCTTCTGT	chr15	43714139	43714376
AMPL7153461411	*TP53BP1*	TGAAACAAAATCACGCAACTGCATT	GGCCATGTCTTACATCGTCACAT	chr15	43720097	43720330
AMPL7153496642	*TP53BP1*	GCGTACTTCCCGGATTGTTCTC	TGAAGCATAAGATTTGTGATTTTGACAGC	chr15	43720285	43720438
AMPL7153496104	*TP53BP1*	CTCTGAGAATCCTGAAAGTTAAAAACAAGG	GGACCAGAACTTTGGAAAGCAAG	chr15	43724331	43724497
AMPL7153496417	*TP53BP1*	CTCTCAGTCTGAACTGTGGCAT	GCAATGGTGACAGATGTGCTAGA	chr15	43724452	43724697
AMPL7153496418	*TP53BP1*	TACTCCGTCCTTCTTTCTGGTCT	CACTTGCTTAATTGACATGGCTTCTTTAT	chr15	43724651	43724919
AMPL7153461404	*TP53BP1*	CAAACCACAGCACAGTACATATTAGGA	GCATTTTATTTCCTCTGTGCATAAAAGGA	chr15	43730431	43730706
AMPL7153496430	*TP53BP1*	CCTTTGTTGATAAACCATCTACCCTCA	CAGAGTGAAGGTTTAGGTGTGATACTAAC	chr15	43733647	43733859
AMPL7153496425	*TP53BP1*	CCCAGATAATCTGCAGCTACTAATTACC	CACCATAGCAACCAGTGATGTCAT	chr15	43738535	43738766
AMPL7153496426	*TP53BP1*	GGTCTCCACCATGCTTTCAGAC	GCAAATTGATGACCTTGAATTGCTGTTT	chr15	43738720	43738872
AMPL7153496661	*TP53BP1*	CACCCTTTCTTTCCTTCCTCTCTTTA	CCACCTCTTATTGGGCACCTAA	chr15	43739363	43739618

AMPL7153461416	*TP53BP1*	CTGTGTCTCTTGGGCTCCAATT	GGAGCAAAGGAAAAGAGTGATTGAAATATT	chr15	43739574	43739808
AMPL7153496496	*TP53BP1*	TCGCCAACTTTCCATTCCAGAT	CCAAACTCAGGAGAAAACAAGTAATTCATT	chr15	43748024	43748232
AMPL7153461398	*TP53BP1*	GCATTAGCCATTTTTGAGTCTTCTGTT	CCAGTCATGGGAGGATATTGCT	chr15	43748175	43748439
AMPL7153461399	*TP53BP1*	TATTCTCAGCACATGGTTCTATTTCTGG	AAAGAATGCTCAGAAGCTATGGAAGTT	chr15	43748389	43748663
AMPL7153461400	*TP53BP1*	GGGAATCAATACTAATCACACTGGTTTC	GTTAGATCAGGAGGAAGCTATGGAAATT	chr15	43748608	43748868
AMPL7153461401	*TP53BP1*	CCTCCTCTGGATGGTGTTCTTT	GTGAAGTACAACTGAGTCAGAATGATGA	chr15	43748818	43749067
AMPL7153461402	*TP53BP1*	GTGTCTGTATCATCTCCCTTTGTTTTG	GAGGATCTTGGGCTATCTTTGACAG	chr15	43749012	43749287
AMPL7153461403	*TP53BP1*	AAAGCATCAACTTGCAAGAATCCC	ATGATCATGTACAAATTACAATTTGTCTCTTTC	chr15	43749238	43749462
AMPL7153461370	*TP53BP1*	ACATTTAAAACGTGTGAATTTTATGGTATGT	ACCAATATCTCAGAGCACACCAG	chr15	43761955	43762128
AMPL7153496414	*TP53BP1*	ATAGGAAGTGACCCAGGAGGGAAGA	TGTTCTTAGTGGTTTTGGAACATGAGTAAT	chr15	43762080	43762340
AMPL7153496404	*TP53BP1*	CGAATTGTGTAGGTCACAAGCATT	CAAATACAGAAATGCAAAACTGTGGCTA	chr15	43766754	43767029
AMPL7153496427	*TP53BP1*	GCCTCTTTCAGCCTTAGCCTAA	GCAGTACTGAATGGTTTTTGGCA	chr15	43767690	43767964
AMPL7153496429	*TP53BP1*	AAAAGTTTAGATCTTTGGAGAAGAGCTGT	GAACTTAGGAACTTTTTCTCCTGGTGAT	chr15	43769739	43769998
AMPL7153496420	*TP53BP1*	ACATTAGTATAACCCTACAGCAAAACTCG	CCTTCCTGTCATTTTATAAAACTCTCAGCA	chr15	43771537	43771752
AMPL7153496421	*TP53BP1*	TCGTTGGACTGTTCTTCATGCTTAAT	GCTGAGGTTTTAAGTTATTGGTTACTTTCC	chr15	43771696	43771889
AMPL7153496424	*TP53BP1*	CTACCTCTAATATGGGAGAAAACACAACA	TGTACATTGCATAATGTTGTGGGATGA	chr15	43771997	43772271
AMPL7153496620	*TP53BP1*	GAGAAATCTGCAACCTAATAAAGAGGAGA	GGGAACACATGATTATATCCTTCTGCT	chr15	43772989	43773253
AMPL7153461387	*TP53BP1*	CCTTTTATCCTTGGGATGAGGCAA	GTGTCACTGATTAGATTGGTATTGTCTGT	chr15	43783778	43784048
AMPL7153496390	*TP53BP1*	ACAGACAATACCAATCTAATCAGTGACAC	GGTTCAATGAACATTTGAAAGAAAACAAGG	chr15	43784019	43784235
AMPL7153461367	*TP53BP1*	TAGCTTTAAACACCTCACCTGCAA	CCGAAAACCAGCAAACATAAGTATTTAGAA	chr15	43784181	43784432
AMPL7153496419	*TP53BP1*	AAAATCTGCTCAATCTTCTTCAAACAAGG	AGGTTACACGGAACTGATCTTTTGTTAT	chr15	43784442	43784704
AMPL7153461392	*TP53BP1*	CCATCCCTTCAGACCCGAAATC	ATATTGTGTGACGTGACGGGAAA	chr15	43785166	43785381
AMPL7153420185	*XPC*	TCAAATGGGAACAGGTGGGAAG	GGACTCTCTTCTGATGAAGAGGAGG	chr3	14187450	14187623
AMPL7153420186	*XPC*	CCGCTTCTGCTTGAGAGCT	GGAACTTGCTGCCTCTTCATG	chr3	14187574	14187743
AMPL7153420680	*XPC*	CTGAGCTGCATCTCCAAGAAACT	AGGAGAGGCTATATTCTAAAGTGGTGT	chr3	14188726	14189001
AMPL7153420156	*XPC*	GCTGTCCCTCAGTCCTGCATA	GCAGGTTCGAGGGTTGACATC	chr3	14189377	14189539
AMPL7153420682	*XPC*	CATCCCTGTGTTTAGCCTCCAT	GTTTGGGAATGTGTACCTCTTCCT	chr3	14190021	14190217
AMPL7153420195	*XPC*	GACACAGCCAATAGGCATCATG	AAGGTAAGGGCAGCATCAGAAG	chr3	14190165	14190316
AMPL7153420170	*XPC*	CTTTCCGAGCACGGTTAGAAAAG	TGTTCTGAGGGTTCACCAGGTA	chr3	14190414	14190560
AMPL7153420679	*XPC*	CCGGGAAAGTGAGACATTTCTGA	GGCTGTGGACTGAGTTACCTTT	chr3	14193697	14193960
AMPL7153420663	*XPC*	GCTAATCCCATGCCATCAGGAA	ATTTCCTCTTCTAAATAACATGCTCAACCT	chr3	14197780	14198045
AMPL7153420664	*XPC*	TCAAAAACAGGAATAATTTTAATAACCTGACTG	TTACAAGTACGCCACCAAGCCCAT	chr3	14199445	14199709
AMPL7153420665	*XPC*	ACTGTCAATGCCCACCACATAG	GCTCTTCAAGCAGTAAAAGAGGCAA	chr3	14199660	14199875
AMPL7153420666	*XPC*	CTCACCATCGCTGCACATTTTC	AAAGAGGAGAGTGGGAGTGATGA	chr3	14199828	14200092
AMPL7153420667	*XPC*	ACTGGAGAGCTCAAAATCAGAGC	CAAACAAGAGGAAACCTTTGCTAAGG	chr3	14200035	14200285
AMPL7153420668	*XPC*	CCTCTTCCCTTTGGCACTTGG	AAAAGCAAAATTTCTTATTCTGTTTAAGGGA	chr3	14200227	14200420
AMPL7153420676	*XPC*	TTAAGTGTGACAATTTCCTGTCAATTGC	CCCTGTCTTGAACAAGCACCATAA	chr3	14201181	14201425
AMPL7153420662	*XPC*	GCACATGGCTGCCATTATCATT	CCTCTTTTTATTTTCTTGGCTGGAAATGAA	chr3	14206244	14206515
AMPL7153420673	*XPC*	GCAAGATCATACCTGGCATTTCTC	CGAGATGTGGACACCTACTACCT	chr3	14206765	14206968
AMPL7153420674	*XPC*	CCTTACCACTTCACCAGGTTTGAG	CTTTGGCAGCAAAAATTCCTCCT	chr3	14206921	14207158
AMPL7153420681	*XPC*	CAGCAAAGCCAGAAATAAAGCCTC	TCATCCGAGGAGAAGGAATTGC	chr3	14208582	14208840
AMPL7153420670	*XPC*	GCATGTGACAGGAGCCTAGAAG	CCTTGATTCTGTTCAGTACAGTAGCTATT	chr3	14209717	14209955
AMPL7153420660	*XPC*	GCTCAAAAGAAAACAAAAGGATTGCAATT	GAACACTAGGATGTCTGAGTCTTCTTC	chr3	14211829	14212104
AMPL7153420683	*XPC*	ATGAGAAAAATCAAGACCCGAGACAA	CCCTCACCTTATGTTCTGTGTTGT	chr3	14214321	14214593
AMPL7153420671	*XPC*	CCTTGCTCTTGGCCTTGGAT	GTGACTAGGCCTCCAACGAA	chr3	14219989	14220188
AMPL7153506487	*XRCC1*	CCCAGGGAGCCTCTTAGAGTTT	CCTCGATGACACATTCCCGTAT	chr19	44047425	44047700
AMPL7153151413	*XRCC1*	GTGGAGTCCATACGGGAATGTG	GAGTAGGACGTCAGTGCTGATT	chr19	44047669	44047901
AMPL7153151446	*XRCC1*	CCTTCTCCATCCTCCTGATGGT	GGGCCTCATGCTTACCTTAGC	chr19	44048181	44048424
AMPL7153151464	*XRCC1*	CAAGTCCCAGCTGAGAACTGAG	GCTCTGCATGCTCACTCTCTAC	chr19	44049905	44050149
AMPL7153142859	*XRCC1*	GGTGCCAAGAGGAGTAGAGAGT	GCCTTGCATTGACTGAGCTTCT	chr19	44050114	44050288
AMPL7153142770	*XRCC1*	CAGGCTCTCTTCCCTCAGACT	GCTCCAGGAAGATATAGACATTGAGG	chr19	44050612	44050784
AMPL7153151438	*XRCC1*	CCTCAGAGTCTGACCTGACTGTAC	CACTCTTGCCTCTTGCAGGTA	chr19	44050732	44050959
AMPL7153151531	*XRCC1*	ACTCATGTCATCCTCCCACTACA	AGTATTTTAGTAAATGCCAATGGCTGTTG	chr19	44050982	44051221
AMPL7153151515	*XRCC1*	TTGCCCAGCACAGGATAAGGAG	CTCAGATCACACCTAACTGGCAT	chr19	44055676	44055884
AMPL7153151421	*XRCC1*	GGGAGAAACAAAAAGAGGTTGGAGA	GCGAGATAAGGCCCTAGAGCTT	chr19	44055962	44056237
AMPL7153151428	*XRCC1*	GTCCAGTCTGGCCGATACTTG	GGTGCTAACCTAATCTACTCTTTGTCTTC	chr19	44056189	44056461
AMPL7153151496	*XRCC1*	CCCTGTGGAAACAAGGGATCTA	CAAGGTGAAATCATCAGACTTTGGTG	chr19	44056916	44057137
AMPL7153151453	*XRCC1*	GGGTCTTCTTTTCTTCTTGGTTCAAA	CCACTTTCTCCCTTCATAGTCACAG	chr19	44056999	44057262
AMPL7153151455	*XRCC1*	GGGTAGCAGCTGCATAGCTAG	CTGGTGGGTTTTGGCAACAAT	chr19	44057198	44057343
AMPL7153151524	*XRCC1*	CACGAGTCTAGGTCTCAACCCTA	CAGGTAAGCTGTACCTGTCACT	chr19	44057498	44057763
AMPL7153151490	*XRCC1*	TAAGGTCCCGCAAGGTCAGTAT	GAAGGAGCCAGGAAGAGGTTTC	chr19	44057665	44057933
AMPL7153151470	*XRCC1*	ATCCCAAACCCATTTCAGGGAA	ACCGGGTCAAAATTGTTTGCAG	chr19	44058636	44058835
AMPL7153151474	*XRCC1*	CACCCTAGGTACCTTGCTGTAG	TAGAAGCTCTCATCTAAGCCAACCT	chr19	44058785	44059018
AMPL7153151482	*XRCC1*	GCAGAGGTCAGTATGGGATCCA	GTGATCCAAAGGAGATTCATCCTGA	chr19	44065021	44065259
AMPL7153151502	*XRCC1*	GTGACTAAACCCACTGCCAGTT	CTTGGGAGGACTCAAAGGAATCA	chr19	44078968	44079243
AMPL7153506488	*XRCC1*	GCCGGAACGTCCCTAATTCA	CCCATACTCTACCTCATCCTTCTG	chr19	44079394	44079659
AMPL7153262135	*XRCC2*	GCTTGCGTAGTACCCTGCAAAA	TCTCCAAACAAGATGATTCTCAAAGCA	chr7	152345685	152345848
AMPL7153090251	*XRCC2*	CAACGTGAAACTAATGAAAATTGGTTGC	TACAGGAGTCTACTCTGAGGAAATGTTC	chr7	152345793	152346067
AMPL7153090256	*XRCC2*	GTCATTTACAAGCTTCTCTAAGCACTGA	ACAGACTATCCCAAAGCTCTGAAGA	chr7	152346011	152346277
AMPL7153090261	*XRCC2*	AAAAATCTTCCCAGGCAGTATTTGATTATT	GAGCTACTGCATTTTGACTGAGATTTTTAA	chr7	152346222	152346489
AMPL7153087990	*XRCC2*	CTCTTGTGAGGAGTATGTGTATACATGTG	TGAAATCTTACAGACTTTCGGAAAATGGTA	chr7	152357692	152357963
AMPL7153050011	*XRCC2*	CCTTGTTCCCATCTCCCTCA	GCAGTTGGTGAATGGCGTTG	chr7	152373083	152373228
AMPL7153572485	*XRCC3*	CCAGGCAGACGCGTTTTAAA	TAACCAGCTCCTGGTGAGACT	chr14	104165055	104165309
AMPL7153572486	*XRCC3*	CCTTCGGCACTGATCGTGTA	TCTGGGACGAACGTGTTTCC	chr14	104165168	104165352
AMPL7153572487	*XRCC3*	GGTTAGCCCAGGTTATGCCAAG	TGTGCCTAACCATCGAGAAGAC	chr14	104165304	104165577
AMPL7153383730	*XRCC3*	GCTTCCGCATCCTGGCTAAAAATA	TGTTGGAGTGTGTGAATAAGAAGGTC	chr14	104165641	104165906
AMPL7153546422	*XRCC3*	GCTGTCAAATTCACAGCGGAAT	TTGCTGACCAGCATAGACAATGA	chr14	104165799	104165951
AMPL7153775048	*XRCC3*	CATGACCCATGCTGTGTTCAGA	CTGCTTCAGAAGCTCCGATTTG	chr14	104169369	104169566
AMPL7153775049	*XRCC3*	CCACGTGCTCGATGAAGATCTG	ACTTGATTTGTGTGGCAGTCACA	chr14	104169517	104169734
AMPL7153775047	*XRCC3*	GTGTAGGACAAGCAAGATGGGAA	GTTTTACACTTTTCTGGACCAGACTTG	chr14	104174698	104174973
AMPL7153572490	*XRCC3*	TGGAGAGGTTGGTCAGTCTCTT	AAAGCACCTTTTGTCACTGTGTTG	chr14	104174924	104175073
AMPL7153572491	*XRCC3*	TAATCAGGGTAGGCAAAGGAAGGA	CCTCCCACAGGCTTTGAATTGA	chr14	104177256	104177527
AMPL7153775055	*XRCC4*	ATTTTCTTATTTCCTTGGTGTTTGTGTAGC	CAGAAACAAGGTGGATTCTGCTTATTTT	chr5	82400623	82400775
AMPL7153775056	*XRCC4*	TCCCATTACAGGTATTAAGAAATGGAGAGA	TGAGGACTGAAGAATAGCACACTTAAATTT	chr5	82400717	82400937
AMPL7153775051	*XRCC4*	AAATGTGTAGTATAGGGATTGATTTTACTTGTT	AGTTCACCAACATATTTCCCTTTTTCCA	chr5	82406706	82406916
AMPL7153775052	*XRCC4*	CAAGAAGCTGATGACATGGCAA	CAAGCTTTTGCTTCTGAGGTGTTC	chr5	82406866	82407140
AMPL7153775058	*XRCC4*	CAGGCTTCTCAATCTTGATATTTTCCC	ACATAAAGAGGGCTCCGTATTTTTATACC	chr5	82491531	82491805
AMPL7153775057	*XRCC4*	AAGCAGAACTGCATGACTAATTTGTTTAC	GTTCATTAACTTTGGGAAGAAATTCCACTT	chr5	82499325	82499599
AMPL7153775054	*XRCC4*	GGGTGAGCCAGTTATTTATAATTCTACCT	GCCCAAATAAGATATTCAACAGAGGAGATA	chr5	82500590	82500788
AMPL7153775059	*XRCC4*	ACAGCAAGTTAATACTTACAAATGATGTGC	CAAAAATGTCATCTCACTTCTTGGCA	chr5	82554277	82554540
AMPL7153775053	*XRCC4*	GTTAGAGAAATTAGTCTCTTTATCTCTTTCCTT	GCTGTTTCTCAGAGTTTCTAAAGACATGTT	chr5	82648769	82649031
AMPL7153774710	*XRCC4*	AAAAGGAGCACATCTCAGCTGAA	TGCTGACTTGAAATTCTCCTTTTTCATTTT	chr5	82648978	82649146
AMPL7153775043	*XRCC5*	CCTTTCAGGCCTAGCAGGAAAC	CGATCCCACGATTCCTGCTT	chr2	216974033	216974262
AMPL7153775026	*XRCC5*	AATACAATAAGCAAGGGACATTCTTACAGT	GCTGGTATCTTGCATTAGTAGGTAGAG	chr2	216977658	216977932
AMPL7153775032	*XRCC5*	TGCTTCCAGATTGTTCTAATATGGTTTTCA	GGAAATTTCTTAAAGTCTTCTCTGGCAGAA	chr2	216981329	216981604
AMPL7153775033	*XRCC5*	GCACGGTGGACTTCATTGGAAA	TGCAGCTTGATTAACAATGAATCACAAAAT	chr2	216982387	216982619
AMPL7153775041	*XRCC5*	TCTCTCCTCAGTGACCAAGTGT	GGCCCATTCACTATATTAGGAAATCATAGG	chr2	216983679	216983951
AMPL7153775044	*XRCC5*	GGTAAGAGTCGTTTGACAGATGAGA	CTTCCATCTTCCTTGCCAAGTGA	chr2	216986656	216986817
AMPL7153775045	*XRCC5*	CTGTTGATCTTTCTTAATAGCTTGCCTTTC	GTGGGTACAATTAAAAATTCCCATCAATGT	chr2	216986764	216987019
AMPL7153775027	*XRCC5*	CAAATGCATCATCCAGCACAAGT	ACACACACATGAATTGTCTGTTCCTTATA	chr2	216990569	216990840
AMPL7153775038	*XRCC5*	CAATTGGAATCCTAACTTTCCTTGTTTTCC	CATCATGTTGAAGAAAATGTCTTTTAGGCA	chr2	216992207	216992452
AMPL7153775025	*XRCC5*	GTTAATGTATGCATGGAACTAATTTCAGCT	AAAATGACCAATCAAAAGCATACCATTTCA	chr2	216995514	216995785
AMPL7153775023	*XRCC5*	GAGGATTTTGAATGGAATTTCTTGGCA	CACTCCTATTGCAAGAATTCATGTCATTTT	chr2	216996955	216997226
AMPL7153775030	*XRCC5*	AGTTCTGTTGTGAAAACTCTCACATGT	CATTAACAGCTGAGAAACACTGCTTAG	chr2	217001748	217002023
AMPL7153775034	*XRCC5*	TTTCTGTTATGCTACTTTCACAGAATTTGG	CCAACAACTAGCAGACAGGTGAT	chr2	217002716	217002979
AMPL7153775024	*XRCC5*	TCTACGTAGAAAGCATTTGTTTTAGTGGA	CGCAACTTTGCACTTAGCTCTCT	chr2	217005835	217006108
AMPL7153775039	*XRCC5*	ATCACTTAGCCCTCCCACACTA	CTTTTTCTTGGCTTCAATCAGAGGAAAA	chr2	217012699	217012964
AMPL7153775040	*XRCC5*	TCAGATTCCTCTCTCTAAAATAAAGACCCT	GCTTCTTCCTCTCACCTTTTAAATTCCATA	chr2	217012906	217013118
AMPL7153775037	*XRCC5*	TTTTTGGTGCTTGGAAAATAAGTTTCAC	GAACGGACTAACAAAAATTGATGCACT	chr2	217024704	217024970
AMPL7153775042	*XRCC5*	AGCACACTTATTACATTAAGCCATCTTGT	AGTGTTACAACAAAAGGCTACAGTTCTTA	chr2	217026607	217026865
AMPL7153775031	*XRCC5*	ACTTATAGGCACAAACACAGAAGCA	CCAGGATGCTGTATGCCTCTTT	chr2	217054867	217055134
AMPL7153775028	*XRCC5*	GCCTTCCACAGATGGAAGGATT	AGTTGTTAAAGCGCTGCTCTTCT	chr2	217057159	217057389
AMPL7153775029	*XRCC5*	CCCAACTTGCTACCACAGTTTTC	AAGTCATCATGTTTCCCACATAACTCATTA	chr2	217057343	217057589
AMPL7153775046	*XRCC5*	GAGGAGGTGTGATGGAAACGAA	TTCTTCGAGGGTATCAAGAAAATAAAGTCC	chr2	217059562	217059782
AMPL7153775035	*XRCC5*	CCTTAGGTTTTGCTGTGGCAAT	AAGCAACAAGTGTAAGATAATCAGCCT	chr2	217068949	217069214
AMPL7153775036	*XRCC5*	AGCGGTGAATAAATGAGCAAGTACA	CCAGGTAACCTGCTGGGAATTT	chr2	217069777	217070052
AMPL7153240827	*XRCC6*	ACAGATCTCACAAGAACCTAGAGGT	CAGGCCATCAAACAGCAAAGTC	chr22	42017913	42018184
AMPL7153240831	*XRCC6*	GGTAAACAAATACGAAAACAAGGACAAACA	ATGCCCAGCCAATACTGTTTTTAAATTT	chr22	42024066	42024287
AMPL7153240826	*XRCC6*	GGTACTGAGCACTTATGGAGCTTC	AGTCCTCTTTGATCAGAGTACTGATATTCT	chr22	42032050	42032324
AMPL7153235001	*XRCC6*	GGTAAAGGCAGCCACTGACATT	CACTTCACTGAGTGAGTAGTCAGATC	chr22	42032455	42032623
AMPL7153235002	*XRCC6*	GGACAAAAACGTTTCCAAGACATGATG	GGGTTGTCTTCATTGGTGAACA	chr22	42032563	42032709
AMPL7153235003	*XRCC6*	ATTCAAGATGAGTCATAAGAGGATCATGC	AACAATAATTAAGTGCCAATTTAAGAGAACGG	chr22	42032658	42032820
AMPL7153240808	*XRCC6*	TTTTTCCTCCCTCACTTTTGTTTACC	CTGCTTCTGATTTAAGGGCAGTGTA	chr22	42033575	42033847
AMPL7153240855	*XRCC6*	ACAGTGAAGCTTTGGTGTTATGAGG	CCTGAGACCTCTTGGTATCGCTA	chr22	42042813	42043087
AMPL7153235018	*XRCC6*	CAAGTACAGGCGGTTTGCTTC	CTGTAGCGAGTCATGTTCGC	chr22	42043039	42043183
AMPL7153240790	*XRCC6*	ATTTTGCATCTCCTCGTAGCCTT	GTAAACTGCACTGTGTGTTAGAGTC	chr22	42046671	42046946
AMPL7153240816	*XRCC6*	AAGAGAAGAAAGGAGGAAACCTTTTTAGG	ATTCCAAAGTATGGGCAAGGTTCA	chr22	42049473	42049747
AMPL7153240839	*XRCC6*	ATTTGGAGCTGAGAAACAAGTGACT	GCCTATGCCCAAGCTGTTTCTAATAA	chr22	42052835	42053109
AMPL7153240845	*XRCC6*	GGACCTGTAGTATCTCAGGTGGTT	GGGAAATGCACCCAAATTACTGG	chr22	42054166	42054441
AMPL7153240849	*XRCC6*	TTCTCAGAGAGTTCTGGAAGTGGA	AAGGATATTGCCCACTGTGATAAGAC	chr22	42057254	42057528
AMPL7153775022	*XRCC6*	CTAGGCTGGCATCACAGCTTTA	AGCGTACCCTTGCTGATGTG	chr22	42059499	42059707
AMPL7153240800	*XRCC6*	GGAGTATTCAGAAGAGGAGCTGAAGA	ACACATTTTAACTGGCTGAGGACAA	chr22	42059659	42059897

The genomic positions were determined using GRCh37/hg19.

Supplementary Table S4. Primers used for pyrosequencing and expression analysis

Gene symbol	Primer sequence			Annealing temparature (˚C)
	Forward	Reverse	Sequencing primer	
Pyrosequencing				
FGF5	GTTTTTGGAGGATTTAGTTGTTAACG	CCCGCGACTTTATATACTCACTAAC	GGATGAGTGGTTTTGGTA	54
ZNF585A	GATATGGCGTATGTTTTGAATAAATTTG	AACCCACATTTCCAATAACTTTCTC	TAGTTAATAGGGAGTAGATA	58
ZNA585B	AGTATTTTTTTTTATGGATTGTTGATATTG	CAAACCTAATCACACTAATTACAATCTTACAT	AGTTTAAGGGTAGAGAATAT	60
Cancer cell fraction				
RAPGEFL1	GGTTAATTTGGAGTTGTTGTTGTAG	CTTAATAAACTTCTTAAACAACTAC		59
TFAP2B	GTTTTGTTTTAGTTTTTGGGTTTTG	CAARAAAAAACTATCACCCATACCC		63
ARHGEF4	TGTTTTGTTGTGTTGTAATTTGTT	AACCAACAAAAAAATCAATAC		57
Expression analysis				
GAPDH	GGTCGGAGTCAACGGATTT	TGATGGCAACAATATCCACTT		56
FGF5 (long)	GCTGTGTCTCAGGGGATTGT	TGAAAACGCTCCCTGAACTT		62
FGF5-S (short)	CCAATATGTTAAGCCAAG	TTATTCAGGGCCACATACCA		57

Supplementary Table S5. List of mutation in ESCC tissues and cell lines

Sample name	Panel	Gene	Type	Chromosome	Region	Coverage	Mutant allele frequency (%)	Nucleotide change	Amino acid change
MEN008	Repair	*ERCC6*	Deletion	chr10	50669409	102	12.7	c.3972delA	p.Pro1324fs
MEN008	CP1	*TP53*	SNV	chr17	7578271	360	30.3	c.578A>C	p.His193Pro
MEN018	CP1	*TP53*	SNV	chr17	7577573	1036	66.3	c.708C>A	p.Tyr236*
MEN041	Repair	*GEN1*	SNV	chr2	17962457	192	15.1	c.1978C>G	p.Leu660Val
MEN041	CP1	*TP53*	SNV	chr17	7577127	2308	34.4	c.811G>A	p.Glu271Lys
MEN041	CP1	*TP53*	SNV	chr17	7578412	624	31.7	c.518T>A	p.Val173Glu
MEN043	*No mutations*								
MEN047	CP1	*ABL1*	SNV	chr9	133750384	1605	46.0	c.1215G>C	pTrp405Cys
MEN047	CP1	*TP53*	SNV	chr17	7578271	599	25.5	c.578A>C	p.His193Pro
MEN049	CP1	*EP300*	SNV	chr22	41527628	1684	51.5	c.1519A>G	p.Ser507Gly
MEN049	CP1	*TP53*	SNV	chr17	7579414	101	57.4	c.273G>A	p.Trp91*
MEN055	CP1	*CDKN2A*	Insertion	chr9	21971170^21971171	616	67.0	c.230_231insCCTGC	p.Ala77fs
MEN055	CP1	*FBXW7*	Deletion	chr4	153259008	64*	65.6	c.807delG	p.Met269fs
MEN055	CP1	*KRAS*	SNV	chr12	25378562	3482	28.4	c.436G>A	p.Ala146Thr
MEN055	CP1	*TP53*	Deletion	chr17	7577546..7577547	378	63.2	c.734_735delGC	p.Gly245fs
MEN064	*No mutations*								
MEN066	CP1	*KDR*	SNV	chr4	55946220	722	18.8	c.3959C>A	p.Ser1320Tyr
MEN066	SWI/SNF	*SMARCA2*	SNV	chr9	2039526	2565	75.8	c.416C>G	p.Ser139Cys
MEN073	*No mutations*								
MEN081	Repair	*XRCC6*	SNV	chr22	42033692	166	76.5	c.670G>C	p.Ala224Pro
MEN083	Repair	*GEN1*	SNV	chr2	17962601	165	18.2	c.2122A>G	p.Ile708Val
MEN089	SWI/SNF	*PBRM1*	SNV	chr3	52595786	403	64.3	c.4285G>A	p.Glu1429Lys
MEN089	SWI/SNF	*PBRM1*	SNV	chr3	52597427	1427	67.4	c.3958G>C	p.Glu1320Gln
MEN089	SWI/SNF	*PBRM1*	SNV	chr3	52598088	567	64.4	c.3853G>C	p.Asp1285His
MEN089	CP1	*PIK3CA*	SNV	chr3	178936082	685	22.5	c.1624G>A	p.Glu542Lys
MEN101	CP1	*TP53*	SNV	chr17	7578190	212	38.7	c.659A>G	p.Tyr220Cys
MEN112	CP1	*TP53*	SNV	chr17	7577094	2227	28.5	c.844C>T	p.Arg282Trp
MEN112	CP1	*TP53*	SNV	chr17	7578239	475	32.8	c.610G>T	p.Glu204*
MEN113	Repair	*PRKDC*	SNV	chr8	48811085	716	36.2	c.3409A>T	p.Ile1137Phe
MEN113	CP1	*TP53*	SNV	chr17	7577130	1109	50.8	c.808T>A	p.Phe270Ile
MEN113	CP1	*TP53*	Deletion	chr17	7577577	637	34.7	c.704delA	p.Asn235fs
MEN115	Repair	*ATRX*	Insertion	chrX	76909596^76909597	142	12.7	c.4194_4195insA	p.Glu1399fs
MEN115	Repair	*PARP2*	Deletion	chr14	20822342	243	11.5	c.738delT	p.Cys246fs
MEN115	Repair	*PRKDC*	Deletion	chr8	48825023	108	19.4	c.2881delC	p.Leu961fs
MEN115	Repair	*SLX4*	Deletion	chr16	3641257	167	10.2	c.2382delC	p.Asp794fs
MEN115	CP1	*TP53*	SNV	chr17	7578190	541	67.5	c.659A>G	p.Tyr220Cys
MEN116	CP1	*TP53*	Deletion	chr17	7577558	689	50.2	c.723delC	p.Ser241fs
MEN122	SWI/SNF	*ARID2*	SNV	chr12	46287422	5000	27.8	c.5281G>C	p.Glu1761Gln
MEN122	Repair	*EME1*	SNV	chr17	48456915	2522	19.0	c.1372G>A	p.Glu458Lys
MEN122	Repair	*PARP2*	SNV	chr14	20824819	1566	17.9	c.1339C>T	p.Pro447Ser
MEN122	SWI/SNF	*SMARCE1*	SNV	chr17	38787055	7234	44.7	c.938G>A	p.Arg313His
MEN125	CP1	*TP53*	SNV	chr17	7577538	1507	20.2	c.743G>A	p.Arg248Gln
MEN126	SWI/SNF	*ARID1A*	SNV	chr1	27087443	669	80.0	c.2017C>T	p.Gln673*
MEN126	CP1	*TP53*	SNV	chr17	7578479	297	87.9	c.451C>T	p.Pro151Ser
MEN130	CP1	*TP53*	SNV	chr17	7578406	266	25.6	c.524G>A	p.Arg175His
MEN134	CP1	*TP53*	SNV	chr17	7578478	148	77.0	c.452C>G	p.Pro151Arg
MEN140	SWI/SNF	*SMARCA2*	SNV	chr9	2033069	4666	20.5	c.343C>G	p.Gln115Glu
MEN140	SWI/SNF	*SMARCA4*	SNV	chr19	11170724	6034	30.9	c.4874G>A	p.Arg1625Gln
MEN152	*No mutations*								
MEN160	Repair	*ATR*	SNV	chr3	142266604	2434	14.7	c.3320A>T	p.Tyr1107Phe
MEN160	CP1	*TP53*	SNV	chr17	7579355	262	40.8	c.332T>A	p.Leu111Gln
MEN169	CP1	*ARID1A*	SNV	chr1	27088742	113	61.9	c.2351G>A	p.Gly784Asp
MEN169	CP1	*CDKN2A*	SNV	chr9	21970984	216	59.7	c.374A>G	p.Asp125Gly
MEN169	CP1	*CDKN2A*	SNV	chr9	21970987	215	60.5	c.371G>A	p.Arg124His
MEN169	CP1	*CDKN2A*	MNV	chr9	21970990..21970991	183	56.8	c.367_368delCAinsGG	p.His123Gly
MEN169	SWI/SNF	*SMARCA4*	SNV	chr19	11095980	838	29.0	c.254C>T	p.Ser85Leu
MEN169	CP1	*TP53*	Deletion	chr17	7577076..7577077	704	69.6	c.861_862delGA	p.Glu287fs
MEN176	Repair	*BRCA1*	SNV	chr17	41223094	1632	43.4	c.4900A>G	p.Ser1634Gly
MEN176	Repair	*BRCA1*	SNV	chr17	41244000	1361	55.4	c.3548A>G	p.Lys1183Arg
MEN176	Repair	*BRCA1*	SNV	chr17	41244435	784	53.8	c.3113A>G	p.Glu1038Gly
MEN176	Repair	*BRCA1*	SNV	chr17	41244936	1038	58.7	c.2612C>T	p.Pro871Leu
MEN176	Repair	*BRCA2*	SNV	chr13	32910842	2103	57.5	c.2350A>G	p.Met784Val
MEN176	Repair	*EME1*	SNV	chr17	48458188	1344	51.6	c.1640G>A	p.Arg547His
MEN176	Repair	*ERCC5*	SNV	chr13	103515085	1748	35.4	c.2948G>C	p.Cys983Ser
MEN176	Repair	*ERCC5*	SNV	chr13	103528002	227	99.6	c.4672G>C	p.Asp1558His
MEN176	Repair	*ERCC6*	SNV	chr10	50678369	528	41.7	c.3637A>G	p.Arg1213Gly
MEN176	Repair	*ERCC6*	SNV	chr10	50678717	1550	57.5	c.3289A>G	p.Met1097Val
MEN176	Repair	*NLRP2*	SNV	chr19	55493728	1809	38.0	c.662C>T	p.Thr221Met
MEN176	Repair	*SLX4*	SNV	chr16	3639827	1427	46.9	c.3812C>T	p.Ser1271Phe
MEN176	Repair	*SLX4*	SNV	chr16	3640274	1593	58.4	c.3365C>T	p.Pro1122Leu
MEN176	SWI/SNF	*SMARCAL1*	SNV	chr2	217288388	3384	57.9	c.1129G>C	p.Glu377Gln
MEN176	SWI/SNF	*SMARCC1*	SNV	chr3	47632276	3795	47.7	c.3095G>A	p.Arg1032His
MEN176	CP1	*TP53*	SNV	chr17	7578529	744	11.3	c.401T>C	p.Phe134Ser
MEN176	Repair	*TP53BP1*	SNV	chr15	43724646	2268	55.0	c.3421A>C	p.Lys1141Gln
MEN176	Repair	*TP53BP1*	SNV	chr15	43762196	781	61.7	c.1249G>A	p.Gly417Ser
MEN176	Repair	*TP53BP1*	SNV	chr15	43767774	578	50.2	c.1074C>G	p.Asp358Glu
MEN188	*No mutations*								
MEN192	CP1	*FLT3*	SNV	chr13	28592610	417	23.7	c.2535G>C	p.Arg845Ser
MEN204	Repair	*PRKDC*	SNV	chr8	48811114	1162	17.6	c.3380G>T	p.Cys1127Phe
MEN204	CP1	*TP53*	SNV	chr17	7578437	157	67.5	c.493C>T	p.Gln165*
MEN208	CP1	*TP53*	SNV	chr17	7578394	169	81.7	c.536A>T	p.His179Leu
MEN211	*No mutations*								
MEN217	SWI/SNF	*ARID1A*	SNV	chr1	27059239	8127	72.6	c.1876G>A	p.Glu626Lys
MEN217	CP1	*PIK3CA*	SNV	*chr3*	178938877	5092	19.2	c.2119G>A	p.Glu707Lys
MEN217	CP1	*TP53*	SNV	*chr17*	7578212	15492	37.9	c.637C>T	p.Arg213*
MEN232	*No mutations*								
MEN224	Repair	*LIG4*	SNV	chr13	108862021	141	12.1	c.1596A>T	p.Lys532Asn
MEN234	CP1	*FBXW7*	SNV	*chr4*	153249385	14417	49.3	c.1393C>T	p.Arg465Cys
MEN234	SWI/SNF	*SMARCC1*	SNV	chr3	47777592	4051	43.8	c.508A>G	p.Ile170Val
MEN234	CP1	*TP53*	SNV	*chr17*	7578263	24433	59.5	c.586C>T	p.Arg196*
MEN239	Repair	*ATRX*	SNV	chrX	76939847	544	28.1	c.901G>C	p.Asp301His
MEN239	CP1	*PIK3CA*	SNV	*chr3*	178938877	5607	22.3	c.2119G>A	p.Glu707Lys

MEN239	Repair	TP53BP1	SNV	chr15	43749322	842	26.7	c.1484C>G	p.Ser495Cys
MEN245	CP1	CDKN2A	SNV	chr9	21971161	6230	48.1	c.197A>G	p.His66Arg
MEN245	CP1	PIK3CA	SNV	chr3	178938877	7618	18.2	c.2119G>A	p.Glu707Lys
MEN245	CP1	TP53	Deletion	chr17	7577110	26705	22.2	c.828delC	p.Cys277fs
MEN248	CP1	RB1	Deletion	chr13	49033843..49033846	14323	76.4	c.1980_1983delCCGG	p.Arg661fs
MEN248	CP1	TP53	SNV	chr17	7578461	11544	83.8	c.469G>T	p.Val157Phe
MEN261	CP1	PIK3CA	SNV	chr3	178938877	15465	10.3	c.2119G>A	p.Glu707Lys
MEN261	CP1	TP53	SNV	chr17	7578402	9694	83.6	c.528C>G	p.Cys176Trp
KYSE30	CP1	ASXL1	SNV	chr20	31022641	904	32.2	c.2126C>T	p.Ala709Val
KYSE30	CP1	CDKN2A	SNV	chr9	21971000	117	99.1	c.358G>T	p.Glu120*
KYSE30	CP1	HRAS	SNV	chr11	533874	719	70.1	c.182A>T	p.Gln61Leu
KYSE30	Repair	MDC1	SNV	chr6	30680916	1535	41.7	c.803G>A	p.Arg268Lys
KYSE30	Repair	SLX4	SNV	chr16	3641037	1366	26.6	c.2602G>A	p.Glu868Lys
KYSE30	Repair	SMARCA5	SNV	chr4	144464680	752	98.9	c.1922A>T	p.Asn641Ile
KYSE30	CP1	TP53	SNV	chr17	7579358	332	24.1	c.329G>T	p.Arg110Leu
KYSE50	Repair	ATR	SNV	chr3	142281919	3121	43.8	c.325C>T	p.Arg109Trp
KYSE50	Repair	BRCA1	SNV	chr17	41223048	2471	34.9	c.4946T>C	p.Met1649Thr
KYSE50	Repair	BRCA1	SNV	chr17	41245738	2431	32.5	c.1810A>G	p.Lys604Glu
KYSE50	Repair	ERCC3	SNV	chr2	128050217	4699	16.3	c.440G>C	p.Gly147Ala
KYSE50	Repair	MDC1	SNV	chr6	30680916	979	99.4	c.803G>A	p.Arg268Lys
KYSE50	Repair	RTEL1	SNV	chr20	62326159	724	49.3	c.3247G>A	p.Ala1083Thr
KYSE50	CP1	TP53	SNV	chr17	7579386	334	100.0	c.301A>T	p.Lys101*
KYSE140	SWI/SNF	ARID2	SNV	chr12	46123647	232	34.5	c.28C>A	p.Pro10Thr
KYSE140	Repair	BRCA1	SNV	chr17	41244376	2160	35.5	c.3172A>G	p.Ile1058Val
KYSE140	Repair	MDC1	SNV	chr6	30671883	322	12.4	c.5077C>G	p.Pro1693Ala
KYSE140	Repair	NLRP2	SNV	chr19	55489186	268	57.5	c.392C>T	p.Ala131Val
KYSE140	CP1	TP53	SNV	chr17	7578271	18951	99.6	c.578A>G	p.His193Arg
KYSE170	Repair & SWI/SNF	ATRX	SNV	chrX	76874328	494	99.6	c.5394A>C	p.Arg1798Ser
KYSE170	Repair & SWI/SNF	ATRX	SNV	chrX	76952116	762	19.0	c.319G>C	p.Ala107Pro
KYSE170	Repair	ERCC3	SNV	chr2	128050217	4457	33.5	c.440G>T	p.Gly147Val
KYSE170	Repair	RTEL1	SNV	chr20	62326159	546	27.1	c.3247G>A	p.Ala1083Thr
KYSE170	Repair	SLX4	SNV	chr16	3639605	544	55.3	c.4034G>A	p.Arg1345His
KYSE170	CP1	TP53	SNV	chr17	7577094	740	20.3	c.448C>G	p.Arg150Gly
KYSE170	Repair	TP53BP1	SNV	chr15	43767844	626	98.4	c.1004C>G	p.Ser335Cys
KYSE180	SWI/SNF	ARID2	SNV	chr12	46230745	1427	38.1	c.994C>T	p.Leu332Phe
KYSE180	Repair	ERCC2	SNV	chr19	45854919	1046	75.9	c.2251A>C	p.Lys751Gln
KYSE180	Repair	ERCC4	SNV	chr16	14015919	3343	32.9	c.239G>A	p.Gly80Glu
KYSE180	CP1	HRAS	SNV	chr11	534289	278	10.4	c.34G>A	p.Gly12Ser
KYSE180	Repair	SLX4	SNV	chr16	3640248	1499	27.9	c.3391C>T	p.Pro1131Ser
KYSE180	SWI/SNF	SMARCA1	SNV	chrX	128602759	585	77.6	c.2689G>T	p.Glu897*
KYSE180	CP1	TP53	SNV	chr17	7578265	759	99.9	c.188T>C	p.Ile63Thr
KYSE220	Repair	BRCA1	SNV	chr17	41245740	1169	28.6	c.1808C>T	p.Ser603Leu
KYSE220	CP1	CDKN2A	SNV	chr9	21971153	179	98.3	c.205G>T	p.Glu69*
KYSE220	Repair	PRKDC	SNV	chr8	48711777	1157	38.5	c.10290G>T	p.Met3430Ile
KYSE220	Repair	PRKDC	SNV	chr8	48746866	863	44.4	c.8042C>T	p.Pro2681Leu
KYSE220	Repair	RTEL1	SNV	chr20	62317178	1136	19.9	c.1373C>T	p.Thr458Met
KYSE220	CP1	TP53	SNV	chr17	7577539	563	98.0	c.346C>T	p.Arg116Trp
KYSE270	CP1	EGFR	SNV	chr7	55259524	1407	26.7	c.2582T>A	p.Leu861Gln
KYSE270	Repair	EME1	SNV	chr17	48456028	691	99.1	c.976G>A	p.Asp326Asn
KYSE270	Repair	ERCC5	SNV	chr13	103506711	470	32.6	c.1816G>A	p.Glu606Lys
KYSE270	Repair	ERCC6	SNV	chr10	50678425	256	100.0	c.3581A>G	p.Glu1194Gly
KYSE270	Repair	PARP2	SNV	chr14	20819268	892	99.8	c.523A>G	p.Ile175Val
KYSE270	SWI/SNF	PHF10	SNV	chr6	170110427	1106	39.2	c.1018T>A	p.Phe340Ile
KYSE270	Repair	RTEL1	SNV	chr20	62319357	3910	18.9	c.1621C>T	p.Pro541Ser
KYSE270	Repair	RTEL1	SNV	chr20	62326159	516	63.0	c.3247G>A	p.Ala1083Thr
KYSE270	SWI/SNF	SMARCA4	SNV	chr19	11105565	2255	21.4	c.1481C>G	p.Thr494Arg
KYSE410	Repair	ATR	SNV	chr3	142274923	1234	16.0	c.2137C>G	p.Gln713Glu
KYSE410	Repair	BLM	SNV	chr15	91354521	758	50.7	c.3961G>A	p.Val1321Ile
KYSE410	Repair	ERCC2	Deletion	chr19	45873426..45873428	1722	24.3	c.68_70delCCT	p.Ser23_Tyr24delinsTyr
KYSE410	CP1	KRAS	SNV	chr12	25398285	7095	35.1	c.34G>T	p.Gly12Cys
KYSE410	Repair	LIG4	SNV	chr13	108863609	1930	99.6	c.8C>T	p.Ala3Val
KYSE410	Repair	PRKDC	SNV	chr8	48710955	5872	55.0	c.10298T>C	p.Ile3433Thr
KYSE410	Repair	PRKDC	SNV	chr8	48719852	5217	49.5	c.9592C>G	p.Pro3198Ala
KYSE410	SWI/SNF	SMARCA4	SNV	chr19	11144038	756	50.1	c.3619G>A	p.Val1207Ile
KYSE410	CP1	TP53	SNV	chr17	7574018	7772	99.4	c.1009C>T	p.Arg337Cys
KYSE450	SWI/SNF	ARID1A	Deletion	chr1	27102087..27102108	476	31.3	c.5013_5034delGGTAATGATGTCCCTCAAGTCT	p.Met1673fs
KYSE450	Repair	BLM	SNV	chr15	91354521	1578	71.1	c.3961G>A	p.Val1321Ile
KYSE450	Repair	BRCA1	SNV	chr17	41223048	2465	33.8	c.4946T>C	p.Met1649Thr
KYSE450	CP1	EGFR	SNV	chr7	55249005	27716	22.3	c.2303G>T	p.Ser768Ile
KYSE450	Repair	MDC1	SNV	chr6	30671868	653	33.7	c.5092C>G	p.Pro1698Ala
KYSE450	Repair	PRKDC	SNV	chr8	48711777	705	99.4	c.10290G>T	p.Met3430Ile
KYSE450	Repair	RTEL1	SNV	chr20	62326159	681	81.1	c.3247G>A	p.Ala1083Thr
KYSE450	CP1	SMAD4	SNV	chr18	48591888	7712	98.7	c.1051G>A	p.Asp351Asn
KYSE450	CP1	TP53	SNV	chr17	7574012	24099	36.3	c.1015G>T	p.Glu339*
KYSE450	CP1	TP53	SNV	chr17	7578394	16287	68.8	c.536A>G	p.His179Arg
KYSE510	Repair	BRCA2	SNV	chr13	32912619	1360	26.7	c.4127G>A	p.Gly1376Glu
KYSE510	Repair	BRCA2	SNV	chr13	32913044	1197	28.7	c.4552G>C	p.Glu1518Gln
KYSE510	CP1	EGFR	SNV	chr7	55241657	15823	29.7	c.2105C>A	p.Ala702Asp
KYSE510	Repair	MDC1	SNV	chr6	30672954	208	12.5	c.4006A>G	p.Thr1336Ala
KYSE510	CP1	PIK3CA	SNV	chr3	178936091	20440	24.8	c.1633G>A	p.Glu545Lys
KYSE510	Repair	RBBP8	SNV	chr18	20573579	3921	14.2	c.1789G>C	p.Glu597Gln
KYSE510	Repair	RTEL1	SNV	chr20	62326159	412	61.9	c.3247G>A	p.Ala1083Thr
KYSE510	CP1	TP53	SNV	chr17	7574000	24453	49.8	c.1027G>T	p.Glu343*
KYSE520	Repair	LIG4	SNV	chr13	108863609	1538	99.8	c.8C>T	p.Ala3Val

* Coverage was less than 100, but this mutation was exceptionally confirmed by Sanger sequencing.
